# Infant circulating MicroRNAs as biomarkers of effect in fetal alcohol spectrum disorders

**DOI:** 10.1038/s41598-020-80734-y

**Published:** 2021-01-14

**Authors:** Amanda H. Mahnke, Georgios D. Sideridis, Nihal A. Salem, Alexander M. Tseng, R. Colin Carter, Neil C. Dodge, Aniruddha B. Rathod, Christopher D. Molteno, Ernesta M. Meintjes, Sandra W. Jacobson, Rajesh C. Miranda, Joseph L. Jacobson

**Affiliations:** 1 Department of Neuroscience and Experimental Therapeutics, Texas A&M University Health Science Center, Bryan, TX 77807 USA; 2 Women’s Health in Neuroscience Program, Texas A&M University Health Science Center, Bryan, TX 77807 USA; 3 Harvard Medical School, Boston Children’s Hospital, Institutional Centers for Clinical and Translational Research, Boston, MA 02115 USA; 4 Departments of Pediatrics and Emergency Medicine, Institute of Human Nutrition, Columbia University Medical Center, New York, NY 10032 USA; 5 Department of Psychiatry and Behavioral Neurosciences, Wayne State University School of Medicine, Detroit, MI 48201 USA; 6 Departments of Human Biology and of Psychiatry and Mental Health, Faculty of Health Sciences, University of Cape Town, Cape Town, South Africa; 7 Division of Biomedical Engineering, Department of Human Biology, Faculty of Health Sciences, University of Cape Town, Cape Town, South Africa

**Keywords:** Biomarkers, miRNAs, Neurodevelopmental disorders

## Abstract

Prenatal alcohol exposure (PAE) can result in cognitive and behavioral disabilities and growth deficits. Because alcohol-related neurobehavioral deficits may occur in the absence of overt dysmorphic features or growth deficits, there is a need to identify biomarkers of PAE that can predict neurobehavioral impairment. In this study, we assessed infant plasma extracellular, circulating miRNAs (_*ex*_miRNAs) obtained from a heavily exposed Cape Town cohort to determine whether these can be used to predict PAE-related growth restriction and cognitive impairment. PAE, controlling for smoking as a covariate, altered 27% of expressed _*ex*_miRNAs with clinically-relevant effect sizes (Cohen’s *d* ≥ 0.4). Moreover, at 2 weeks, PAE increased correlated expression of _*ex*_miRNAs across chromosomes, suggesting potential co-regulation. In confirmatory factor analysis, the variance in expression for PAE-altered _*ex*_miRNAs at 2 weeks and 6.5 months was best described by three-factor models. Pathway analysis found that factors at 2 weeks were associated with (F1) cell maturation, cell cycle inhibition, and somatic growth, (F2) cell survival, apoptosis, cardiac development, and metabolism, and (F3) cell proliferation, skeletal development, hematopoiesis, and inflammation, and at 6.5 months with (F1) neurodevelopment, neural crest/mesoderm-derivative development and growth, (F2) immune system and inflammation, and (F3) somatic growth and cardiovascular development. Factors F3 at 2 weeks and F2 at 6.5 months partially mediated PAE-induced growth deficits, and factor F3 at 2 weeks partially mediated effects of PAE on infant recognition memory at 6.5 months. These findings indicate that infant _*ex*_miRNAs can help identify infants who will exhibit PAE-related deficits in growth and cognition.

## Introduction

Despite prevention guidelines and public health advisories^1–3^, alcohol use continues to be common during pregnancy. A recent meta-analysis determined the global prevalence of alcohol use during pregnancy of 9.8%^4^. In the US, a 2013 report found that ~ 18% of women consumed alcohol during pregnancy, and 6.6% reported binge-drinking episodes^5^, which can be particularly damaging to fetal development^6^. In a 2015–2016 Texas state-wide assessment, we reported a third trimester rate of alcohol exposure of 8.4%, with rates as high as 17.7% in some geographical regions^7^. Global prevalence estimates for fetal alcohol syndrome (FAS) range from 0.15 to 0.3%^4, 8^ but are much higher in endemic populations, such as, 5.9–9.1% in the Cape Coloured (mixed ancestry) community in South Africa^9^. The diagnosis of FAS, the most severe of the fetal alcohol spectrum disorders (FASD), consists of a specific pattern of facial dysmorphology, microcephaly, and growth retardation, and applies to only a fraction of the estimated population of fetal alcohol-affected children^10, 11^. Estimates of the prevalence of FASD range from ~ 1 to 5% of the school age population in the US^12^ to 13.6–20.9% in the Western Cape Province of South Africa^9^, and the public health and economic burdens attributable to FASD are substantial^13^.

Early identification of alcohol-affected children can facilitate early intervention, which can mitigate some of the adverse secondary effects of prenatal alcohol exposure (PAE), which may emerge later in life^14^. However, identification of alcohol-affected children is difficult, particularly in children who lack the characteristic dysmorphic facial features that characterize FAS and partial FAS (PFAS)^15^. PAE-related dysmorphology is especially difficult to identify in infancy and early childhood, when interventions may be particularly effective^16^. A documented history of drinking during pregnancy is often difficult to obtain^9^, requiring detailed interviewing by trained and skilled interviewers^17, 18^. To date, several promising *biomarkers of exposure* to alcohol in utero have been identified, including fatty acid ethyl esters, which are metabolites of alcohol that are found in neonatal meconium^19–21^, ethyl glucuronide in placenta^22^, and phosphatidylethanol in newborn blood^23^. However, because effects of PAE on cognition and behavior vary considerably depending on factors, including timing of exposure, dose per drinking occasion, and genetic vulnerability, biomarkers of exposure cannot identify which exposed children will be adversely affected and require intervention.

A recent study on children from our original Cape Town Longitudinal Cohort^24^, using facial imaging involving dense surface modeling and shape signature analysis of 3 dimensional (3D) facial photographs, provided one of the first *biomarkers of effect* for FASD^25^. The 3D methodology showed that about half of the heavily exposed (HE) nonsyndromal children who appeared to lack the distinctive facial anomalies when examined by dysmorphologists, actually had subtle, difficult-to-detect facial features, resembling those seen in FAS and PFAS, and that these children had deficits in verbal IQ and learning and memory comparable to those in FAS and PFAS. These sophisticated facial imaging procedures are not yet available and cost-efficient for routine clinical practice. There is, therefore, a need for alternative, less expensive biomarkers of effect that can identify which exposed nonsyndromal children may be developmentally compromised by PAE^26^.

Data from a preclinical study^27^ and two previous human studies^28, 29^ suggest that microRNAs (miRNAs) secreted by cells and tissues into biofluids, such as blood, can provide biochemically stable, cost-effective biomarkers of PAE. miRNAs are a class of small non-protein-coding RNAs that, among several functions, serve as intracellular repressors of protein translation^30^. In preclinical models, our research was the first to show that ethanol influences miRNA expression^31^ via receptor mediated pathways^31, 32^ and epigenetic mechanisms^33, 34^. We and others have also found that alterations in miRNAs can mediate effects of alcohol on fetal neural stem cells^31, 35^, cranial development^33^, and behavior^36^. A growing body of evidence has shown that miRNAs play an important role in alcohol addiction, toxicity, and teratology^37^. The potential of miRNAs as biomarkers first became evident in 2008 when it was reported that they are secreted into human plasma^38^ and can identify patients with prostate cancer^39^. Impressively, *plasma miRNAs* are remarkably stable with proper sample collection and storage, even through multiple sample freeze–thaw cycles^39^, a factor that enhances their biomarker potential.

Since health care providers are often presented with newborns and infants who lack the characteristic facial dysmorphic features and/or growth deficits but may be alcohol-affected^40, 41^, it is of interest to determine whether biomarkers, such as extracellular, circulating miRNAs (_*ex*_miRNAs) in the presenting infant, can be used to identify alcohol-affected infants; that is, those who will exhibit PAE-related developmental impairment. To address this question, we first identified miRNAs in plasma samples from infants at 2 weeks and 6.5 months that discriminate between alcohol-exposed and non-exposed infants. This study is the first to examine whether _*ex*_miRNAs obtained from the infant can serve as biomarkers both of PAE and of adverse effects that emerge during development following PAE. This study is also the first to test directly whether _*ex*_miRNAs that discriminate between exposed and non-exposed infants mediate effects of PAE on specific outcome domains, namely, postnatal growth restriction and cognition.

## Results

### Participant characteristics

Demographic and background characteristics are summarized in Table 1. The mothers were poorly educated (i.e., the majority did not complete high school; none received any post-high school education) and economically disadvantaged (mean = low end of Hollingshead Inventory^42^ Level IV, semi-skilled laborers). There were no between-group differences in maternal education, socioeconomic or marital status, or parity, nor were there differences in gestational weight gain or daily caloric intake, indicating comparable between-group nutritional status during pregnancy. Women in the control group were on average 3.3 years younger and had their first antenatal visit on average 3.2 weeks earlier than alcohol-exposed mothers.

**Table 1:** Sample characteristics.

	Controls (*n* = 31)			PAE (*n* = 37)				
	Mean or %	SD	Range	Mean or %	SD	Range	*t* or *c*^2^	*p*-value
**Maternal characteristics**								
Age at delivery (year)	25	4.9	18.0 to 36.7	28.3	6.1	19.1 to 43.1	2.47	0.016
Education (year)	9.7	2.2	1.0 to 12.0	9.6	1.5	6.0 to 12.0	0.29	0.776
Socioeconomic status^#^	23.7	7.3	8.0 to 37.0	22.7	7.2	9.5 to 50.0	0.59	0.557
Marital status (% married)	51.6			37.8			1.3	0.255
Parity	2.1	2.1	1 to 5	2.4	2.3	1 to 7	0.97	0.336
Gestational weight gain (kg)	0.5	0.3	− 0.3 to 1.1	0.4	0.4	− 0.7 to 1.3	0.35	0.729
Average daily caloric intake (kJ)	9843.40	3198.1	3706.8 to 20,308.9	9427.60	3058	2660.2 to 15,783.4	0.54	0.589
Pregnancy alcohol use†								
At conception								
AA/day	0.002	0.01	0.0 to 0.1	1.4	0.9	0.0 to 3.2	9.69	< 0.001
AA/occasion	0.1	0.3	0.0 to 1.7	4.5	2.7	0.0 to 9.8	9.9	< 0.001
Frequency (day/week)	0.01	0.1	0.0 to 0.3	2.1	1	0.0 to 4.0	12.8	< 0.001
Across pregnancy								
AA/day	0.001	0.002	0.0 to 0.01	0.7	0.5	0.1 to 2.4	8.1	< 0.001
AA/occasion	0.2	0.5	0.0 to 1.3	4.5	2.3	0.7 to 11.9	11.3	< 0.001
Frequency (day/week)	0.004	0.01	0.0 to 0.1	1.2	0.7	0.1 to 3.0	9.71	< 0.001
Pregnancy cigarettes/day	3.3	4	0.0 to 16.7	5	4.7	0.0 to 20.0	1.55	0.126
Pregnancy marijuana (day/month)	0.5	2.5	0.0 to 14.0	1.2	4.7	0.0 to 21.0	0.69	0.491
**Infant characteristics**								
Sex (% male)	54.8			45.9			0.53	0.465
Gestational age at 1st antenatal visit (week)	27.6	4.9	14.1 to 34.6	24.4	5.7	9.4 to 38.4	2.38	0.020
Gestational age at delivery (week)	39.2	1.9	33.7 to 41.6	38.9	1.9	30.9 to 42.0	0.7	0.489
Birth weight (g)	3151	543.2	1940 to 4400	3016.50	578.6	2220 to 4900	0.98	0.330
Birth length (cm)	48.9	3.3	40.0 to 54.0	48.8	2.7	43.0 to 53.0	0.18	0.855
Birth head circumference (cm)	33.9	1.8	31.0 to 38.0	33.3	1.5	30.0 to 36.0	1.47	0.147
Age at 2-wk visit (month)	0.6	0.3	0.2 to 1.5	0.5	0.3	0.1 to 1.4	1.16	0.251
Age at 6.5-month visit (month)	6.6	0.4	5.7 to 8.0	6.5	0.3	5.8 to 7.1	1.17	0.244
Fagan novelty preference (%)	61	6.7	43.6 to 72.8	60.5	5.9	46.9 to 71.29	0.3	0.765
6.5-month WHO growth z-score								
Weight-for-age	0.3	0.8	− 1.64 to 1.57	− 0.8	1	− 2.2 to 1.43	4.66	< 0.001
Length-for-age	− 0.4	1.1	− 3.3 to 2.31	− 1.2	1	− 3.3 to 0.6	3.25	0.002
Head circumference-for-age	-0.1	0.8	− 2.09 to 1.45	− 0.9	0.8	− 2.3 to 0.5	4.40	< 0.001

SD, Standard deviation; AA, absolute alcohol measured in ounces; WHO, World Health Organization.

^#^Based on Hollingshead Four Factor Index of Social Status Scale^42^.

^†^One ounce is equivalent to 28 g or 30 ml absolute alcohol.

The mothers of infants in the alcohol-exposed group reported consuming an average of 4.5 oz absolute alcohol (AA)/occasion (128 g; 133 ml; ≈ 9 standard drinks/occasion) across pregnancy on an average of 1–2 days/week. 26 of the 31 controls (83.9%) abstained from drinking during the pregnancy. Four controls (12.9%) reported no alcohol use when recruited but subsequently drank 1–2.5 drinks on 1–2 occasions later in pregnancy; the fifth non-abstaining control consumed 3 drinks on 2 occasions around time of conception and then reported abstaining when she learned she was pregnant. While there were no group differences in number of cigarettes smoked/day, 31 women (78.4%) reported smoking in the PAE group compared to 17 controls (54.8%) (*Χ*^2^(1) = 4.27, *p* = 0.039). The number of cigarettes smoked/day was generally low with most women smoking < 0.5 pack/day. Only 4 women (10.8%) reported using marijuana in the PAE group and 2 among the controls (6.5%) (*Χ*^2^(1) = 0.40, *p* = 0.528); the number of days/week marijuana was used did not differ between the two groups. None of the women reported using methamphetamine, methaqualone, cocaine, or opiates during pregnancy.

There were no between-group differences in infant sex, gestational age (GA) at birth, birth weight, length, head circumference, or age at T_2wk_ or T_6.5mo_, but the alcohol-exposed infants had smaller weight-, length-, and head circumference-for-age by 6.5 months of age. Five of the 37 infants (13.5%) born to the heavy drinking mothers met the Revised Institute of Medicine criteria^10^ for full FAS; an additional two (5.9%), for PFAS.

### Plasma and RNA characteristics

#### Sample purity characteristics

There were no significant differences in free hemoglobin levels (absorbance at 414 nm) attributable to exposure group or infant sex (both *p*-values > 0.20). Free hemoglobin at 2 weeks was 5.4-fold higher than at 6.5 months (*F*_(1,118)_ = 38.93, *p* = 7.11 × 10^–09^; see Supplementary Fig. S1). However, there were no significant differences due to age, exposure group, or sex in the difference in cycle threshold (ΔCT) for miR-23a-3p (miRbase accession number MIMAT0000078) and miR-451a (MIMAT0001631) (ΔCT_(miR23a-miR451a)_), an independent marker for hemolysis^43^. Moreover, in contrast to previous reports in adult populations^44^, there was no significant relation (*r* = 0.13, *p* > 0.10) between absorbance at 414 nm and ΔCT_(miR23a-miR451a)_, and none of the samples expressed the erythrocyte-specific transcript, SLC4A1 (see Supplementary Fig. S1). These data suggest that the elevated free hemoglobin observed was not due to acute hemolysis during sample collection but rather to the normal physiologic elimination of extra red blood cells that occurs during the first ~ 6 weeks of life.

**Figure 1:**
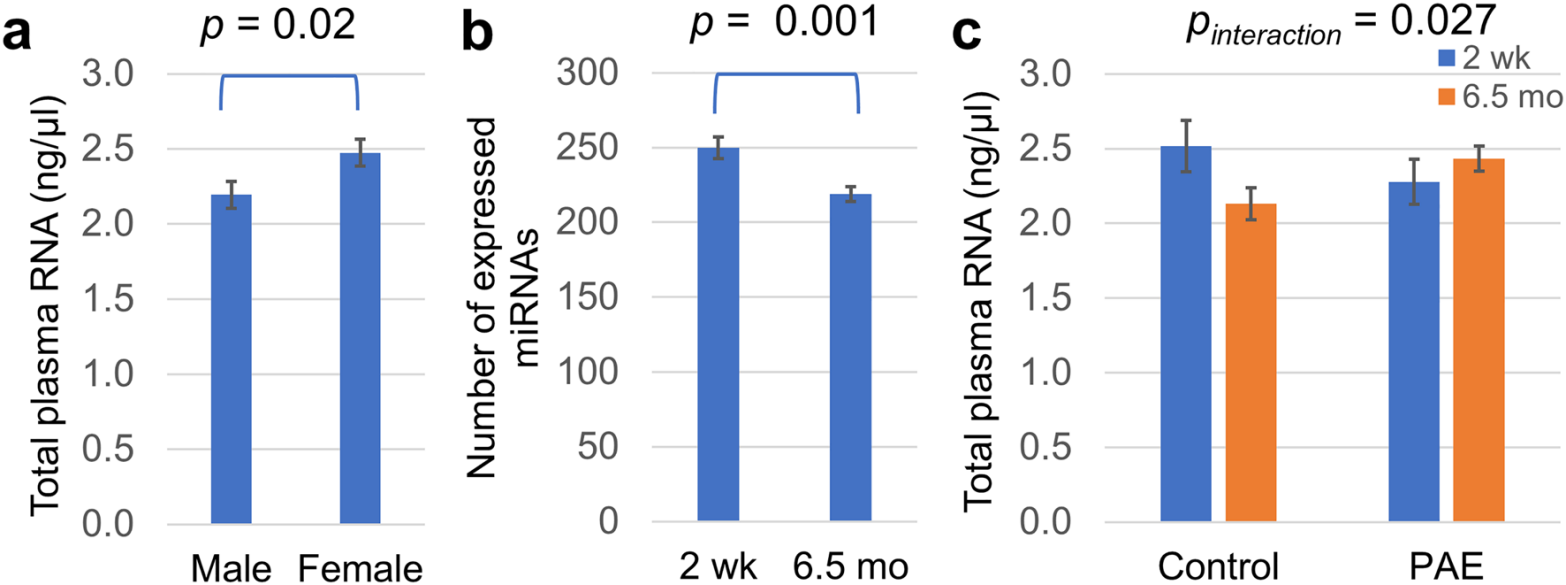
RNA and miRNA content in infant plasma samples. (**a**) Female and male assessed plasma RNA content, determined by multiplying isolated RNA concentration by total plasma volume. (**b**) The total number of expressed plasma miRNAs, i.e., miRNAs with detected CT, at T_2wk_ and T_6.5mo_. (**c**) Association between plasma RNA concentration, PAE status, and infant age. For (**a**) and (**b**), *t*-test *p*-values are shown. For (**c**), the *p*-value shown is for the interaction between age and exposure group resulting from a two-way ANOVA.

*Sex and age effects.* Female infant-derived plasma samples contained ~ 12% more total isolated RNA, which included carrier MS2 RNA, per microliter (Fig. 1a) compared to male samples (*F*_(1,118)_ = 5.6, *p* = 0.02). The number of expressed miRNAs decreased with age (*F*_(1,110)_ = 12.9, *p* = 0.001; Fig. 1b); infants expressed ~ 14% fewer unique miRNAs at 6.5 months than at 2 weeks. However, whereas the control infants exhibited an ~ 18% decline in total RNA content between T_2wk_ and T_6.5mo_, infants with PAE continued to exhibit elevated plasma RNA levels at T_6.5mo_ (age by exposure group interaction: *F*_(1,118)_ = 4.99, *p* = 0.027; Fig. 1c). Notwithstanding, there were no significant effects of PAE or infant sex on number of unique miRNAs expressed (both *p*-values > 0.20) and no effects of PAE, age, or sex on average expression level of all expressed miRNAs (average CT; all *p*-values > 0.15).

### PAE influences _ex_miRNA expression in infancy

**Figure 2:**
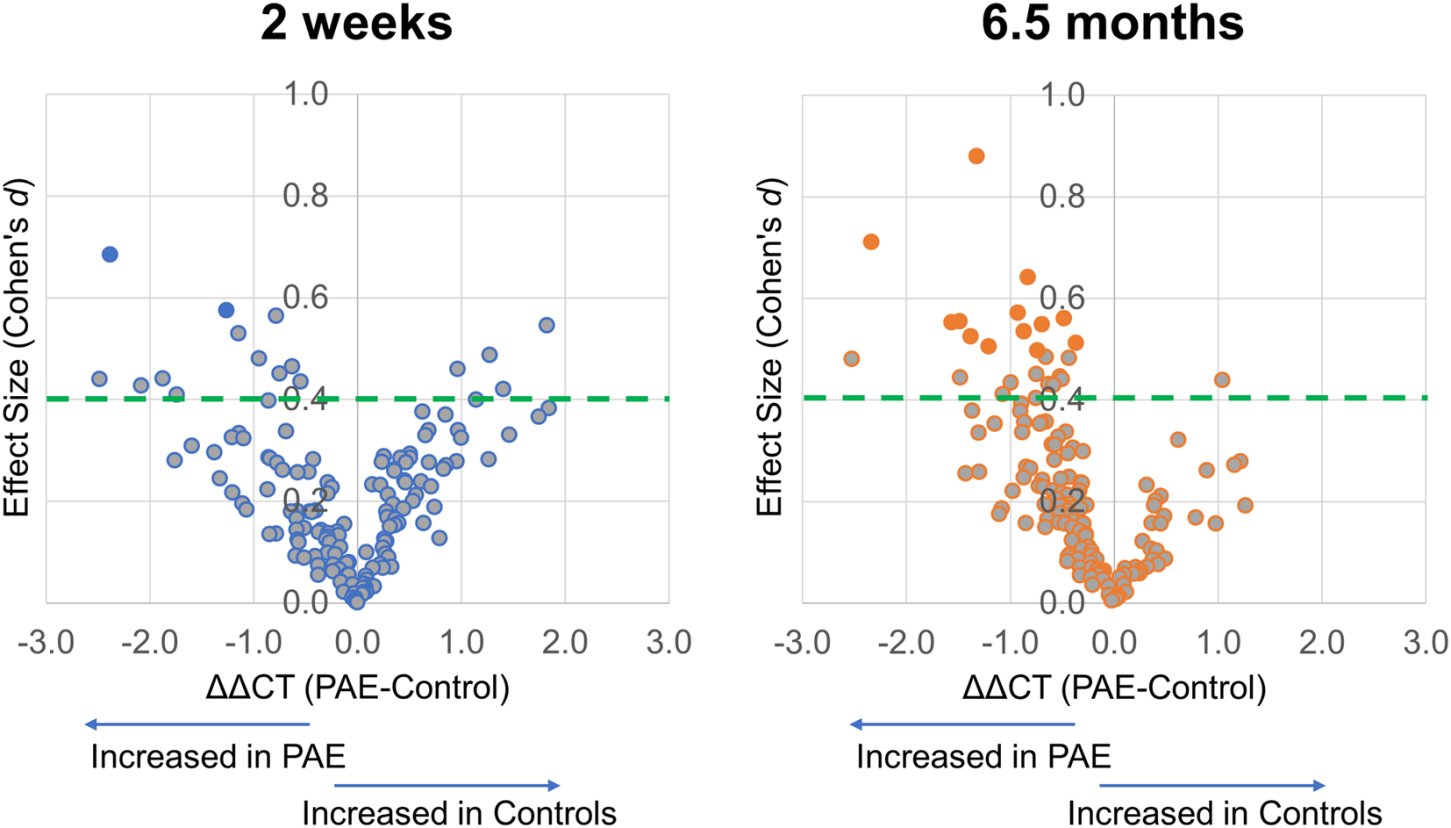
PAE-altered _*ex*_miRNAs in infant plasma. Volcano plots of relative expression of _*ex*_miRNAs (ΔΔCT, ΔCT_PAE_—ΔCT_control_) and effect size (Cohen’s *d*) at T_2wk_ (left) andT_6.5mo_ (right). Dashed green line denotes a clinically relevant, moderate effect size of 0.40. Orange and blue filled points denote _*ex*_miRNAs with significant effect sizes (95% confidence interval does not contain zero).

We next examined the 148 miRNAs that were detected in at least 80% of the samples in either the alcohol-exposed or control group at T_2wk_ or T_6.5mo_ (Table 2, Supplementary Table S2). To isolate the effects of PAE in this population, effect sizes were determined based on ANCOVA adjusted means, with cigarettes/day as the covariate. The expression of two miRNAs at T_2wk_ and 13 miRNAs at T_6.5mo_ was significantly altered by PAE, i.e., the 95% confidence interval of the effect size did not contain zero (ANCOVA *F* > 4.03; *p* < 0.049). To create a broader set of candidate miRNAs that might be present in miRNA profiles with mechanistic significance for FASD in this sample, we next identified miRNAs that differed between the exposed and control groups with at least a medium effect size (Cohen’s *d* ≥ 0.40). At T_2wk_, expression levels of 18 miRNAs differed between the exposed and control groups, with effect sizes ranging from 0.40 (*p* = 0.179) to 0.68 (*p* = 0.014). At T_6.5mo_, expression of 26 miRNAs differed between the groups with effect sizes ranging from 0.40 (*p* = 0.105) to 0.88 (*p* = 0.001). At T_2wk_, 72% of these PAE-responsive miRNAs were upregulated by PAE, while at T_6.5mo,_ 92% of the miRNAs were upregulated by PAE (Fig. 2).

**Table 2:** _*ex*_miRNA expression and effect size.

MIMAT #	miRNA	ΔCT(SD)		All Samples		Girls		Boys	
		Controls	Exposed	*d*	95% CI	*g*	95% CI	*g*	95% CI
**2 wk**									
MIMAT0005867^a^	hsa-miR-663b	1.2 (3.5)	− 1.2 (3.5)	0.68	[0.14, 1.23]*	0.95	[0.17, 1.73]*	0.29	[− 0.47, 1.05]
MIMAT0006764^a^	hsa-miR-320d	0.7 (2.2)	− 0.6 (2.2)	0.57	[0.03, 1.12]*	0.91	[0.13, 1.68]*	0.06	[− 0.70, 0.82]
MIMAT0000244^b^	hsa-miR-30c-5p	1.9 (1.4)	1.1 (1.4)	0.56	[− 0.03, 1.15]	0.92	[0.12, 1.73]*	0.06	[− 0.79, 0.92]
MIMAT0000443^b^	hsa-miR-125a-5p	2.4 (3.4)	4.3 (3.3)	− 0.55	[− 1.13, 0.04]	− 0.23	[− 1.00, 0.54]	− 0.96	[− 1.87, − 0.06]*
MIMAT0000076^b^	hsa-miR-21-5p	− 2.6 (2.2)	− 3.7 (2.2)	0.53	[− 0.06, 1.12]	0.73	[− 0.06, 1.52]	0.34	[− 0.52, 1.20]
MIMAT0000070^b^	hsa-miR-17-5p	2.7 (2.6)	4.0 (2.6)	− 0.49	[− 1.07, 0.10]	− 0.35	[− 1.12, 0.42]	− 0.66	[− 1.54, 0.22]
MIMAT0000435^b^	hsa-miR-143-3p	1.7 (2.0)	0.8 (2.0)	0.48	[− 0.11, 1.07]	0.37	[− 0.40, 1.15]	0.60	[− 0.27, 1.48]
MIMAT0000077^b^	hsa-miR-22-3p	0.1 (1.4)	− 0.6 (1.3)	0.46	[− 0.12, 1.05]	0.53	[− 0.26, 1.31]	0.31	[− 0.55, 1.18]
MIMAT0000707^b^	hsa-miR-363-3p	− 0.8 (2.1)	0.1 (2.1)	− 0.46	[− 1.05, 0.13]	− 0.31	[− 1.09, 0.46]	− 0.65	[− 1.53, 0.23]
MIMAT0009447^a^	hsa-miR-1972	1.6 (1.7)	0.8 (1.7)	0.45	[− 0.09, 0.99]	0.24	[− 0.50, 0.99]	0.76	[− 0.03, 1.54]
MIMAT0003293^a^	hsa-miR-624-5p	5.4 (4.3)	3.5 (4.2)	0.44	[− 0.10, 0.98]	0.61	[− 0.14, 1.37]	0.18	[− 0.58, 0.95]
MIMAT0002891^b^	hsa-miR-18a-3p	8.4 (5.7)	5.9 (5.6)	0.44	[− 0.15, 1.02]	0.55	[− 0.23, 1.33]	0.26	[− 0.60, 1.12]
MIMAT0000092^b^	hsa-miR-92a-3p	− 4.4 (1.3)	− 4.9 (1.2)	0.43	[− 0.15, 1.02]	− 0.25	[**-**1.02, 0.52]	0.98	[0.08, 1.89]*
MIMAT0003266^b^	hsa-miR-598-3p	8.2 (4.9)	6.1 (4.9)	0.43	[**-**0.16, 1.01]	0.40	[**-**0.37, 1.18]	0.33	[**-**0.53, 1.20]
MIMAT0000093^b^	hsa-miR-93-5p	− 4.0 (3.4)	− 2.6 (3.3)	− 0.42	[− 1.00, 0.16]	− 0.28	[− 1.05, 0.49]	− 0.54	[− 1.41, 0.33]
MIMAT0004496^a^	hsa-miR-23a-5p	8.4 (4.3)	6.7 (4.2)	0.41	[− 0.13, 0.95]	0.32	[− 0.42, 1.06]	0.45	[− 0.32, 1.21]
MIMAT0000728^b^	hsa-miR-375-3p	2.8 (2.9)	4.0 (2.9)	− 0.40	[− 0.98, 0.18]	− 0.29	[− 1.06, 0.48]	− 0.51	[− 1.38, 0.36]
MIMAT0000692^b^	hsa-miR-30e-5p	− 0.2 (2.2)	− 1.1 (2.2)	0.40	[− 0.19, 0.98]	0.12	[− 0.65, 0.89]	0.63	[− 0.25, 1.51]
**6.5 mo**^c^									
MIMAT0000445	hsa-miR-126-3p	− 2.1 (1.5)	− 3.4 (1.5)	0.88	[0.37, 1.39]*	0.89	[0.18, 1.61]*	0.75	[0.04, 1.45]*
MIMAT0000752	Has-miR-328-3p	4.3 (3.3)	2.0 (3.3)	0.71	[0.21, 1.21]*	1.15	[0.42, 1.89]*	0.34	[− 0.35, 1.03]
MIMAT0000244	hsa-miR-30c-5p	1.7 (1.3)	0.8 (1.3)	0.64	[0.14, 1.14]*	0.86	[0.15, 1.58]*	0.33	[− 0.35, 1.02]
MIMAT0000080	hsa-miR-24-3p	− 2.0 (1.6)	-3.0 (1.6)	0.57	[0.08, 1.07]*	0.30	[-0.39, 0.98]	0.72	[0.02, 1.43]*
MIMAT0000449	hsa-miR-146a-5p	− 0.5 (0.9)	1.0 (0.9)	0.56	[0.06, 1.06]*	0.83	[0.12, 1.54]*	0.22	[− 0.47, 0.90]
MIMAT0004614	hsa-miR-193a-5p	5.5 (2.7)	4.0 (2.7)	0.55	[0.06, 1.05]*	0.62	[− 0.08, 1.32]	0.45	[− 0.24, 1.14]
MIMAT0000101	hsa-miR-103a-3p	0.1 (2.8)	− 1.4 (2.8)	0.55	[0.06, 1.05]*	0.70	[0.00, 1.41]*	0.36	[− 0.32, 1.05]
MIMAT0000092	hsa-miR-92a-3p	− 4.8 (1.3)	− 5.5 (1.3)	0.55	[0.05, 1.04]*	0.94	[0.22, 1.66]*	0.07	[− 0.61, 0.76]
MIMAT0000435	hsa-miR-143-3p	1.2 (1.6)	0.3 (1.6)	0.53	[0.04, 1.03]*	0.68	[− 0.02, 1.38]	0.37	[− 0.32, 1.06]
MIMAT0000431	hsa-miR-140-5p	2.1 (2.6)	0.7 (2.6)	0.52	[0.03, 1.02]*	0.35	[− 0.34, 1.03]	0.65	[− 0.05, 1.35]
MIMAT0000418	hsa-miR-23b-3p	− 1.1 (0.7)	− 1.4 (0.7)	0.51	[0.02, 1.01]*	0.56	[− 0.14, 1.25]	0.38	[− 0.31, 1.07]
MIMAT0000443	hsa-miR-125a-5p	2.9 (2.4)	1.7 (2.4)	0.50	[0.01, 1.00]*	0.15	[− 0.53, 0.83]	0.89	[0.18, 1.61]*
MIMAT0000460	hsa-miR-194-5p	1.7 (1.5)	1.0 (1.5)	0.50	[0.00, 0.99]*	0.09	[− 0.60, 0.77]	0.71	[0.01, 1.41]*
MIMAT0000420	hsa-miR-30b-5p	1.9 (1.4)	1.2 (1.4)	0.48	[− 0.01, 0.98]	0.92	[0.20, 1.63]*	0.20	[− 0.48, 0.88]
MIMAT0000243	hsa-miR-148a-3p	− 0.3 (0.9)	− 0.8 (0.9)	0.48	[− 0.01, 0.98]	0.37	[− 0.32, 1.06]	0.49	[− 0.20, 1.19]
MIMAT0000069	hsa-miR-16-5p	− 4.4 (5.3)	− 6.9 (5.2)	0.48	[− 0.01, 0.97]	0.82	[0.11, 1.53]*	0.13	[− 0.55, 0.82]
MIMAT0000074	hsa-miR-19b-3p	− 4.4 (1.7)	− 5.1 (1.7)	0.45	[− 0.04, 0.94]	0.42	[− 0.27, 1.11]	0.41	[− 0.28, 1.10]
MIMAT0004748	hsa-miR-423-5p	− 1.6 (1.2)	− 2.1 (1.2)	0.45	[− 0.05, 0.94]	1.17	[0.43, 1.90]*	− 0.11	[− 0.79, 0.57]
MIMAT0000099	hsa-miR-101-3p	0.9 (3.3)	− 0.6 (3.3)	0.44	[− 0.05, 0.94]	0.43	[− 0.26, 1.12]	0.41	[− 0.28, 1.10]
MIMAT0000419	hsa-miR-27b-3p	− 0.8 (1.1)	− 1.3 (1.1)	0.44	[− 0.05, 0.93]	0.89	[0.17, 1.60]*	0.19	[− 0.49, 0.87]
MIMAT0004911	hsa-miR-874-3p	3.1 (2.4)	4.2 (2.4)	− 0.44	[− 0.93, 0.05]	− 0.57	[− 1.26, 0.13]	− 0.36	[− 1.05, 0.32]
MIMAT0002888	hsa-miR-532-5p	2.7 (2.3)	1.7 (2.3)	0.43	[− 0.06, 0.93]	0.23	[− 0.46, 0.91]	0.55	[− 0.14, 1.25]
MIMAT0000078	hsa-miR-23a-3p	− 3.2 (1.5)	− 3.8 (1.5)	0.43	[− 0.06, 0.92]	0.63	[− 0.07, 1.33]	0.14	[− 0.55, 0.82]
MIMAT0000280	hsa-miR-223-3p	− 4.0 (1.4)	− 4.6 (1.4)	0.43	[− 0.06, 0.92]	0.37	[− 0.32, 1.06]	0.52	[− 0.17, 1.22]
MIMAT0000423	hsa-miR-125b-5p	0.0 (2.6)	− 1.0 (2.6)	0.41	[− 0.08, 0.90]	0.56	[− 0.13, 1.26]	0.54	[− 0.15, 1.24]
MIMAT0000087	hsa-miR-30a-5p	3.0 (1.9)	2.3 (1.9)	0.40	[− 0.09, 0.89]	0.77	[0.06, 1.48]*	− 0.13	[− 0.82, 0.55]

MIMAT #, miRBase identification number; ΔCT, average miRNA expression cycle threshold following sample normalization to global mean of miRNA expression, SD, standard deviation; *d,* Cohen's *d* effect size; *g,* Hedges's *g* effect size; CI, confidence interval for effect size measurement.

*Effect size has a non-zero spanning 95% confidence interval.

^a^All Samples: Exposed N = 32, Control N = 24; Girls: Exposed N = 17, Control N = 12; Boys: Exposed N = 15, Control N = 12. ^b^All Samples: Exposed N = 27, Controls N = 21; Girls: Exposed N = 16, Control N = 11; Boys: Exposed N = 11, Control = 10. Samples/panels that did not reach technical criteria for amplification were omitted. ^c^All samples: Exposed N = 36, Control N = 31; Girls: Exposed N = 20, Control N = 14; Boys: Exposed N = 16, Control N = 17.

**Figure 3:**
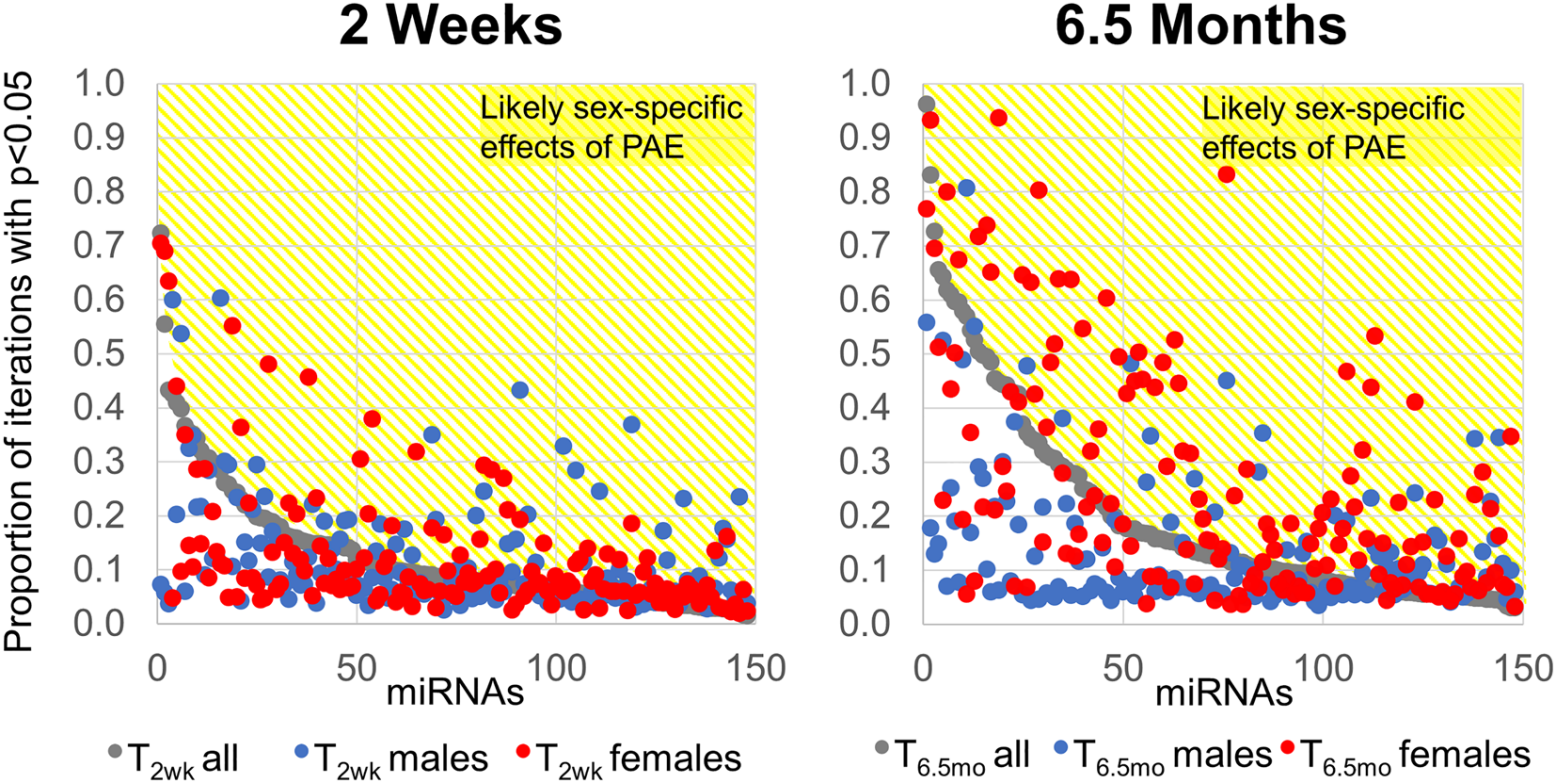
Sexually dimorphic changes in _*ex*_miRNAs in response to PAE. Bootstrap resampling of _*ex*_miRNA expression at T_2wk_ (left) and T_6.5mo_ (right). The population including both male and female samples (gray) was resampled 2000 times with replacement and the proportion of significant *p*-values (ANCOVA with cigarettes/day as a covariate) across the iterations is shown. The population was then resampled with only male (blue) or only female (red) infants. _*ex*_miRNAs that were more likely to be significantly altered when examined in a single sex than in the combined population are in the yellow region and are likely to be altered in a sex-specific manner in response to PAE.

Previous research suggests that there are sex differences in the early presentation of FASD^45^ and some of the cognitive impairments associated with PAE^46^. Moreover, sex differences have been found in the expression of other pediatric biomarkers^47, 48^ and in _*ex*_miRNA profile^49^. Although this study was under-powered to evaluate sex differences in _*ex*_miRNA profiles, some large-effect-size differences did emerge when _*ex*_miRNA expression data were disaggregated by sex (Table 2, Supplementary Table S2). For instance, at T_6.5mo_, MIMAT0000752 (hsa-miR-328-3p) was significantly elevated in female PAE infant plasma samples (Hedges’s *g* = 1.15, *p* = 0.002), but the effect was much smaller in male PAE infants (*g* = − 0.34; *p* = 0.324). Bootstrap resampling analysis^50^ of ANCOVA-adjusted means indicated that the expression of this _*ex*_miRNA was significantly different in the PAE compared to the control group in 83.1% of the resampling iterations which contained both male and female infants (Fig. 3). When resampled separately, female PAE infants were also significantly different from female controls in 93.3% of iterations, whereas male PAE infants were significantly different from male controls in only 17.1% of the iterations. In contrast, MIMAT0000443 (hsa-miR-125a-5p) was significantly elevated in T_6.5mo_ male PAE samples (*g* = 0.89, *p* = 0.013) but not female PAE infant plasma samples (*g* = 0.15; *p* = 0.66). Resampling analysis indicated that the expression of this _*ex*_miRNA was different in PAE compared to the control group in 57.0% of the iterations which contained both male and female infants. However, when resampled separately, male PAE infants were different from controls in 80.7% of the iterations, while female PAE infants were significantly different from controls in only 5.5% of the iterations. Sex differences in PAE-regulated _*ex*_miRNA expression were more pronounced at T_6.5mo_ than at T_2wk_; the number of _*ex*_miRNAs that were significant in more than half of the iterations and more frequently significant than the population as a whole when disaggregated by sex was 6 at T_2wk_ and 21 at T_6.5mo_.

**Figure 4:**
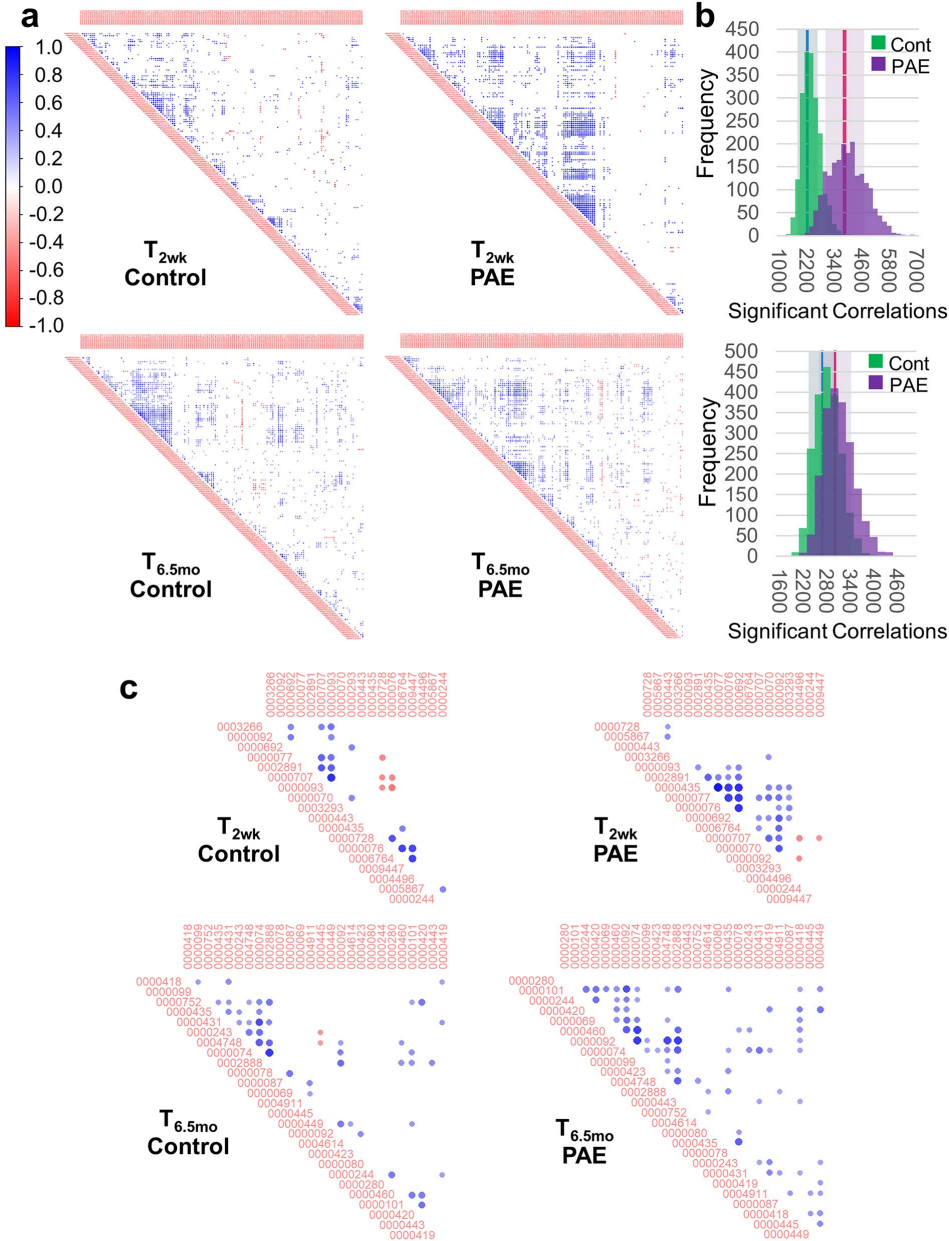
_*ex*_miRNAs are highly correlated in PAE infants at T_2wk_. (**a**) Hierarchically clustered correlation plots of significant (*p* < 0.05) _*ex*_miRNA cross correlations. (**b**) Bootstrap resampling to assess the stability of the number of correlations at T_2wk_ (top)and T_6.5mo_ (bottom). The standard deviation of each distribution are shown by the shaded regions and 99% confidence intervals are shown by the blue (control) and pink (PAE) regions. (**c**) Hierarchically clustered correlation plots of significant (*p* < 0.05) _*ex*_miRNA cross correlations for _*ex*_miRNAs with an effect size ≥ 0.40. Figures (**a**) and (**c**) were constructed using the corrplot package (version 0.77, https://cran.r-project.org/web/packages/corrplot/index.html) for R (version 3.6.1) and figure (b) was constructed using Excel.

Hierarchically-clustered correlation matrices were computed to assess the extent to which PAE resulted in coordinated expression of _*ex*_miRNAs (Fig. 4). At T_2wk_, the alcohol-exposed infants exhibited 1.6-fold greater significantly (*p* < 0.05) correlated _*ex*_miRNAs compared with controls; at T_6.5mo_, the infants with PAE exhibited 1.2-fold more significant correlations as controls. Bootstrap resampling with replacement was used to assess the statistical stability of the number of significant correlations. At both T_2wk_ and T_6.5mo_, PAE was associated with higher numbers of stable correlations among miRNAs compared with controls (99% confidence interval (CI): T_2wk_ Cont [2273.9, 2319.4], PAE [3826.9, 3918.2]; T_6.5mo_ Cont [2708.4, 2746.4], PAE [2999.3, 3043.3]). At T_2wk_ the standard deviation of the control and PAE distributions of significant correlations did not overlap, whereas there was overlap of the distributions at T_6.5mo._ These data indicate that the correlated expression of _*ex*_miRNAs seen at 2 weeks in PAE infants was diminished over development. Re-computation of correlation matrices for the subset of miRNAs that differed between groups with effect size ≥ 0.40 also showed that alcohol-exposed infants exhibited a greater number of positively intercorrelated miRNAs at both ages (T_2wk_: 153 total possible correlations: control = 23 (15%), PAE = 37 (24%); T_6.5mo_: 325 total possible correlations: control = 45 (14%), PAE = 72 (22%)).

### PAE Is associated with increased coordinated expression of _*ex*_miRNAs across chromosomes at 2 weeks

**Figure 5:**
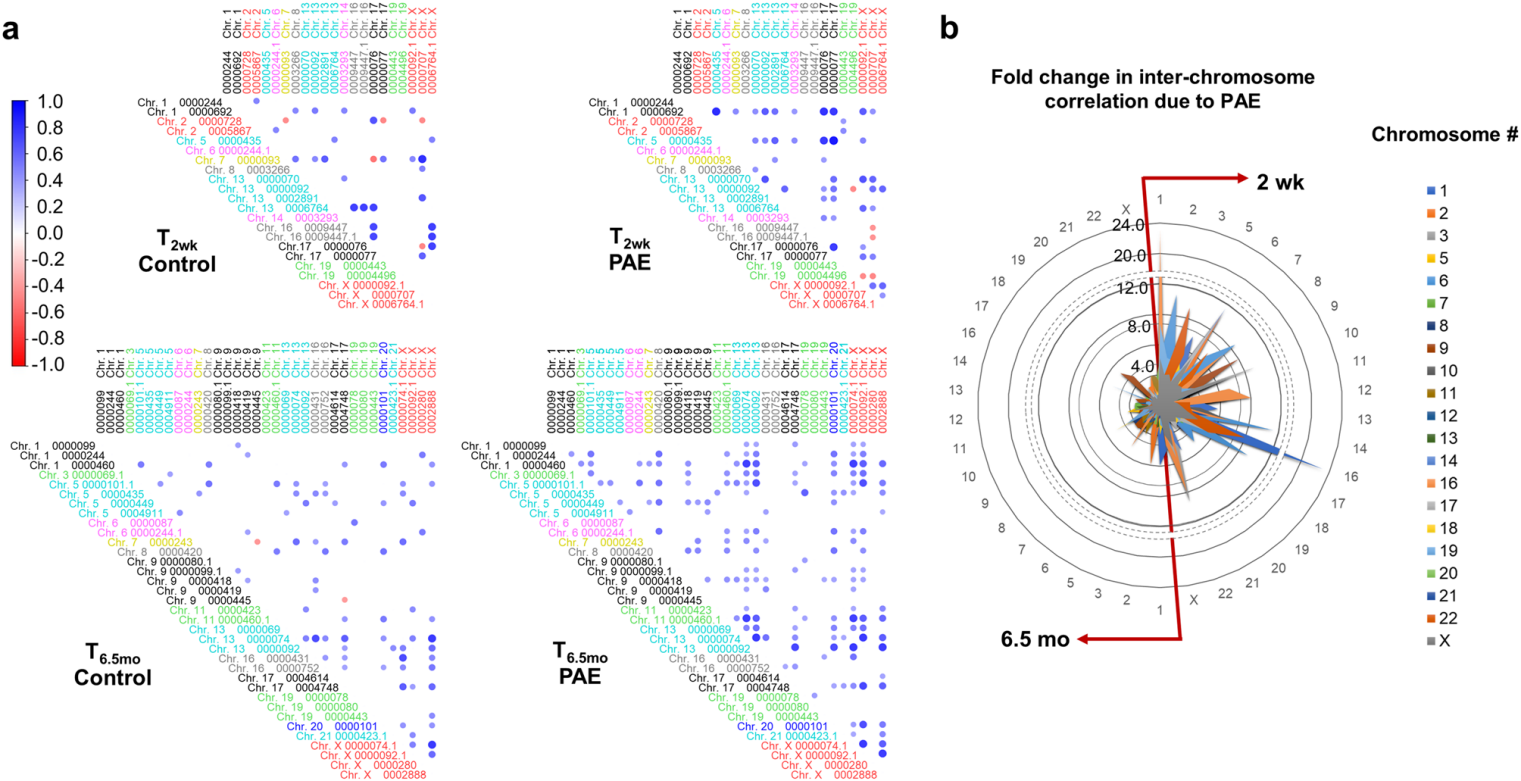
Coordinated expression of _*ex*_miRNAs across chromosomes. (**a**) Cross correlation of _*ex*_miRNA expression, arranged by chromosomal location, at T_2wk_ and T_6.5mo_ for _*ex*_miRNAs with an effect size ≥ 0.40. Individual miRNAs are denoted with MIMAT number and chromosomal location, with “.1” indicative of a chromosomal duplication of the primary miRNA. For corresponding miRNA names, see Table 2. (**b**) From the full correlation matrices (see Supplementary Fig. S3), we assessed the fold-change difference in the number of significant correlations between chromosomes per number of expressed _*ex*_miRNA on each chromosome. Radar plot shows the enrichment of inter-chromosomal correlation in PAE infants compared to control infants at T_2wk_ (right) and T_6.5mo_ (left). Figure (**a**) was constructed using the corrplot package (version 0.77, https://cran.r-project.org/web/packages/corrplot/index.html) for R (version 3.6.1) and figure (**b**) was constructed using Excel.

Transcription is thought to be localized to relatively few spatially distinct nuclear regions, termed transcription factories, where strands from different chromosomes are functionally linked, resulting in co-transcription that can lead to correlated gene expression^51^. To assess the extent to which PAE may reorganize co-transcription from miRNA-encoding chromatin, we tabulated the number of significant correlations between expressed _*ex*_miRNAs by chromosomal location. We then assessed the fold-change associated with PAE in number of significant cross-chromosome correlations between _*ex*_miRNAs. There was a larger number of inter-correlations between _*ex*_miRNAs encoded on different chromosomes in the plasma of exposed infants compared with controls (T_2wk_: 217 total possible cross-chromosomal correlations: control = 32 (15%), PAE = 43 (20%); T_6.5mo_: 560 total possible cross-chromosomal correlations: control = 69 (12%), PAE = 141 (25%)) (Fig. 5; Supplementary Fig. S3). These correlations are not due to co-expression of miRNAs within chromosomal clusters, i.e., miRNAs within 10 kb of additional miRNAs which are commonly co-expressed^52^, as few PAE-sensitive _*ex*_miRNAs were located within chromosomal clusters (Supplementary Table S4). Of note, miRNAs from the development-associated miR 17–92 genomic cluster on chromosome 13^53^, are expressed at T_2wk_ and T_6.5mo_ and have higher correlated expression in the PAE group at both timepoints (percentage of significant correlations T_2wk_: Cont 1.7%, PAE 8.7%; T_6.5mo_: Cont 2.7%, PAE 6.1%). Increased correlation between specific chromosomes was observed (Fig. 5b). For example, at T_2wk_ the number of significant inter-correlations between _*ex*_miRNAs on chromosome 16 and those on chromosome 1 was 23-fold greater in the PAE group than among the controls. Similarly, the number of significant correlations between miRNAs on chromosome 6 and those on chromosomes 2, 13, and 16 was more than tenfold greater in the PAE group than among the controls. Neither of these patterns was seen at T_6.5mo_.

### Confirmatory factor analyses at 2 weeks and 6.5 months of age

An iterative procedure was used to identify clusters of PAE-responsive (Cohen’s *d* ≥ 0.40, adjusted for smoking) _*ex*_miRNAs that shared common variance. Following several iterations in which _*ex*_miRNAs were linked to each other using conceptual (exploratory Ingenuity pathway analysis (IPA)), empirical (chromosomal location), and statistical criteria (exploratory factor analysis), simple factor structures were tested for minimal amounts of measurement error using confirmatory factor analysis (CFA). The biological pathways associated with each of the factors were identified based on IPA. As shown in Table 3, at T_2wk_, a bifactor model provided the best model for clusters of _*ex*_miRNAs that distinguished the exposed and control infants using inferential statistics but not using information criteria (the Bayesian information criterion (BIC) was smaller in the 3-factor correlated solution). Furthermore, the bifactor solution was not interpretable as neither the general factor nor the specific factors were supported. Consequently, the 3-factor correlated solution was the preferred simple structure at both ages.

**Table 3:** Simple structures of miRNAs at neonatal and 6.5-month period and comparison of nested competing models.

Model Tested	*χ*^2^	d.f	*p*-value	Δ *χ*^2^	D.F	AIC	BIC	SABIC
2 Weeks of Age (T_2wk_)								
T_2wk_: Unidimensional	179.058	118	< 0.001***	–	–	3650.815	3756.133	3592.699
*T*_*2wk*_*: 3-factor correlated*	*149.17*	*115*	0.018*	29.888***	*3*	*3626.927*	*3738.321*	*3565.458*
T_2wk_: Bifactor model	110.73	101	0.239	38.440***	14	3616.487	3756.236	3539.372
6.5 Months of Age (T_6.5mo_)								
T_6.5mo_: Unidimensional	332.276	206	< 0.001***	–	–	5612.817	5764.941	5547.685
*T*_*6.5mo*_*: 3-factor correlated*	*274.617*	*203*	0.0002**	57.659***	*3*	*5561.158*	*5719.896*	*5493.194*
T_6.5mo_: Bifactor model	247.454	184	0.013*	27.163***	19	5571.995	5772.622	5486.096

Italics denote chosen model. AIC, Akaike’s information criterion; BIC, Bayesian information criterion; SABIC, sample-size-adjusted Baysian information criterion; *χ*^2^, omnibus chi-squared test *χ*^2^; d.f., degrees of freedom; Δ*χ*^2^, chi-squared difference test; Δ_D.F._, difference of degress of freedom. *P*-values are from the omnibus chi-squared test with significance denoted as *p < 0.05; **p < 0.01; ***p < 0.001.

**Figure 6:**
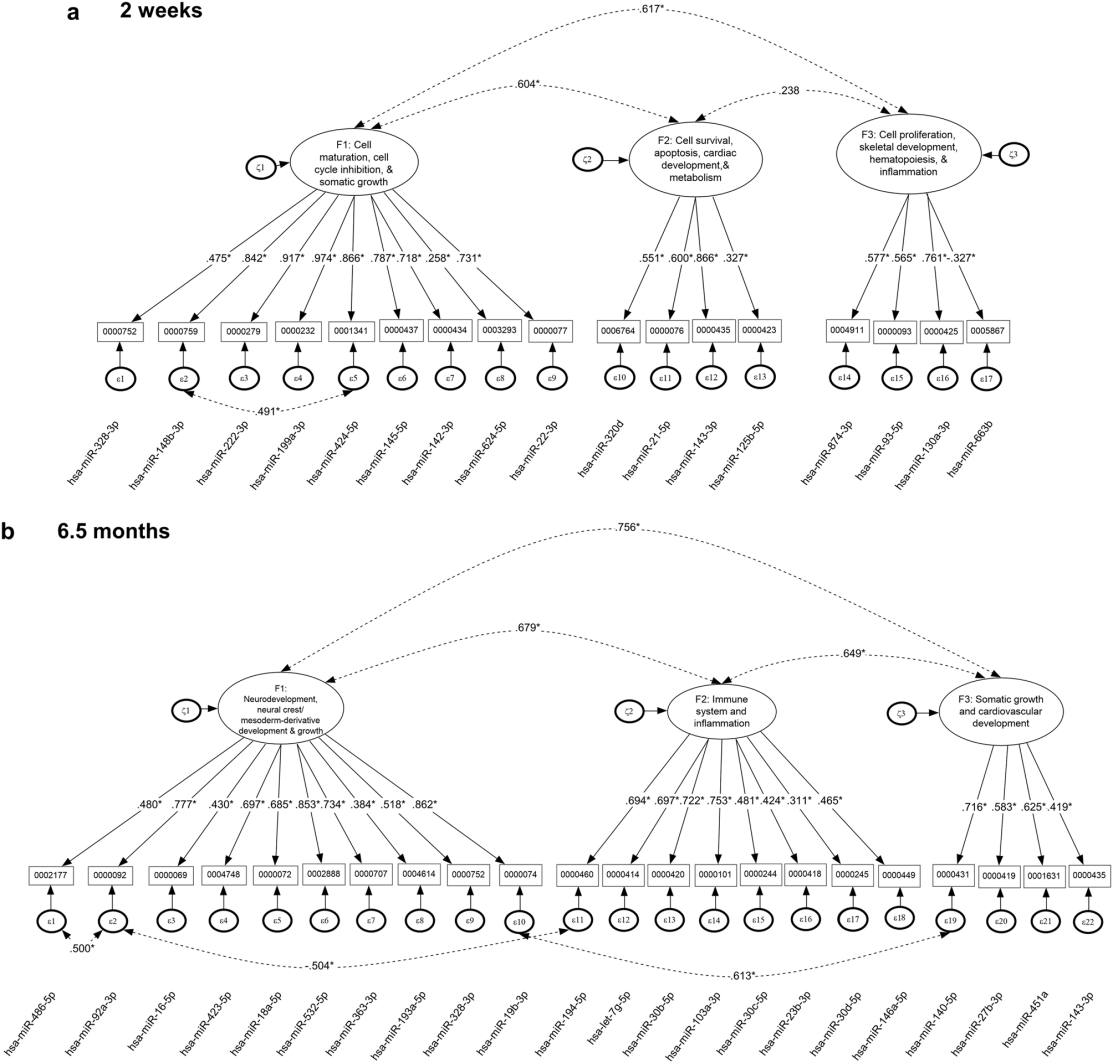
Confirmatory factor analysis clustered PAE-sensitive _*ex*_miRNAs into factors associated with distinct developmental pathways. Structural equation modeling of _*ex*_miRNA loading into the 3-factor solutions at (**a**) T_2wk_ and (**b**) T_6.5mo_. Factors are named based on IPA analysis of the canonical pathways and disease and biofunctions for the mRNAs targeted by the miRNAs within each factor (see Supplementary Fig. 6, 8). Figures were constructed using Inspiration (version 9.2.4).

At T_2wk_, IPA showed that the first factor included miRNAs related to cell maturation, cell cycle inhibition, and somatic growth, the second factor included miRNAs related to cell survival, apoptosis, cardiac development, and metabolism, and the third factor included miRNAs related to cell proliferation, skeletal development, hematopoiesis, and inflammation (Fig. 6a; Supplementary Fig. S5). This model provided an excellent fit to the data despite having a significant omnibus chi-square test, suggesting minimal discrepancies between the current model and “perfect” model fit (i.e., with zero residuals) [χ^2^(115) = 149.170, *p* = 0.018]. Fit indices were also excellent (Root Mean Square Error of Approximation (RMSEA) = 0.073, 95% CI = 0.032–0.104; Bentler’s Comparative Fit Index (CFI) = 0.920, Tucker-Lewis Index (TLI) = 0.906) prior to Bartlett’s correction. After applying the correction to the omnibus chi-square statistic, it became non-significant (χ^2^(115) = 127.434, *p* = 0.202), and two of the fit indices also improved after application of the correction (RMSEA = 0.43, 95% CI = 0.001–0.087; CFI = 0.971, TLI = 0.966) (see Supplementary Fig. S6a,b). No alterations from the hypothesized model were implemented, except for a residual covariation between miRNA MIMAT0001341 (miR-424-5p) and MIMAT0000759 (miR-148b-3p), and all items loaded significantly on their respective factors. The between-factors correlations ranged between 0.238 and 0.617 and were significant only between F1 and F2 and F1 and F3 with *p* < 0.001, suggesting correlated but distinct dimensions. This 3-factor structure at the neonatal period was compared to a unidimensional structure and to a bifactor model positing generalized and specific effects of the miRNAs in that both domain specific factors and a generalized factor would explain the relations between miRNAs (for a discussion on bifactor models see^54^). Results as mentioned earlier favored the 3-factor correlated solution using the information criteria (i.e., the BIC) and interpretational quality of the solution (Table 3). Internal consistency estimates using omega and maximal reliability *H* were 0.867 and 0.971 for the first factor, 0.553 and 0.804 for the second factor, and 0.354 and 0.711 for the third factor, respectively, indicating minimal amounts of measurement error and accurate measurement of the latent variables.

At T_6.5mo_, a 3-factor correlated solution provided the best model (Table 3). IPA showed that the first factor included miRNAs related to neurodevelopment, neural crest/mesoderm-derivative development and growth; the second, miRNAs related to immune system and inflammation; the third, miRNAs related to somatic growth and cardiovascular development (Fig. 6b, Supplementary Fig. S7). The measurement model showed good model fit, as all factor loadings were significantly different from zero. Prior to Bartlett’s correction, unstandardized residuals were 7.3%, fit indices were: CFI = 0.880, TLI = 0.864, and the chi-square test was significant [*χ*^2^(203) = 274.617, *p* = 0.001]. Post correction, model fit improved substantially with a point estimate of the residuals of 4.7% and a 95% CI ranging between 0.1 and 7.3%. The chi-square test was no longer significantly different from zero [*χ*^2^(203) = 233.253, *p* = 0.072] (Supplementary Fig. 6c,d) supporting an inference that the tested model does not deviate from a “perfect” model. The between factor correlations ranged from 0.649 and 0.756, indicating the absence of collinearity but the presence of three different yet highly correlated constellations of miRNAs. All between factor correlations were significantly different from zero and indicative of large effect sizes (*r*s > 0.50^55^). This 3-factor solution had three residual covariations between miRNAs, namely between miRNA MIMAT0000092 (hsa-miR-92a-3p) on Factor 1 and both MIMAT0002177 (hsa-miR-486-5p, Factor 1) and MIMAT0000460 (hsa-miR-194-5p, Factor 2) and between miRNAs MIMAT0000074 (hsa-miR-19b-3p) on Factor 1 and MIMAT0000431 (hsa-miR-140-5p) on Factor 3, suggesting the presence of a third variable that likely accounts for part of the variance not attributable to the latent constructs measured by Factors 1 and 2. Internal consistency reliability indicated low levels of measurement error. The omega coefficient estimates for the 3 factors were 0.807, 0.802, and 0.625, respectively. The estimates using maximal reliability were 0.918, 0.838, and 0.638, all within acceptable standards, although Factor 3 was lower than the others.

### Mediation of effects of PAE on somatic growth by _*ex*_miRNAs

Table 4 presents the results of the analyses examining the degree to which the effects of PAE (alcohol exposure (in AA/day), saturated linear regression) on the anthropometric measures are mediated by the expression levels of the _*ex*_miRNAs loading on each of the factors. The negative direct paths indicate that higher levels of PAE were associated with shorter infant length, lower weight, and smaller head circumference, after accounting for (a) the mediating role of the miRNA factors, and (b) the relation between alcohol exposure and smoking. The rows labeled “Indirect Paths” present the data from the mediation models for each of the factors. The indirect path rows assess the effect of the pathways from PAE to _*ex*_miRNA expression level and from _*ex*_miRNA expression level to the outcome. The rows labeled “Specific Indirect” show the degree to which the effect of PAE was mediated by the expression levels of the _*ex*_miRNAs in each factor.

**Table 4:** Models assessing mediation by _*ex*_miRNAs of the effects of prenatal alcohol exposure on postnatal infant growth, controlling for smoking.

Model Description	Mediating Factor					
	2-week _*ex*_miRNAs F1	2-week _*ex*_miRNAs F2	2-week _*ex*_miRNAs F3	6.5-month _*ex*_miRNAs F1	6.5-month _*ex*_miRNAs F2	6.5-month _*ex*_miRNAs F3
Direct Path: Length						
Exposure → Length	− 0.388*	− 0.388*	− 0.388*	− 0.414***	− 0.414***	− 0.414***
Mediational Paths						
Exposure → miRNAs	0.186**	− 0.400***	0.417***	− 0.313**	− 0.354***	− 0.422***
miRNAs → Length	− 0.407*	− 0.026	0.323^†^	0.166	− 0.341**	0.062
Specific Indirect	− 0.076^†^	0.01	0.135	− 0.052	0.121*	− 0.026
Direct Path: Weight						
Exposure → Weight	− 0.726***	− 0.726***	− 0.726***	− 0.523***	− 0.523***	− 0.523***
Mediational Paths						
Exposure → miRNAs	0.186*	− 0.397***	0.426***	− 0.313**	− 0.355***	− 0.422***
miRNAs → Weight	− 0.434*	− 0.06	0.659**	0.002	− 0.056	0.05
Specific Indirect	− 0.081	0.024	0.281*	− 0.001	0.02	− 0.021
Direct Path: HC						
Exposure → HC	− 0.542***	− 0.542***	− 0.542***	− 0.550***	− 0.550***	− 0.550***
Mediational Paths						
Exposure → miRNAs	0.187**	− 0.400***	0.424***	− 0.314***	− 0.354***	− 0.420***
miRNAs → HC	− 0.24	0.028	0.363*	0.182	− 0.128	− 0.284
Specific Indirect	− 0.026	− 0.016	0.154*	− 0.057	0.045	0.119

Mediational models predicting growth from alcohol exposure (direct effect) and through miRNAs (mediating variables). The effects of alcohol exposure on growth are adjusted for level of maternal smoking during pregnancy. Estimates are standardized regression coefficients for the direct paths and for each of the indirect paths. Direct path = effect of PAE on the growth measure; mediational paths = effects of PAE on _*ex*_miRNA expression level and effects of _*ex*_miRNA expression level on the growth measure; specific indirect = the degree to which the effect of PAE was mediated by the expression levels of the _*ex*_miRNAs in each factor. Estimates for the specific indirect paths are the product of the regression coefficients for the two indirect paths. HC = head circumference; Exposure = prenatal alcohol exposure (AA/day); 2-week _*ex*_miRNAs F1 = cell maturation, cell cycle inhibition, and somatic growth; 2-week _*ex*_miRNAs F2 = cell survival, apoptosis, cardiac development, and metabolism; 2-week _*ex*_miRNAs F3 = cell proliferation, skeletal development, hematopoiesis, and inflammation; 6.5-Month _*ex*_miRNAs F1 = neurodevelopment, neural crest/mesoderm-derivative development, and growth; 6.5-Month _*ex*_miRNAs F2 = immune system and inflammation; 6.5-Month _*ex*_miRNAs F3 = somatic growth and cardiovascular development; ^†^*p* < 0.10; *p < 0.05; **p < 0.01; ***p < 0.001.

At T_2wk_, all direct effects were negative and significant, showing that higher levels of exposure were associated with decrements in all three growth parameters. Because higher _*ex*_miRNA values (increased CT) indicate lower levels of expression, among the indirect paths PAE was associated with decreased expression of _*ex*_miRNAs associated with (F1) cell maturation, cell cycle inhibition, and somatic growth and (F3) cell proliferation, skeletal development, hematopoiesis, and inflammation and with elevated miRNA expression of _*ex*_miRNAs associated with (F2) cell proliferation, skeletal development, hematopoiesis, and inflammation in relation to all three anthropometric measures. Partial mediation, in which both the direct path and specific indirect path were significant, was observed for weight and head circumference by _*ex*_miRNAs associated with (F3) cell proliferation, skeletal development, hematopoiesis, and inflammation.

As at T_2wk_, all direct effects at T_6.5mo_ were negative and significant, showing that higher levels of exposure were associated with greater decrements in all three growth parameters. Among the indirect paths, higher PAE was associated with elevated miRNA exposure for all three domains, namely, (F1) neurodevelopment, neural crest/mesoderm-derivative development and growth, (F2) immune system and inflammation, and (F3) somatic growth and cardiovascular development. (F2) immune system and inflammation _*ex*_miRNAs partially mediated the effect of PAE on length.

### _*ex*_miRNAs mediate the effect of PAE on cognitive functioning

**Figure 7:**
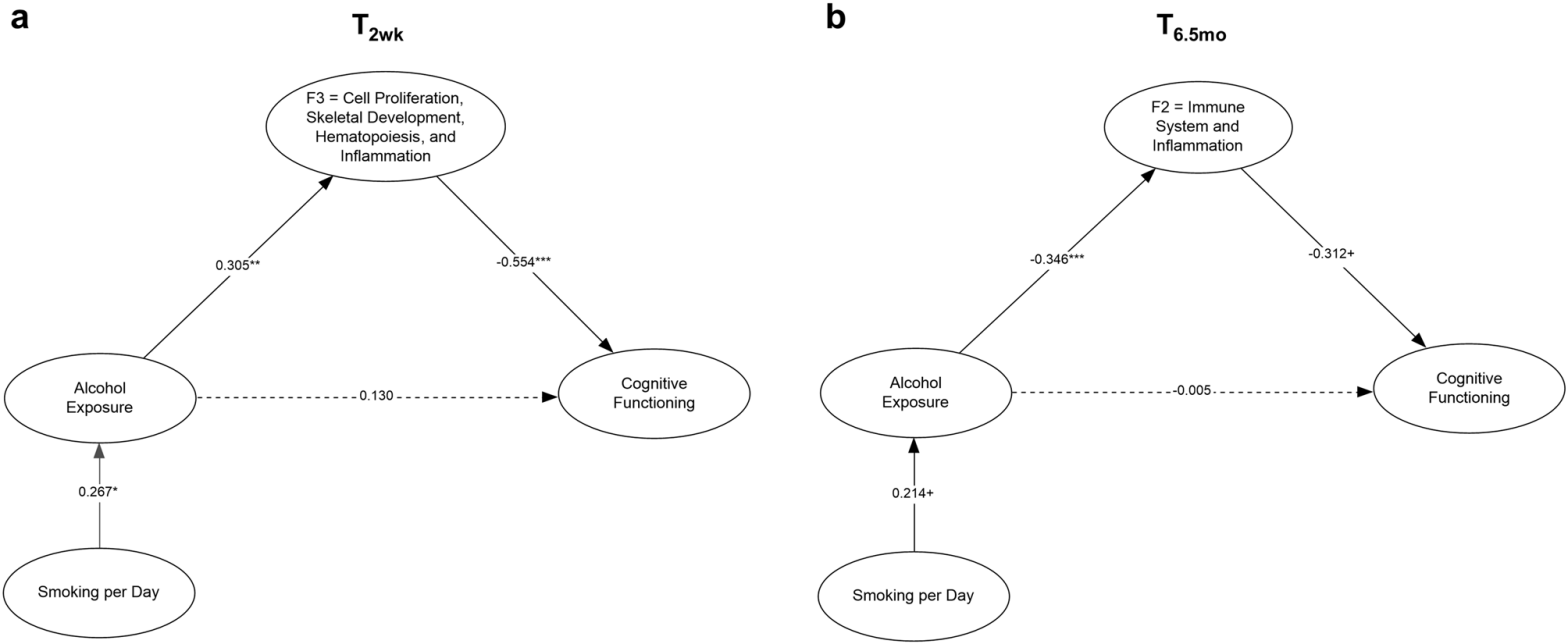
Mediational model predicting cognitive function. Cognitive function (measured by FTII visual recognition memory at 6.5 months) mediation from the direct effect of alcohol exposure and the indirect effect of alcohol exposure mediated by the _*ex*_miRNA factors at (**a**) T_2wk_ and (**b**) T_6.5mo_ and adjusted for maternal smoking during pregnancy. Model included all three miRNA factors with residual correlations between them^139^. For T_2wk_, figure displays only F3, for which significant mediation was found. The indirect effect of alcohol exposure on cognitive functioning through F3 was significant (*b* = − 0.169, *p* = 0.032). The factors for which significant mediation was not evident (F1 and F2) are omitted from the figure for clarity. For T_6.5mo_, figure displays only F2, for which the mediational effect fell short of conventional levels of statistical significance (*b* = 0.108, *p* = 0.109), but using a one-tailed test mediation is evident. The factors for which significant mediation was not evident (F1 and F3) are omitted from the figure for clarity. ^†^*p* < 0.10; *p < 0.05; **p < 0.01; ***p < 0.001. ). Figures were constructed using Inspiration (version 9.2.4).

Mediation of the effect of alcohol exposure on the Fagan Test of Infant Intelligence (FTII;^56^) visual recognition memory was significant for one _*ex*_miRNA factor: T_2wk_ (F3) cell proliferation, skeletal development, hematopoiesis, and inflammation (Fig. 7a). The indirect effect of alcohol exposure on cognitive functioning through F3 was significant (*b* = − 0.169, *p* = 0.032), and the model fit the data well [χ^2^(4) = 3.355, *p* = 0.500; CFI = 1.00; TLI = 1.00]. A similar model also fit well at T_6.5mo_, [χ^2^(4) = 9.001, *p* = 0.061; CFI = 0.965; TLI = 0.869]. As shown in Fig. 7b, the data suggest partial mediation at T_6.5mo_ of the effect of PAE on cognitive functioning by (F2), immune system and inflammation _*ex*_miRNAs (*b* = 0.108, *p* = 0.109).

## Discussion

PAE is associated with a broad range of cognitive deficits, including lower IQ^57, 58^, poor attention and executive function^59–62^, deficits in eyeblink conditioning^24, 63–66^ and number processing^67, 68^, and slower cognitive processing speed^57, 67, 69–72^. Although prenatal alcohol effects are irreversible, early interventions and remediation may mitigate severity of intellectual and behavioral impairment^73^. However, identification of affected individuals in need of early intervention is challenging both because FASD diagnostic clinical assessment is not universally available and because most PAE-affected individuals may be nonsyndromal and, therefore, lack the characteristic pattern of craniofacial dysmorphic features and growth deficits seen in FAS. There is thus a clear and pressing need for supplemental diagnostic approaches that can be more widely deployed as a screening tool for FASD in the general population. We and others recently found that plasma miRNAs in the pregnant mother could be a potential index not only for exposure^29^ but also for predicting which infants were more likely to meet criteria for a diagnosis of FAS or PFAS^28^. In the current study, based on our preclinical data in an ovine model^27^, we hypothesized firstly that PAE would influence the pattern of plasma miRNAs in the newborn infant, and secondly, that these infant _*ex*_miRNAs would predict infant growth and cognitive outcomes, thus enabling better early identification of PAE-affected infants. Findings from this study lend support to these hypotheses.

PAE was associated with alterations in patterns of _*ex*_miRNA expression at both 2 weeks and 6.5 months, demonstrating the utility of _*ex*_miRNAs as biomarkers of exposure at different ages. PAE-related _*ex*_miRNAs were different at the two ages, though there was a clear bias towards increased miRNAs in infant plasma due to PAE such that by 6.5 months almost all of the altered _*ex*_miRNAs were elevated in PAE infants relative to controls. Likewise, the number of likely sex-specific _*ex*_miRNAs also increased over development, with 3.5-fold more at T_6.5mo_ than T_2wk_. Four of the 18 _*ex*_miRNAs altered to a clinically-relevant level at T_2wk_ were also altered at T_6.5mo_ (Supplementary Table S8), and three of these four were altered in the same direction and with a greater effect size at T_6.5mo_ (*d* increased by 0.07–0.12). In addition, some of the miRNAs identified in this study have also been found in our previous work identifying miRNA biomarkers in pregnant women^28^ and in an ovine model^27^. T_2wk *ex*_miRNAs had the best concordance with the infant-outcome predictive model from our previously published maternal data^28^, while at T_6.5mo_ the best concordance is from the lamb neonate^27^. These findings suggest a transition away from maternally-programmed miRNA alterations at 2 months to an adaptive growth and development driven profile at 6.5 months.

At T_2wk_, changes in _*ex*_miRNAs in the PAE group may be attributable to the direct effect of PAE on fetal organ and tissue development, whereas the differences in _*ex*_miRNA expression seen at T_6.5mo_ may be due to the downstream effects of these in utero insults later in development and/or facilitate compensatory adaptations to the effects of PAE. For example, secreted miR-126-3p (MIMAT0000445) has been shown to promote angiogenesis^74^, which has, in turn, been shown, in pre-clinical models, to be inhibited by PAE^75^, whereas secreted miR-146a (MIMAT0000449) has been shown to suppress inflammation^76, 77^, which is induced in PAE models^78^. Further research using preclinical models will be necessary to determine the biological roles of these identified PAE-sensitive _*ex*_miRNAs, particularly as _*ex*_miRNAs expressed in concert can have effects that are different than a summation of the individual effects of each miRNA^79^. For instance, we previously identified _*ex*_miRNAs significantly elevated in maternal plasma from heavy alcohol exposed pregnancies^28^ and preclinically and found that these _*ex*_miRNAs together, but not individually, inhibited placental growth and maturation^79^.

In the newborn period, altered levels of _*ex*_miRNAs in the PAE group showed higher intercorrelated expression than was seen in the control group. The greater intercorrelated expression of _*ex*_miRNAs transcribed from specific chromosomes at T_2wk_ suggests substantial chromatin reorganization in contributory cells and the emergence of new nuclear transcription factories as a consequence of PAE. Such factories, containing loops of chromatin from different chromosomes, are thought to be the functional unit within cellular nuclei for co-regulated transcription, including the regulation of multiple gene loci by a single transcription factor^51^, to adapt and reorganize to influence cellular differentiation states^80^. Recently, pro-inflammatory cytokines, such as tumor necrosis factor (TNF)-α, which are reportedly elevated in PAE mice following nerve damage^81^, have also been shown to induce the reorganization of transcription factories to promote transcription at miRNA-encoding genes^82^. It is also possible that a reactive network of cytokines coordinates _*ex*_miRNA secretion from multiple tissues, following PAE. Co-secretion of _*ex*_miRNAs from muscle, endothelial cells, and other tissues has been hypothesized to occur following muscle damage^83^ and may coordinate damage repair mechanisms that rely upon a number of tissues^84^. The much smaller number of PAE-related chromosomal intercorrelations observed at T_6.5mo_ suggests that some direct effects of PAE on chromatin reorganization in these regions may be transient or altered by the effects of the early postnatal environment.

Mediation models indicated that at T_2wk *ex*_miRNAs associated with cell proliferation, skeletal development, hematopoiesis, and inflammation (F3) mediated the effects of PAE on weight and head circumference, after controlling for maternal smoking during pregnancy. At T_6.5mo_, _*ex*_miRNAs associated with immune system and inflammation (F2) mediated the effects of PAE on length, after controlling for maternal smoking during pregnancy. The majority (> 70%) of _*ex*_miRNAs within these factors did not exhibit sex differences (Supplementary Table S9), indicating that the mediation of the effects of PAE by these _*ex*_miRNAs likely occur in a sex-independent manner.

The apparent relevance of hematopoiesis-related _*ex*_miRNAs to PAE-related growth restriction is consistent with our prior work demonstrating that PAE was related to a 70% increase in the prevalence of iron deficiency anemia at age 6.5 months and that fetal alcohol-related growth restriction was markedly more severe among infants with iron deficiency anemia^85, 86^. In addition, in a longitudinal study we recently reported that effects of PAE on IQ, learning and memory, and cognitive flexibility in childhood were stronger in children with PAE-related postnatal growth restriction^87^. Studies in preclinical animal models have shown similar interaction effects between iron status, anemia, and PAE-induced growth and neurobehavioral deficits^88^. Intriguingly, this hematopoeisis related factor (T_2wk_ F3) also partially mediated the effects of PAE on FTII infant visual recognition memory. FTII performance, which assesses basic information-processing skills involving encoding and retrieval, has been repeatedly shown to predict intellectual function in childhood^89^, whereas the more frequently used global Bayley Scales of Infant Development rely heavily on sensorimotor manipulation of objects and have poor predictive validity^90^. Thus, the observed mediation of effects on growth suggests that _*ex*_miRNAs during the newborn period may provide early biomarkers of vulnerability to long-term PAE-related intellectual impairment.

This study thus provides the first evidence that levels of _*ex*_miRNAs during early infancy can provide a biomarker of the effect of PAE on a measure of cognitive processing abilities in infancy that is predictive of school-age IQ. PAE-altered _*ex*_miRNAs found to play functional mediating roles in FASD may be targets for therapeutic intervention, using strategies such as antagomiRs and synthetic miRNAs as have been seen in clinical trials for therapies for cancer and hepatitis C^91^.

The differences in the _*ex*_miRNA patterns attributable to PAE at T_2wk_ and T_6.5mo_ suggest that these patterns evolve with postnatal development and may be modified by adverse postnatal environmental influences, such as maternal stress^92, 93^ or the transgenerational effects of early life stress^93–95^. A more detailed study of the stability of infant and child _*ex*_miRNA expression patterns will be needed to tease apart the relative contributions of PAE, development, and the postnatal environment. The generalizability of these _*ex*_miRNAs as biomarkers of PAE requires further validation studies to assess the robustness of these identified _*ex*_miRNAs, particularly as the best practices in _*ex*_miRNA analysis continue to evolve^96^, the duration of their expression, and preclinical studies to assess whether mediating _*ex*_miRNAs directly contribute to the PAE growth and neurodevelopmental deficits, as has seen with maternal _*ex*_miRNAs^79^, or are byproducts of the biological mechanisms underlying these deficits. The biological function of these _*ex*_miRNAs in the factors were determined based on our current understanding of gene function and miRNA targeting, and as our understanding evolves, additional biological functions may attributable to these factors. In addition, although our study was not specifically powered to address the impact of infant sex on _*ex*_miRNA expression profiles, bootstrap resampling analyses indicated more pronounced sex-segregated PAE-related differences in _*ex*_miRNA expression levels at T_6.5mo_ than at T_2wk_. (Fig. 3). Follow-up studies specifically powered to ascertain the effect of infant sex on _*ex*_miRNAs following PAE are, therefore, warranted.

This study has additional limitations common to other longitudinal studies of PAE. Misclassification of some cases with regard to PAE may obscure some associations, but we have previously validated the timeline follow-back alcohol ascertainment protocol used in this study in relation to levels of fatty acid ethyl esters metabolites in meconium samples in this community^21^ and to infant and child behavior^17, 24, 97^, somatic growth^87^, and brain structure^98–100^ and function^101^. Some unmeasured confounders may have played a role in these findings, such as differences in unmeasured environmental exposures, such as household smoking, or lead or pesticide exposure. However, both the exposed and control groups were recruited from the same community, and socioeconomic status^42^ did not differ between groups, nor did weight gain during pregnancy or nutritional status across most micronutrients^102^.

## Conclusions

In summary, our findings suggest that alterations in _*ex*_miRNAs in infant plasma have the potential to serve as biomarkers of both PAE and of the effects PAE on growth and cognition. Biomarkers of the developmental effects of alcohol may facilitate detection of affected infants who do not display overt physical characteristics of PAE and referral of infants with FASD for interventions during early sensitive periods of development. These findings thus respond to the consensus statement issued by the American Academy of Pediatrics^40^ and the Interagency Coordinating Committee on Fetal Alcohol Spectrum Disorders highlighting the urgent need for new tools to aid in the identification and ultimately, the diagnosis of FASD. These findings build on our previous report that maternal _*ex*_miRNAs can predict FAS and PFAS to now show that infant _*ex*_miRNAs can provide direct indications of fetal damage. This study is the first to test directly whether _*ex*_miRNAs that discriminate between alcohol-exposed and non-exposed infants mediate effects of PAE on specific outcome domains, namely, growth and cognition. In summary, this study indicates the potential of miRNAs as biomarkers for predicting PAE-related developmental impairment and perhaps other developmental origins of health and disease.

Further validation studies in different infant populations will be needed to determine if _*ex*_miRNAs are a robust biomarker of effect.

## Methods

### Participants

The sample consisted of 68 Cape Coloured (mixed ancestry) mothers and their infants (37 heavy alcohol exposed, 31 healthy controls) from a larger prospective cohort^87, 103^. The Cape Coloured population historically comprised the large majority of workers in the wine-producing region of the Western Cape. The high prevalence of FAS in this community is a consequence of very heavy maternal drinking during pregnancy, due to poor psychosocial circumstances and that farm laborers were historically paid, in part, with wine^104^. Drinking, which is concentrated primarily on 2–3 days on the weekends^24^, continues to be a major source of recreation for many in urban and rural Cape Coloured communities^9^, despite numerous public health interventions to reduce pregnancy drinking.

The infants in this study were born to women who were recruited between 2013 and 2015, at their first antenatal visit, at one of two midwife obstetrical clinics that serve economically disadvantaged areas of Cape Town. Each mother was interviewed antenatally regarding her alcohol use using the ‘gold standard’ 2-week timeline follow-back interview^17^, adapted to reflect how pregnant women in this community drink. The interview included information about type and amount of each beverage consumed at time of conception and across pregnancy, as well as about container size (including pictures of different containers, bottles, cans, glass size) and sharing (by how many individuals), for use in calculation of amount of alcohol consumed. At recruitment, the mother was interviewed regarding incidence and amount of drinking on a day-by-day basis during a typical 2-week period at time of conception. Volume was recorded for each type of beverage consumed each day and converted to oz absolute alcohol (AA), using weights that reflect potency of AA in Cape Town (liquor**—**0.4, beer**—**0.05, wine**—**0.12, cider**—**0.06). The woman was then asked whether her drinking had changed since conception; if so, when the change occurred and how much she drank on a day-by-day basis during the past 2 weeks. Maternal exclusionary criteria were age < 18 years, HIV infection, multiple gestation pregnancy, and pharmacologic treatment for chronic medical conditions, including diabetes, hypertension, epilepsy, or cardiac problems.

Two groups of women were recruited: heavy drinkers, who consumed 14 or more standard drinks/week (1.0 oz AA/day, equivalent to 28 g or 30 ml AA/day) and/or engaged in binge drinking (4 or more drinks/occasion), and controls, who abstained or drank only minimally during pregnancy (83.9% abstained, 12.9% drank 1–2.5 drinks on 1–2 occasions, one consumed 3 drinks on 2 occasions around conception). The 2-week timeline follow-back interview was repeated at 4 and 12 weeks after recruitment. Data from the three alcohol consumption interviews were averaged to provide continuous measures of drinking across pregnancy: average oz AA consumed/day, AA/drinking day (dose/occasion), and frequency (days/week). Mothers were also asked about their drug use during pregnancy. Marijuana (“dagga”), cocaine, heroin, methaqualone (“mandrax”), and methamphetamine (“tik”) were measured in days/month; smoking, as cigarettes/day. All women who reported drinking during pregnancy were advised to stop or reduce their intake and were offered referrals for treatment, if requested. Infant exclusionary criteria were major chromosomal anomalies, neural tube defects, multiple births, very low birth weight (< 1500 g), GA < 30 weeks, and seizures.

Human subjects’ approval was obtained from the Wayne State University, the University of Cape Town Faculty of Health Sciences, and Texas A&M University Institutional Review Boards. All mothers provided written informed consent. All procedures were followed according to the relevant guidelines.

### MiRNA expression analysis

#### Sample collection

Venous EDTA-stabilized plasma samples were obtained from the infants at 2 weeks (T_2wk_) and 6.5 months (T_6.5mo_) postpartum by a trained phlebotomist, placed on ice, and centrifuged and decanted immediately to avoid contamination with intracellular miRNA. Approximately 1 ml of whole blood was drawn into an EDTA tube to ensure at least 500 µL plasma. The samples were stored at − 80** °C** and subsequently shipped on dry ice to RCM’s laboratory for _*ex*_miRNA analyses (see below). Samples were obtained from 68 infants in total; 58 infants were sampled at both T_2wk_ and T_6.5mo_ and an additional 10 infants at T_6.5mo_ only.

#### Sample preparation and quality control analysis

Total plasma RNA, including lipoprotein and protein bound and extracellular vesicle packaged, was isolated from 80 to 200 μl plasma using the miRNeasy Mini RNA isolation kit (Qiagen, Gaithersburg, MD) with 1.2 µg MS2 carrier RNA added per 200 µL of plasma (Roche Diagnostics, Indianapolis, IN). RNA concentration was determined using a NanoDrop ND-1000 spectrophotometer, and RNA samples were stored at − 80 °C prior to use.

Plasma miRNA content can be contaminated by miRNAs from lysed erythrocytes^105^. We controlled for possible erythrocyte contamination in three steps, as described elsewhere^27, 28^ (see Supplementary Fig. S1). First, the presence of free hemoglobin was assessed in the plasma samples, using absorbance at 414 nm which has been shown to be an indicator of hemolysis^105^ and has been used previously by our laboratory to assess plasma purity. Each sample was then assessed both for the presence of mRNA for the erythrocyte-specific band-3 membrane protein (SLC4A1) and for levels of erythrocyte-enriched miR-451a (MIMAT0001631) relative to miR-23a-3p (MIMAT0000078). SLC4A1 content was assessed with quantitative RT-PCR after qScript cDNA Synthesis Kit (Quantabio, Beverly, MA) using PerfeCTa SYBR Green FastMix (Quantabio) with gene-specific primers (forward: 5′-aacgagtgggaacgtagctg-3′; reverse: 5′-cttcatattcctcctgctccag-3′). RNA isolated from sheep red blood cells was used as a positive control. Three samples had 414 nm absorbance and miR-451a enrichment (ΔCT_(miR-23a-3p -miR-451a)_) over hemolysis-indicator thresholds (> 0.3 and > 7, respectively)^43^ and were excluded from subsequent miRNA expression analysis (one sample each for Control T_2wk_, PAE T_2wk_, and PAE T_6.5mo_). Therefore, miRNA data is derived from 56 samples T_2wk_ and 67 samples at T_6.5mo_.

To examine the possibility that the RNA collection method contained inhibitors for cDNA synthesis, cDNA was synthesized from two independant RNA samples that were spiked with cel-miR-39-3p and UniSp6 RNA, according to the miRCURY LNA RNA Spike-in Kit. UniSP6 was added at 100 × higher concentration than cel-miR-39-3p. The RNA was then added into 20 μL cDNA synthesis reactions at increasing amounts (5 ng, 10 ng, 20 ng, 40 ng). These cDNA were then diluted 1:100 into the qPCR mix and the quantity of the spike-ins was assessed using appropriate primers (Exiqon/Qiagen). These analyses showed no evidence for the presence of endogenous inhibitors of cDNA synthesis and PCR amplification (see Supplementary Fig S1).

#### miRNA analysis

Using 25 ng of RNA input, cDNA was synthesized using the miRCURY LNA Universal RT cDNA synthesis kit (Exiqon/Qiagen). A standardized input of cDNA (40 µl) was diluted 110 × and then combined 1:1 with the ExiLENT SYBR green master mix (Exiqon/Qiagen). miRNA content was assessed using Human miRCURY LNA miRNA miRNome PCR Panels (V4; Exiqon/Qiagen). These arrays are contained in 2 X 384 well plates and assess 752 unique miRNAs. Quantitative PCR was then performed using Applied Biosystems 7900HT Fast Real-Time PCR System (Applied Biosystems/Thermo Fisher Scientific, Waltham, MA). Amplification data were visually inspected to ensure proper amplification of target miRNA. Interplate controls were assessed to determine equal performance of each panel. At T_2wk_, 8 panel I plates did not reach amplification criteria and the miRNAs from panel I, but not panel II, were excluded for these samples. CTs for each amplicon were determined using SDS2.4 software (Applied Biosystems/Thermo Fisher Scientific).

### Infant assessments

Mothers and infants were transported in a research van by our research driver and nurse to the Cape Universities Brain Imaging Center (CUBIC) for scanning and to our University of Cape Town Mother–Child Research Development Laboratory at 6.5 months postpartum for cognitive assessment. Weight, length, and head circumference were measured by our research assistants, who were trained by RCC, using standard WHO protocols^106^ at 2 weeks and 6.5 months (see^87^). Length-for-age, weight-for-age, and head circumference-for-age *z*-scores were calculated from measurements obtained at 2 weeks, 6.5 months, and at a diagnostic clinic in 2016 (see below). GA was based on early gestation ultrasound, which was available for 92.6% of the study participants, or date of last menstrual period.

#### Fagan test of infant intelligence

Visual recognition memory was assessed on the FTII^107^, which was administered by Master’s-level psychologists at the 6.5-month visit. The infant, seated on the mother’s lap, is shown identical photos of a human face and then a novel photo of another face paired with the familiar one. The normative response, preference for the novel stimulus, indicates the ability to recall the familiar stimulus and discriminate it from the novel one. The infant is administered 10 visual comparison problems consisting of pairs of faces. Infant fixation is recorded on a computer, and preference for novelty is computed by dividing duration of time looking at the novel stimulus by total time looking at the paired familiar and novel stimuli for each of the 10 problems. We have previously reported that the FTII is sensitive to PAE and that the specific effects of PAE on FTII are not seen in relation to other exposures, including smoking, cocaine, or marijuana^72, 108^.

#### FASD diagnosis

In 2016, we organized a clinic in which the infants, including the 68 in the present study (mean age = 1.7 yr, SD = 0.6; range = 0.9 to 3.1 yr), were examined for growth and FAS anomalies using a standard protocol^10^. Each child was independently examined by HE Hoyme (HEH), MD, an expert FASD dysmorphologist, and a second examiner trained by HEH (G De Jong, MD; H Bezuidenhout, MD; E Krzesinski, MD; RCC, MD). HEH, the other dysmorphologists, and SWJ, JLJ, RCC, and CDM, subsequently conducted case conferences to reach consensus regarding which infants met criteria for diagnoses of FAS or PFAS. Diagnosis was based on the 2005 Revised Institute of Medicine Guidelines^10^. FAS is characterized by microcephaly, growth retardation, and at least two of the distinctive craniofacial anomalies linked to fetal alcohol exposure: short palpebral fissures, flat philtrum, thin vermilion (upper lip). PFAS was diagnosed when at least two of these facial characteristics were present in conjunction with microcephaly or growth retardation and there was confirmed evidence of maternal drinking during pregnancy. Heavily exposed infants who did not meet criteria for FAS or PFAS were classified as nonsyndromal HE.

### Statistical analyses

#### Group difference and effect size estimates

CTs were computed for each expressed miRNA (_expressed_miRNA_CT). The average CT of all expressed miRNAs in each sample was computed (_average_CT), and the levels of each miRNA in each sample were expressed as ΔCT (_expressed_miRNA_CT—_average_CT). ΔΔCT, ΔCT_PAE_-ΔCT_Control_, is reported for comparison between groups, with negative values indicating higher expression in the PAE group and positive values indicating higher expression in the control group. For the 148 miRNAs whose expression was detected in at least 80% of the samples in at least one group at one age, miRNAs that were below the level of detection for the assay were assigned a CT value of 1 greater than the highest value of the given miRNA that was detected, in accordance with research that shows including these non-detected reactions as data points reduces bias^109^ and that best practices to replace these non-detected values with an experimentally relevant value^96, 110^. A low ΔCT value indicates higher level of miRNA expression, and as with CTs, a unit change in ΔCT, or ΔΔCT, of 1.0 indicates a twofold difference in miRNA expression.

Given the prevalence of smoking within this population (Table 1), we used an ANCOVA model to test for exposure group differences in mean expression of each miRNA, adjusted for average number of cigarettes smoked during pregnancy per day. These analyses were performed on the 148 miRNAs whose expression was detected in at least 80% of the samples in at least one group at one age. Given the relatively small sample size in relation to the number of tests run, Cohen’s *d*^55, 111^ effect size estimates of ANCOVA-adjusted mean expression were used to identify meaningful between-group differences. In Cohen’s *d*, which compares standard deviation differences between two groups, differences of 0.20, 0.50, and 0.80 are considered small, medium, and large effects, respectively. In this study we used a small-to-medium effect size cut-off of 0.40 to identify miRNAs on which the exposed and control groups differed. This criterion was selected because (a) 0.40 is considered the lower bound for a clinically meaningful effect size^112^; (b) an effect size of 0.40 corresponds to an odds ratio of 2.0, indicating twice the odds that the outcome will be observed in the exposed than in the control group^113^; (c) with an effect size of 0.40, based on Cohen’s^111^ U3 statistic, 66% of the participants in the exposed group will be above the mean for the participants in the control group; (d) using Rosenthal and Rubin’s^114^ binomial effect size display (BESD), an effect size of 0.40 indicates a 20% between-group difference on the outcome of interest. A large proportion of the effect sizes for the differences in miRNA expression identified using this approach was substantially larger than 0.40 (see Table 2, Supplementary Table S2). While Hedge’s *g* has been shown to have decreased bias for small sample sizes ^115^, our sample is sufficiently large that Cohen’s *d* and Hedges’s *g* vary by < 0.1% (Supplementary Table S2). When examining the population segregated by sex, in which the sample size for each group is smaller, the more conservative Hedges’s *g* effect size estimate is reported (Table 2).

#### Within-group correlation analysis

To assess the extent to which PAE resulted in coordinated expression of _*ex*_miRNAs, Pearson’s correlations between the expression levels of ΔCT values in all possible pairs of the 148 miRNAs were generated. Correlation plots were created for the control and alcohol exposed groups separately for each time point using the “corrplot” package^116^ implemented in R^117^ (see Supplementary Methods S10). We calculated *p*-values for each correlation, and correlations with *p* ≥ 0.05 were masked. Correlation matrices were ordered using two approaches—first, by a complete hierarchical clustering method that aims to identify highly correlated clusters in the measured variables; then, by using the chromosomal location of the miRNAs (ftp://mirbase.org/pub/mirbase/20/genomes/hsa.gff3). The number of significant correlations was reported for each of the infant groups at each time point. To test the stability of the pairwise correlations with *p*-values less than 0.05, we bootstrapped the estimates of correlation coefficients using sampling with replacement (see Supplementary Methods S10). Additionally, we reported the correlations between miRNAs with effect sizes greater than 0.40.

#### Confirmatory factor analysis

The PAE-responsive miRNAs (i.e., those with a group difference of Cohen’s *d* ≥ 0.40) were examined using CFA. CFA uses a simultaneous equation approach to estimate discrepancies between a population Σ(Θ) and a sample’s variance–covariance matrix S(θ), using an iterative procedure. Model fit is assessed using an omnibus chi-square test and three descriptive fit indices, among which the most preferred are Bentler’s CFI^118^, the TLI^119^, and Steiger and Lind’s^120^ RMSEA. Values of good model fit are indicated by fit-index values greater than 0.95^121^ and residual values less than 0.10^122^ and 0.08 for the other two indices, respectively^123, 124^. In light of the relatively small sample size in the present study, we adjusted the omnibus chi-square test value, and consequently the values of all other indices, using Bartlett’s^125^ correction using an R-function we developed for that purpose, which accounts for sample size and model complexity as shown below:

1 $${\text{Bartlett}'\text{s}}\;{\text{Corrective}}\;{\text{Factor}} = 1 - \frac{{4{\text{k}} + 2{\text{p}} + 5}}{{6{\text{n}}}}$$

with *k* being the number of latent variables, *p* the number of observed variables and *n* the sample size + 1. CFAs were run separately to examine the miRNA expression levels seen at each of the two ages. The expression levels of the miRNAs loading on each of the factors at each age were averaged within groups to provide the mean expression level for that factor.

#### Power for the CFA models

A Monte Carlo simulation of the CFA model at the first time-point, T_2wk_, was run by generating population data to test the sample size required to find significant factor loadings of specified minimum values and overall model fit indices. The specified parameters were: (a) factor loadings = 0.50, (b) factor means and variances at 0 and 1, respectively, for identification, (c) factor covariance at 0.50, standardized value, and, (d) item residual variances = 0.75. A model with *n* = 59 was run using 10,000 replicated datasets, as recommended^126^. Results indicated that coverage ranged between 92.9 and 94.0%. Power for the significance of the factor loadings ranged between 89.5 and 92.9%. Power for the standardized covariance estimate was 90.7%. Out of the 10,000 samples, the analysis converged with 9,989. Percentage bias of the estimated parameters was set to a maximum of 10% and should ideally be less than 5%^126^. Bias was calculated by subtracting the population value from the average parameter estimate, dividing by the population value, and then multiplying by 100. Results indicated that the largest bias was at 1.8%, which is well below the lowest acceptable standard (i.e., 5%). The results for the 3-factor model at 6.5 months (T_6.5mo_) are not reported here because with the exact same configuration, the sample size of *n* = 67 would be associated with even higher power and more stable parameter estimates in the estimation of the factor loadings and the between factor correlations. These simulation findings generally agree with an earlier simulation study^127^, in which sample sizes with between 50 and 70 participants were associated with nominal Type-I errors (of the chi-square test), proper coverage (> 90%), and stable parameter estimates (e.g., of factor loadings, item variances, etc.).

#### Internal consistency reliability

In light of the limitations in the estimation of Cronbach’s alpha and its highly restrictive assumptions (e.g., requiring essential tau equivalence), two indices directly estimable from the CFA model were utilized; namely, the omega coefficient^128^ and maximal reliability H^129^. The omega index^128, 130, 131^ is similar to alpha but has the advantage of allowing for heterogeneous item-latent variable correlations, thus enabling accurate estimation of measurement errors at the item level with congeneric measures (i.e., non-tau-equivalent). It is estimated as follows:

2 $$Omega = \frac{{\left( {\sum \lambda_{i} } \right)^{2} }}{{\left( {\sum \lambda_{i} } \right)^{2} + \sum Var(\varepsilon_{i} )}}$$

with *λ*_*i*_ being the factor loadings of item *i* and *Σvar*, the respective error variances of item *i*. Omega was adjusted for collinearity in the residuals as recommended by Wang and Wang^132^ following a procedure detailed in the Supplementary Methods S10. Maximal reliability *H* was estimated using an optimally weighted composite comprised of standardized factor loadings, which provided the advantage of allowing negative factor loadings to contribute meaningful variance to the latent construct^133^. It was estimated as follows^134, 135^:

3 $$H = \frac{{\sum \frac{{l_{i}^{2} }}{{1 - l_{i}^{2} }}}}{{1 + \sum \frac{{l_{i}^{2} }}{{1 - l_{i}^{2} }}}}$$

with *l*^2^ being the standardized factor loading of item *i* squared^133^. The advantages of the maximal reliability coefficient compared to omega are that (a) negative factor loadings are modeled properly, (b) it uses a weighted estimate by squaring the individual factor loadings^133^, (c) the estimated reliability can never be less than reliability of the best measured item, and (d) the weighing procedure downgrades less informative items that load weakly on the factor^136^. Between the two estimates, more value will be placed on maximal reliability as some factor loadings exerted negative effects.

#### Ingenuity pathway analysis

Before examining the relation of each of the factors to the infant outcomes, we conducted Ingenuity Aathway Analysis (IPA, Qiagen) to identify core physiological functions of the miRNAs within each factor^28^. Briefly, for the _*ex*_miRNAs within each factor, the IPA miRNA Target Filter was used to identify target mRNAs with (a) high predicted confidence of interaction with PAE-sensitive _*ex*_miRNAs (context score < − 0.4, as defined previously^137^) or (b) experimentally validated miRNA/mRNA interactions. These targets were then subjected to IPA's Core Analysis workflow to identify known gene regulatory networks that are overrepresented amongst the predicted miRNA targets.

The target mRNAs for the miRNAs in each factor were then subjected to pathway enrichment analysis. In this analysis target mRNAs are assessed for overrepresentation in biological pathways against a reference data set list of genes to determine pathways that were significantly associated with the target mRNAs of the CFA-linked miRNAs compared to the universe of all possible pathways. This procedure allowed us to control for both experimental and curation-based biases in pathway overrepresentation. Pathways were considered overrepresented if they had a significantly higher proportion of the target mRNAs within the pathway than outside the pathway, determined using a right-tailed Fisher's Exact Test false discovery rate adjusted *p*-value of < 0.05. IPA’s comparison analysis tool was used to visualize and hierarchically cluster both canonical pathways and overarching disease and biofunction pathways for each timepoint.

#### Mediation analysis

Mediation analysis was performed to examine the degree to which effects of PAE on growth and cognitive outcomes were mediated by the _*ex*_miRNA expression levels of each of the factors generated by the CFAs at T_2wk_ and T_6.5mo_^138^. Mediation by miRNAs of the effects of PAE on the outcomes (i.e., the indirect effect) was estimated as the product of two regression slopes, one predicting the miRNA expression levels in each factor from PAE and one predicting the outcome from the expression levels. The significance of each indirect effect was tested by use of a *z*-test and also bootstrapping as described below. All models tested for linear relations using a saturated regression analysis model. However, because the distribution of indirect effects is likely non-normal, particularly with modest sample sizes^139^, bias-corrected non-symmetric 95% confidence intervals were created using bootstrap standard errors from 10,000 replicated samples^140^. Those results agreed with the results from the *z*-test testing the significance of the indirect effect across all instances. Mediation of the effect of PAE on each growth and cognitive outcome by each of the factors generated by the CFAs at T_2wk_ and T_6.5mo_ was examined in separate models.

Power for each mediation model was estimated using a Monte Carlo simulation with paths being set to a standardized value of 0.40, the Cohen’s *d* effect size used for the initial group comparisons and by fixing item means and variances to 0 and 1, respectively. Based on the sample size at T_2wk_ (*n* = 58) and 10,000 replications, coverage of the direct and indirect paths ranged from 93.7 to 94.5%. Power ranged from 80.4 to 85.3%. The largest parameter bias estimate was 0.005, which was well below 1% and far below 5–10%, the recommended cutoff values. Power was slightly greater for the larger sample at 6.5 months. All analyses were conducted using Mplus 8.1, except Bartlett’s correction for which we utilized R version 3.4.4.

### Ethics approval and consent to participate

Human subjects’ approval was obtained from the ethics committees at Wayne State University, the University of Cape Town Faculty of Health Sciences, and Texas A&M University. All mothers provided written informed consent.

## Supplementary Information

Supplementary Information 1.

Supplementary Information 2.

Supplementary Information 3.

Supplementary Information 4.

Supplementary Information 5.

Supplementary Information 6.

Supplementary Information 7.

Supplementary Information 8.

Supplementary Information 9.

Supplementary Information 10.

Supplementary Information 11.

Supplementary Information 12.

## Data Availability

miRNA expression data generated during this study are included in this published article as supplementary information files (see Supplementary Dataset S11) and are available in the NCBI/GEO database (accession number, GSE164209).

## Abbreviations

AA: Absolute alcohol
AIC: Akaike’s information criterion
ANOVA: Analysis of variance
ANCOVA: Analysis of covariance
BESD: Binomial effect size display
BIC: Bayesian interference criterion
CFA: Confirmatory factor analysis
CFI: Comparative fit index
CI: Confidence interval
_*ex*_miRNAs: Extracellular circulating microRNAs
ΔCT: Normalized cycle threshold
ΔΔCT: Difference in ΔCT between PAE and control
GA: Gestational age
HC: Head circumference
HE: Heavily exposed nonsyndromal
IPA: Ingenuity pathway analysis
FAS: Fetal alcohol syndrome
FASD: Fetal alcohol spectrum disorders
FTII: Fagan Test of Infant Intelligence
miRNA: MicroRNA
MIMAT: MiRbase unique accession number for microRNAs
PAE: Prenatal alcohol exposure
PFAS: Partial fetal alcohol syndrome
RSMEA: Root nean square error of approximation
SABIC: Sample-size-adjusted Baysian information criterion
TLI: Tucker–Lewis Index
