# Supplementary figures and images for: Infant circulating MicroRNAs as biomarkers of effect in fetal alcohol spectrum disorders
Source: Sci Rep. 2021 Jan 14;11:1429. doi: 10.1038/s41598-020-80734-y (PMC7809131; doi:10.1038/s41598-020-80734-y)

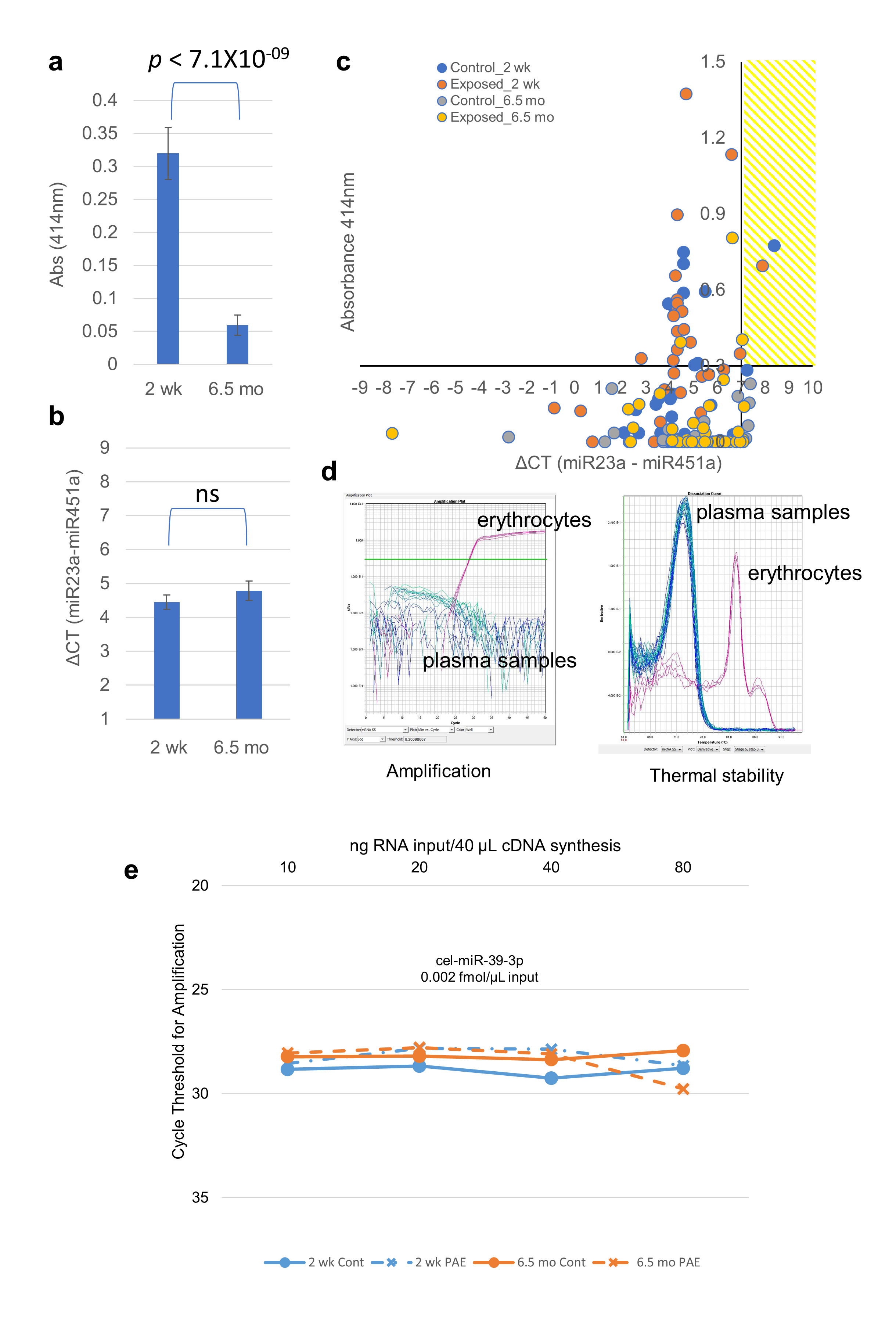

Supplement: Supplementary file 1 — Supplementary Information 1. [file 41598_2020_80734_MOESM1_ESM.tif]

**T<sub>2wk</sub> Control**

Control

**T<sub>2wk</sub>**  
**PAE**

**T<sub>2wk</sub>**  
**PAE**

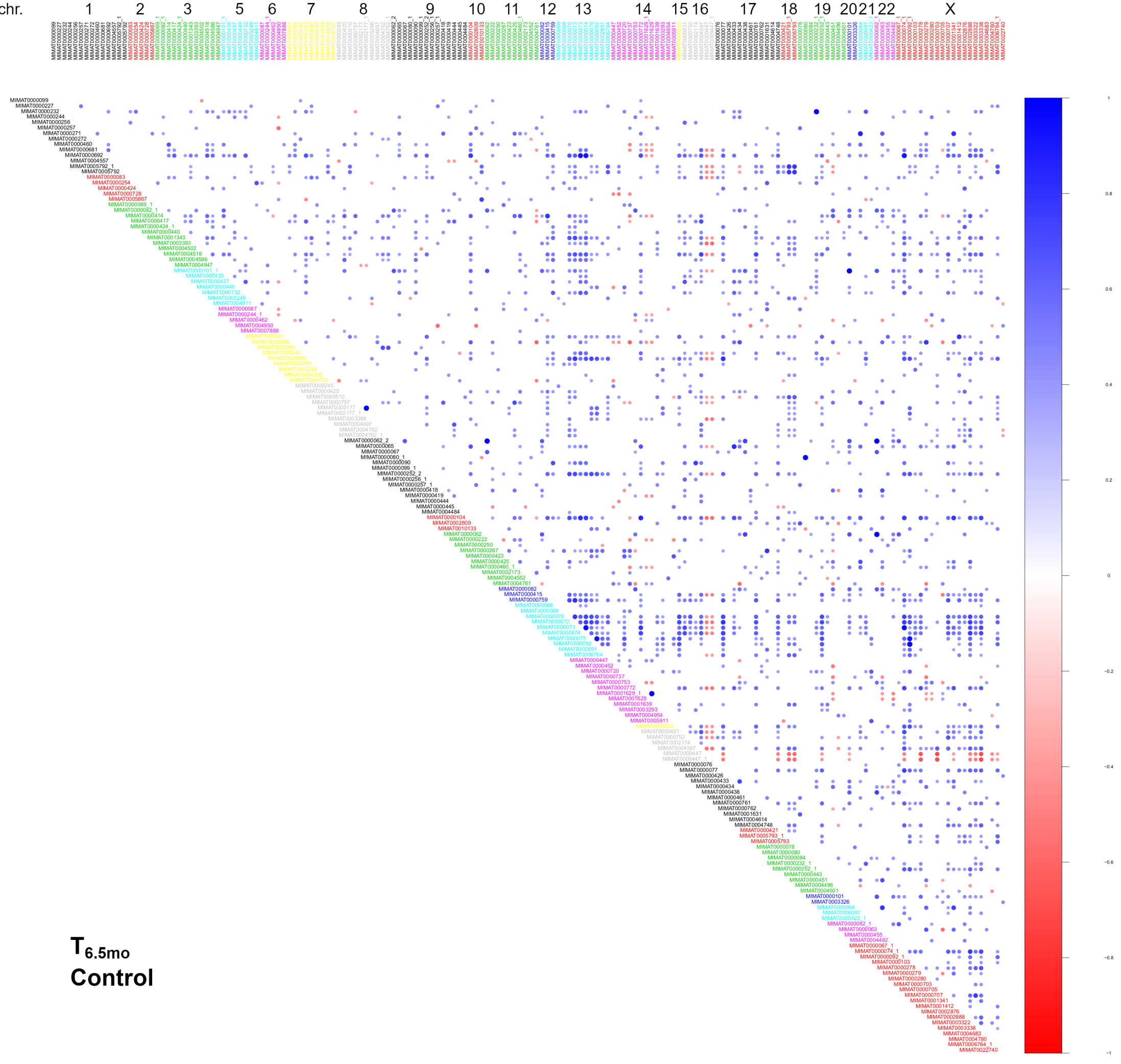

chr. 1 2 3 5 6 7 8 9 10 11 12 13 14 15 16 17 18 19 20 21 22 X

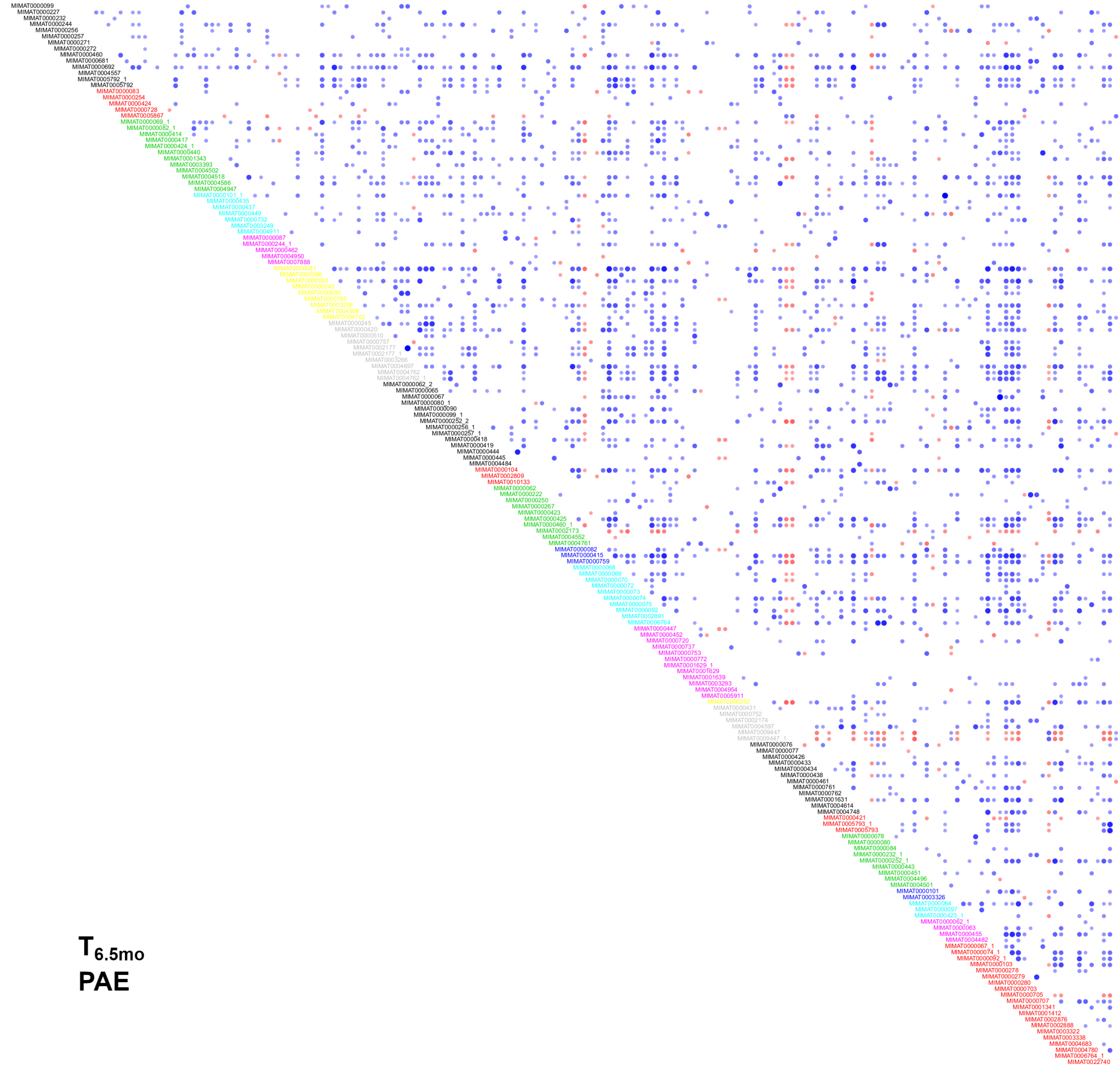

T<sub>6.5mo</sub>  
PAE

Supplement: Supplementary file 3 — Supplementary Information 3. [file 41598_2020_80734_MOESM3_ESM.pdf]

**Supplementary Figure S6 – Descriptive Fit Indices**

**a.**

**T0**


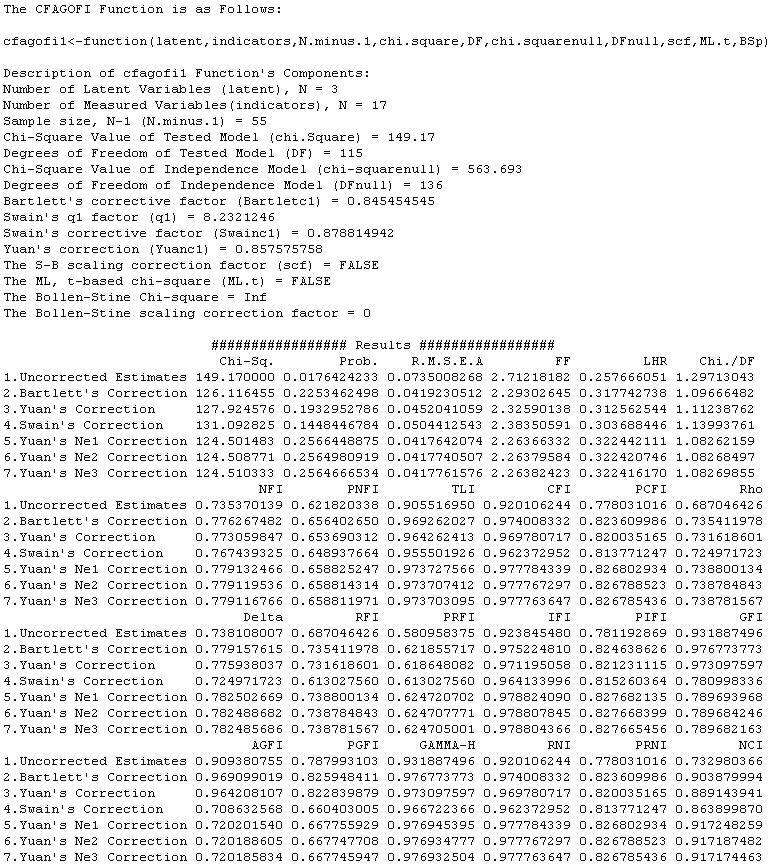


**b.**

**T0**


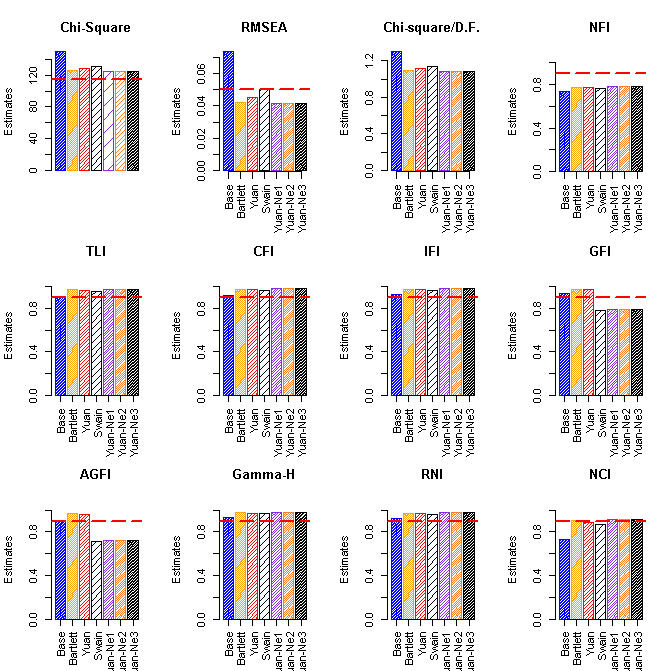


**c.**

**T6**


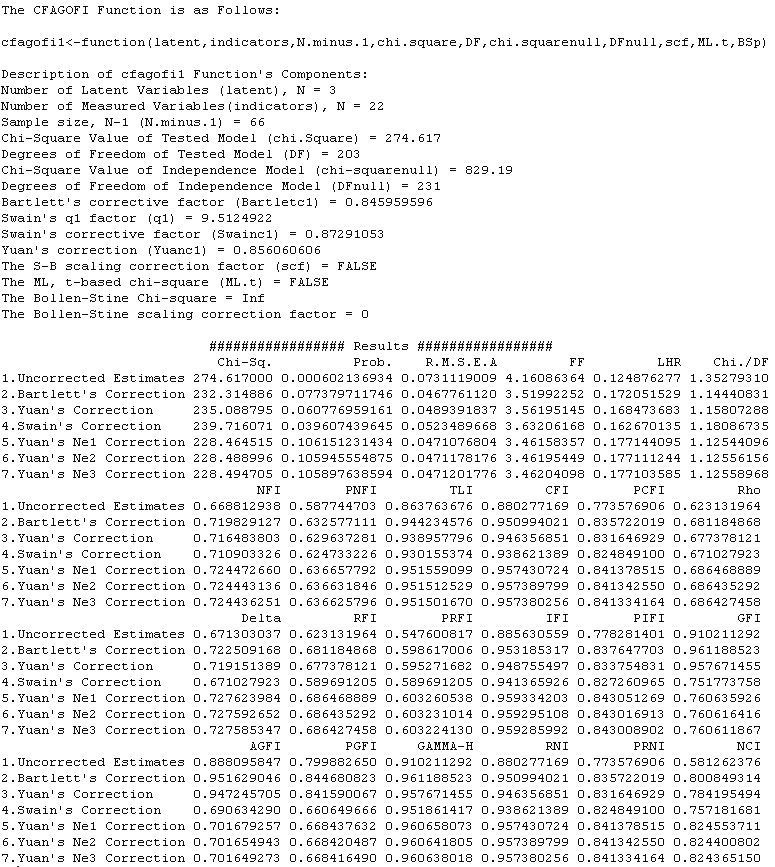


**d.**

**T6**


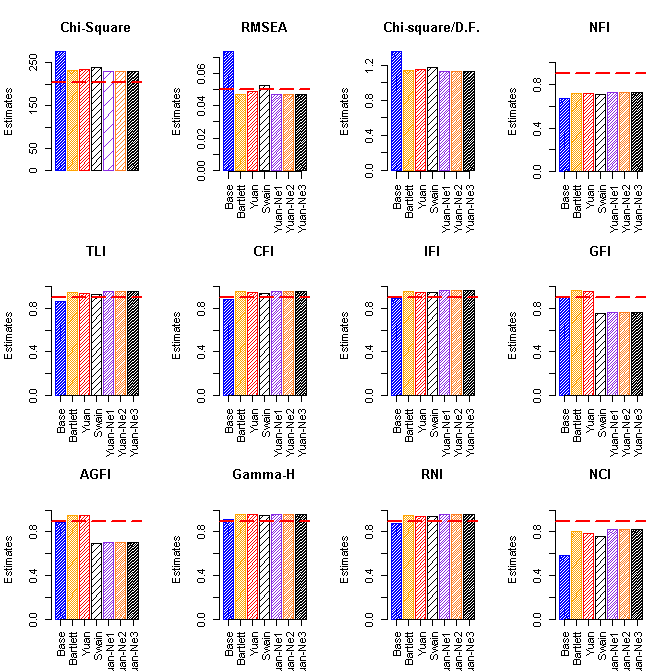

Supplement: Supplementary file 6 — Supplementary Information 6. [file 41598_2020_80734_MOESM6_ESM.docx]
