# Supplementary material for: Infant circulating MicroRNAs as biomarkers of effect in fetal alcohol spectrum disorders
Source: Sci Rep. 2021 Jan 14;11:1429. doi: 10.1038/s41598-020-80734-y (PMC7809131; doi:10.1038/s41598-020-80734-y)
Supplement: Supplementary file 5 — Supplementary Information 5. [file 41598_2020_80734_MOESM5_ESM.pdf]

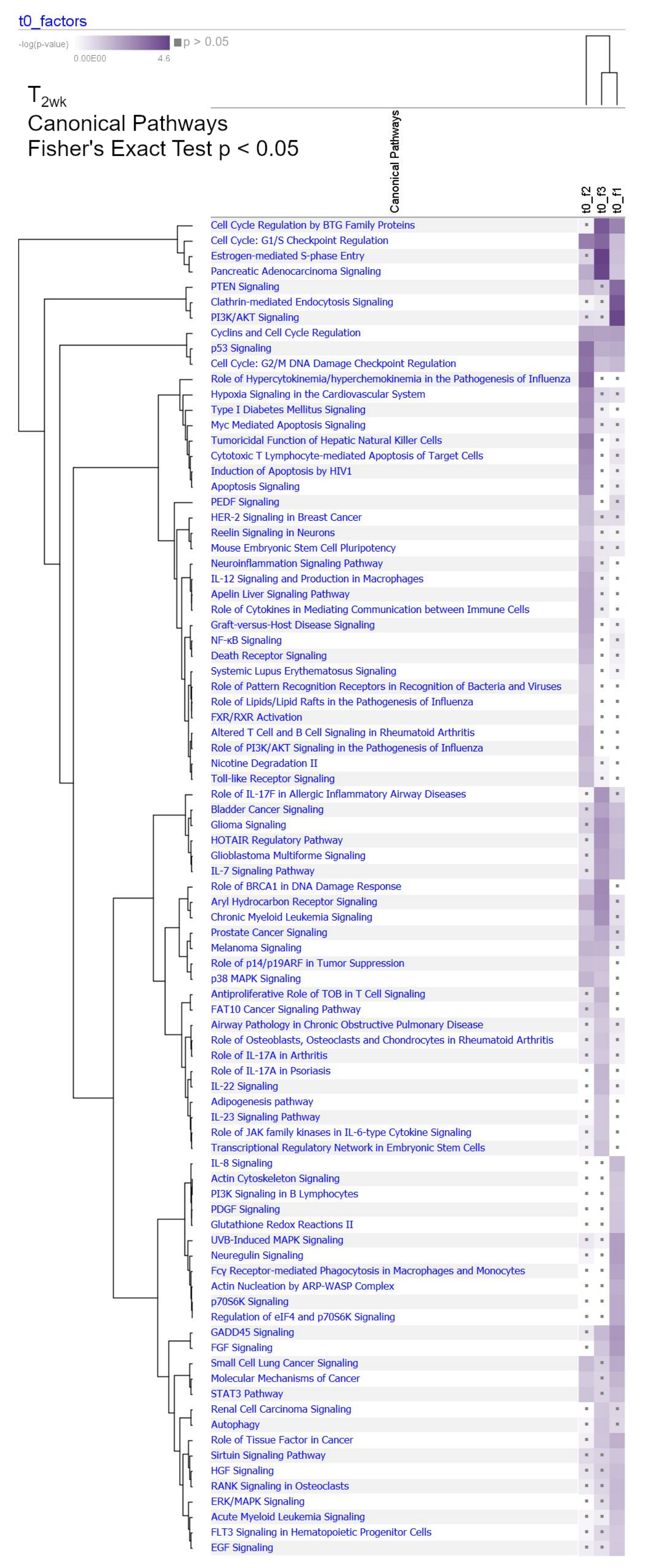
t0_factors
-log(p-value)
0.00E00
4.6
p > 0.05
T_{2wk}
Canonical Pathways
Fisher's Exact Test p < 0.05
Canonical Pathways
t0_f2
t0_f3
t0_f1
Cell Cycle Regulation by BTG Family Proteins
Cell Cycle: G1/S Checkpoint Regulation
Estrogen-mediated S-phase Entry
Pancreatic Adenocarcinoma Signaling
PTEN Signaling
Clathrin-mediated Endocytosis Signaling
PI3K/AKT Signaling
Cyclins and Cell Cycle Regulation
p53 Signaling
Cell Cycle: G2/M DNA Damage Checkpoint Regulation
Role of Hypercytokinemia/hyperchemokinemia in the Pathogenesis of Influenza
Hypoxia Signaling in the Cardiovascular System
Type I Diabetes Mellitus Signaling
Myc Mediated Apoptosis Signaling
Tumoricidal Function of Hepatic Natural Killer Cells
Cytotoxic T Lymphocyte-mediated Apoptosis of Target Cells
Induction of Apoptosis by HIV1
Apoptosis Signaling
PEDF Signaling
HER-2 Signaling in Breast Cancer
Reelin Signaling in Neurons
Mouse Embryonic Stem Cell Pluripotency
Neuroinflammation Signaling Pathway
IL-12 Signaling and Production in Macrophages
Apelin Liver Signaling Pathway
Role of Cytokines in Mediating Communication between Immune Cells
Graft-versus-Host Disease Signaling
NF-κB Signaling
Death Receptor Signaling
Systemic Lupus Erythematosus Signaling
Role of Pattern Recognition Receptors in Recognition of Bacteria and Viruses
Role of Lipids/Lipid Rafts in the Pathogenesis of Influenza
FXR/RXR Activation
Altered T Cell and B Cell Signaling in Rheumatoid Arthritis
Role of PI3K/AKT Signaling in the Pathogenesis of Influenza
Nicotine Degradation II
Toll-like Receptor Signaling
Role of IL-17F in Allergic Inflammatory Airway Diseases
Bladder Cancer Signaling
Glioma Signaling
HOTAIR Regulatory Pathway
Glioblastoma Multiforme Signaling
IL-7 Signaling Pathway
Role of BRCA1 in DNA Damage Response
Aryl Hydrocarbon Receptor Signaling
Chronic Myeloid Leukemia Signaling
Prostate Cancer Signaling
Melanoma Signaling
Role of p14/p19ARF in Tumor Suppression
p38 MAPK Signaling
Antiproliferative Role of TOB in T Cell Signaling
FAT10 Cancer Signaling Pathway
Airway Pathology in Chronic Obstructive Pulmonary Disease
Role of Osteoblasts, Osteoclasts and Chondrocytes in Rheumatoid Arthritis
Role of IL-17A in Arthritis
Role of IL-17A in Psoriasis
IL-22 Signaling
Adipogenesis pathway
IL-23 Signaling Pathway
Role of JAK family kinases in IL-6-type Cytokine Signaling
Transcriptional Regulatory Network in Embryonic Stem Cells
IL-8 Signaling
Actin Cytoskeleton Signaling
PI3K Signaling in B Lymphocytes
PDGF Signaling
Glutathione Redox Reactions II
UVB-Induced MAPK Signaling
Neuregulin Signaling
Fcγ Receptor-mediated Phagocytosis in Macrophages and Monocytes
Actin Nucleation by ARP-WASP Complex
p70S6K Signaling
Regulation of eIF4 and p70S6K Signaling
GADD45 Signaling
FGF Signaling
Small Cell Lung Cancer Signaling
Molecular Mechanisms of Cancer
STAT3 Pathway
Renal Cell Carcinoma Signaling
Autophagy
Role of Tissue Factor in Cancer
Sirtuin Signaling Pathway
HGF Signaling
RANK Signaling in Osteoclasts
ERK/MAPK Signaling
Acute Myeloid Leukemia Signaling
FLT3 Signaling in Hematopoietic Progenitor Cells
EGF Signaling

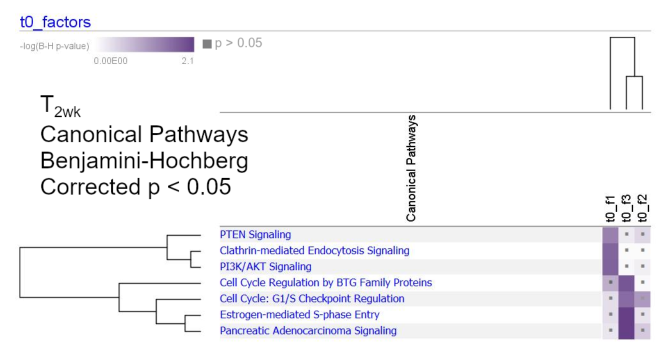
t0_factors
-log(B-H p-value)
0.00E00
2.1
p > 0.05
T2wk
Canonical Pathways
Benjamini-Hochberg
Corrected p < 0.05
Canonical Pathways
t0_f1
t0_f3
t0_f2
PTEN Signaling
Clathrin-mediated Endocytosis Signaling
PI3K/AKT Signaling
Cell Cycle Regulation by BTG Family Proteins
Cell Cycle: G1/S Checkpoint Regulation
Estrogen-mediated S-phase Entry
Pancreatic Adenocarcinoma Signaling

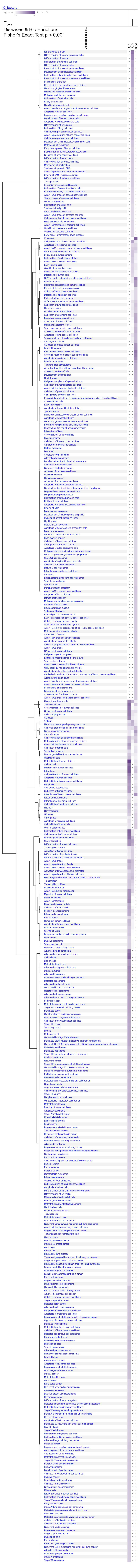
t0_factors
-log(p-value)
1.8
7.8
p > 0.05
T2wk
Diseases & Bio Functions
Fisher's Exact Test p < 0.001
Diseases and Bio ...
t0_f1
t0_f3
t0_f2
Re-entry into S phase
Differentiation of muscle precursor cells
Differentiation of muscle
Proliferation of epithelial cell lines
Differentiation of muscle cells
Re-entry into S phase of tumor cell lines
Development of hematopoietic system
Proliferation of keratinocyte cancer cell lines
Re-entry into S phase of bone cancer cell lines
Permeability transition
Re-entry into S phase of sarcoma cell lines
Hereditary gingival fibromatosis
Necrosis of vascular endothelial cells
Malignant gallbladder neoplasm
Proliferation of epithelial cells
Biliary tract cancer
Quantity of apoptotic cells
Arrest in cell cycle progression of lung cancer cell lines
Apoptosis of heart cell lines
Progesterone receptor negative breast tumor
Development of hematopoietic cells
Apoptosis of connective tissue cells
Differentiation of myoblasts
Proliferation of lung cell lines
Cell flattening of bone cancer cell lines
Arrest in proliferation of bone cancer cell lines
Cell flattening of sarcoma cell lines
Development of hematopoietic progenitor cells
Metabolism of eicosanoid
Entry into S phase of tumor cell lines
Biosynthesis of polyunsaturated fatty acids
G1 phase of bone cancer cell lines
Differentiation of osteoclasts
Cell proliferation of breast cell lines
Morphology of neutrophils
Synthesis of genomic DNA
Arrest in proliferation of sarcoma cell lines
Binding of cAMP response element
Differentiation of leukocyte cell lines
Telangiectasis
Formation of osteoclast-like cells
Proliferation of connective tissue cells
Extrahepatic biliary tract adenocarcinoma
Arrest in G1 phase of bone cancer cell lines
Shape change of sarcoma cell lines
Uptake of thymidine
Proliferation of dermal cells
Synthesis of fatty acid
Autosomal recessive ataxia
Arrest in G1 phase of sarcoma cell lines
Cell movement of bladder cancer cell lines
Head and neck adenocarcinoma
Arrest in interphase of sarcoma cell lines
Quantity of bone cancer cell lines
Quantity of sarcoma cell lines
Early-onset inflammatory bowel disease
Cytostasis
Cell proliferation of ovarian cancer cell lines
Apoptosis of hepatoma cell lines
Arrest in G0 phase of colorectal cancer cell lines
Interphase of bone cancer cell lines
Biliary tract adenocarcinoma
Proliferation of endocrine cell lines
Arrest in G1 phase of tumor cells
Entry into S phase
Growth of connective tissue
Arrest in interphase of tumor cells
Interphase of tumor cells
G1/S phase transition of breast cancer cell lines
Bile duct cancer
Premature senescence of tumor cell lines
Re-entry into cell cycle progression
S phase of breast cancer cell lines
Interphase of fibroblast cell lines
Endometrial serous carcinoma
G1/S phase transition of tumor cell lines
Cell death of lung cancer cell lines
Hereditary cancer
Depolarization of mitochondria
Cell death of carcinoma cell lines
Premature senescence of cells
Cytostasis of tumor cell lines
Malignant neoplasm of eye
Senescence of breast cancer cell lines
Cytotoxic reaction of tumor cell lines
Apoptosis of lung cancer cell lines
Serous or clear cell malignant endometrial tumor
Cholangiocarcinoma
G1 phase of breast cancer cell lines
Familial lung cancer
Response of breast cancer cell lines
Cytotoxic reaction of breast cancer cell lines
Apoptosis of carcinoma cell lines
Bile duct carcinoma
Temporal lobe astrocytoma
Activated B-cell-like diffuse large B-cell lymphoma
Cytotoxic reaction of cells
Development of fibroblasts
Orbital tumor
Malignant neoplasm of eye and adnexa
Cell death of lymphoblastoid cell lines
Arrest in interphase of fibroblast cell lines
Cell death of gonadal cell lines
Clonogenicity of tumor cell lines
Extranodal marginal zone lymphoma of mucosa-associated lymphoid tissue
Cytotoxicity of cells
Entry into mitosis
Apoptosis of lymphoblastoid cell lines
Sporadic tumor
Premature senescence of breast cancer cell lines
Apoptosis of gonadal cell lines
Hereditary gastrointestinal cancer syndrome
B-cell non-Hodgkin lymphoma in lymph node
Phospholipid flip-flop of phosphatidylserine
Interaction of DNA
Cytotoxicity of tumor cell lines
B-cell neoplasm
Cell death of fibrosarcoma cell lines
Generation of dermal fibroblasts
Richter syndrome
Leukemia
Contact growth inhibition
Adrenal cortex carcinoma
Depolarization of mitochondrial membrane
Cell death of carcinoma cells
Refractory multiple myeloma
G1 phase of carcinoma cell lines
Myeloid neoplasm
Hematologic cancer
G2 phase of bone cancer cell lines
Apoptosis of B lymphoblastoid cell lines
Germinal center B-cell-like diffuse large B-cell lymphoma
Large cell neuroendocrine carcinoma
Lymphohematopoietic cancer
Proliferation of smooth muscle cells
Ploidy of tumor cell lines
Apoptosis of rhabdomyosarcoma cell lines
Binding of DNA
Bone marrow neoplasm
Development of antigen presenting cells
Invasion of breast cancer cell lines
Liquid tumor
Mature B-cell neoplasm
Apoptosis of hematopoietic progenitor cells
Bone osteosarcoma
Immune response of tumor cell lines
Bone marrow cancer
Cell death of hepatoma cell lines
G2/M phase of tumor cell lines
Apoptosis of colon carcinoma cells
Malignant fibrous histiocytoma in fibrous tissue
Diffuse large B-cell lymphoma in lymph node
Colon tubular adenoma
Apoptosis of erythroid precursor cells
Cell death of sarcoma cell lines
Mature B-cell lymphoma
Interphase of carcinoma cell lines
Adenoma
Extranodal marginal zone cell lymphoma
Small intestine tumor
Sporadic cancer
Lymphoreticular neoplasm
Arrest in G2 phase of tumor cell lines
Apoptosis of lung cell lines
Diffuse gastric cancer
Malignant endometrial serous neoplasm
Initiation of interphase
Fragmentation of nucleus
S phase of fibroblasts
Familial gastric or colon cancer
Entry into mitosis of cervical cancer cell lines
Cell death of ovarian cancer cells
Grade 4 supratentorial astrocytoma
Arrest in cell cycle progression of colorectal cancer cell lines
Metabolism of phosphatidylcholine
Catabolism of steroid
Arrest in M phase of tumor cell lines
Apoptosis of synovial fibroblasts
Cell cycle progression of colorectal cancer cell lines
Arrest in G2 phase
G2 phase of tumor cell lines
Malignant myeloid neoplasm
Epithelioid mesothelioma in lung pleura
Suppression of tumor
Arrest in G2 phase of fibroblast cell lines
WHO grade IV malignant astrocytoma
Apoptosis of distal lung epithelial cells
Antibody-dependent cell-mediated cytotoxicity of breast cancer cell lines
Adenocarcinoma in ileum
Arrest in cell cycle progression of melanoma cell lines
Arrest in mitosis of colorectal cancer cell lines
Permeability of mitochondria
Benign neoplasm of pancreas
Cytotoxicity of fibroblast cell lines
Arrest in G1 phase of bladder cancer cell lines
Colony formation of cells
Synthesis of DNA
Colony formation of tumor cell lines
G1 phase of tumor cell lines
Cell cycle progression
G1 phase
S phase
Hereditary cancer-predisposing syndrome
Cell cycle progression of tumor cell lines
Liver cholangiocarcinoma
Cervical cancer
Cell proliferation of carcinoma cell lines
Cell proliferation of breast cancer cell lines
Arrest in interphase of tumor cell lines
Cell death of tumor cells
Survival of organism
Female genital tract serous carcinoma
Quantity of cells
Cell viability of tumor cell lines
Cell survival
Interphase of tumor cell lines
Interphase
Cell proliferation of tumor cell lines
Apoptosis of tumor cell lines
Cell viability of breast cancer cell lines
Apoptosis
Connective tissue cancer
Cell death of tumor cell lines
Interphase of breast cancer cell lines
Rectal adenocarcinoma
Interphase of leukemia cell lines
Cell viability of carcinoma cell lines
Necrosis
Osteosarcoma
G2 phase
G2/M phase
Apoptosis of sarcoma cell lines
Cell viability of tumor cells
Uterine corpus cancer
Proliferation of lung cancer cell lines
Cell movement of tumor cell lines
Morphology of tumor cell lines
Colony formation
Differentiation of tumor cell lines
Transcription of DNA
Activation of tumor cell lines
Differentiation of epithelial tissue
Interphase of colorectal cancer cell lines
Arrest in G1 phase
Arrest in proliferation of cells
Arrest in G1 phase of tumor cell lines
Activation of DNA endogenous promoter
Arrest in proliferation of tumor cell lines
HER2 negative hormone receptor negative breast cancer
Transcription
Transcription of RNA
Mesenchymal tumor
Arrest in cell cycle progression
Migration of tumor cell lines
Primary carcinoma
Arrest in interphase
Phosphorylation of protein
Cell death of cancer cells
Papillary adenocarcinoma
Primary adenocarcinoma
Endometriosis
Homing of tumor cell lines
Apoptosis of breast cancer cell lines
Fibrous tissue tumor
Growth of axons
Benign connective or soft tissue neoplasm
Pelvic tumor
Invasive carcinoma
Senescence of cells
Formation of secondary tumor
Advanced stage carcinoma
Advanced extracranial solid tumor
Cell viability
Size of cells
Metastatic lung tumor
Advanced malignant solid tumor
Stage I-II tumor
Advanced lung cancer
Metastatic non-small cell lung carcinoma
Metastatic carcinoma
Advanced malignant tumor
Unresectable recurrent cancer
Hepatocellular carcinoma
Advanced adenocarcinoma
Advanced non-small cell lung carcinoma
Pediatric cancer
Metastatic unresectable malignant tumor
Stage I-IV non-small cell lung cancer
Stage IIIB cancer
Undifferentiated malignant neoplasm
BRAF mutation negative solid tumor
Cell death of cervical cancer cell lines
Stage IIIC cancer
Secondary tumor
Mitosis
Cell movement
Unresectable stage IIIC melanoma
Stage IIIB BRAF mutation negative cutaneous melanoma
Unresectable BRAF mutation negative NRAS mutation negative melanoma
Metastatic solid tumor
Stage IIIC melanoma
Stage IIIB metastatic cutaneous melanoma
Papillary carcinoma
Recurrent cancer
Stage IIIB unresectable metastatic melanoma
Unresectable stage III cutaneous melanoma
Stage 3B unresectable cutaneous melanoma
Epithelial-mesenchymal transition
Metastatic adenocarcinoma
Metastatic unresectable malignant solid tumor
Organismal death
Organization of cellular membrane
Cell movement of colorectal cancer cell lines
Stage I-II cancer
Neoplasia of tumor cell lines
Unresectable metastatic solid tumor
Metastatic melanoma
Invasion of tumor cell lines
Anaplastic carcinoma
Stage IV malignant tumor
Musculoskeletal cancer
Large-cell carcinoma
Pelvic cancer
Progressive metastatic carcinoma
Tubular adenocarcinoma
Refractory malignant solid tumor
Cell death of mammary tumor cells
Metastatic large cell lung carcinoma
Advanced liver tumor
Progressive squamous cell lung cancer
Stage IIIB nonsquamous non-small cell lung carcinoma
Genitourinary carcinoma
Recurrent carcinoma
Childhood malignant hematological system tumor
Benign Tumors
Rectum cancer
Stage II cancer
Unresectable melanoma
Primary colon cancer
Quantity of focal adhesions
Cell proliferation of brain cancer cell lines
Apoptosis of retinal cells
Differentiation of central nervous system cells
Differentiation of neuroglia
Mitogenesis of endothelial cells
Female genital tract cancer
Metastatic gastrointestinal carcinoma
Haptotaxis of cells
Diabetic macular edema
Tubulogenesis
Metastatic renal cancer
Metastatic renal cell carcinoma
Recurrent nonsquamous non-small cell lung carcinoma
Arrest in interphase of lung cancer cell lines
Progressive ALK fusion positive solid tumor
Tumorigenesis of reproductive tract
Uterine tumor
Female genital neoplasm
Stage II-IV breast cancer
Autophagy
Benign lesion
Progressive lung disease
Tumor antigen-positive non-small cell lung carcinoma
Stage II-IV gastrointestinal tract cancer
Progressive nonsquamous non-small cell lung carcinoma
Female genital tract adenocarcinoma
Metastatic thyroid carcinoma
Locally recurrent malignant solid tumor
Recurrent leukemia
Progressive advanced cancer
Lung squamous cell carcinoma
Unresectable metastasis
Recurrent non-small cell lung cancer
Advanced squamous cell cancer
Cell death of ovarian cancer cell lines
Stage IV epithelial cancer
Metastatic skin cancer
Advanced soft tissue sarcoma
Apoptosis of cervical cancer cell lines
Apoptosis of melanoma cell lines
Progressive metastatic non-small cell lung carcinoma
Migration of colorectal cancer cell lines
Stage III-IV melanoma
Cell viability of lung cancer cell lines
Cell death of breast cancer cell lines
Metastatic squamous cell carcinoma
Early stage solid tumor
Metastatic soft tissue sarcoma
Migration of cells
Subcutaneous tumor
Advanced pancreatic tumor
Primary colorectal adenocarcinoma
Familial tumor
Benign pelvic disease
Apoptosis of leukemia cell lines
Progressive metastatic lung cancer
HER2-negative breast cancer
Stage I cancer
Metastatic skin tumor
Brain cancer
Early stage tumor
Recurrent head and neck carcinoma
Metastatic sarcoma
Invasive breast adenocarcinoma
Rectum carcinoma
Differentiation of nervous system
Metastatic malignant connective or soft tissue neoplasm
Cell viability of cervical cancer cell lines
Stage IV non-squamous lung carcinoma
Stage IV advanced non-small cell lung carcinoma
Recurrent sarcoma
Apoptosis of brain cancer cell lines
Stage IIIB-IV recurrent non-small cell lung cancer
B-cell leukemia
Stage IV solid tumor
Proliferation of myeloma cell lines
Proliferation of kidney cancer cell lines
Advanced large cell lung carcinoma
Stage III cancer
Progesterone receptor-negative breast cancer
Autophagy of colorectal cancer cell lines
Chemotaxis of tumor cell lines
Metastatic pancreatic neoplasm
Stage III-IV metastatic melanoma
Stage IV advanced solid tumor
Primary neoplasm
Development of genital tumor
Cell death of colorectal cancer cell lines
Invasive cancer
Familial nephrotic syndrome
Cell death of granule cells
Genitourinary adenocarcinoma
Mitogenesis
Radioresistance of tumor cell lines
Proliferation of embryonic cancer cell lines
Stage IV non-small cell lung carcinoma
Early breast cancer
Stage IV lung squamous cell carcinoma
Metastatic progressive malignant solid tumor
Idiopathic scoliosis
Metastatic unresectable advanced malignant tumor
Cell death of leukemia cell lines
Cell death of melanoma cell lines
Recurrent acute leukemia
Progressive recurrent neoplasm
Stage I epithelial cancer
Invasion of cells
Rectum tumor
Breast or gynecological cancer
Recurrent EGFR expressing non-small cell lung cancer
Adhesion of kidney cells
Metastatic progressive tumor
Stage IV melanoma
Stage III melanoma

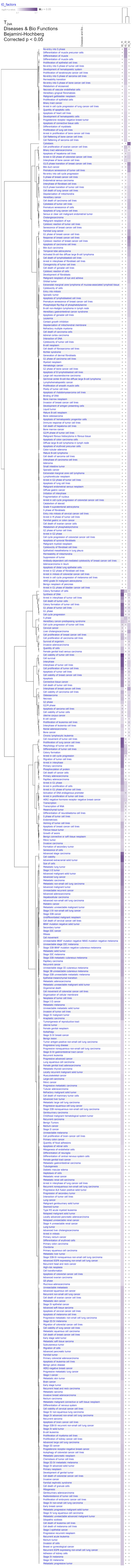
t0_factors
-log(B-H p-value)
0.7
3.7
p > 0.05
T2wk
Diseases & Bio Functions
Bejamini-Hochberg
Corrected p < 0.05
Diseases and Bio...
t0_f1
t0_f3
t0_f2
Re-entry into S phase
Differentiation of muscle precursor cells
Differentiation of muscle
Differentiation of muscle cells
Proliferation of epithelial cell lines
Re-entry into S phase of tumor cell lines
Development of hematopoietic system
Proliferation of keratinocyte cancer cell lines
Re-entry into S phase of sarcoma cell lines
Permeability transition
Re-entry into S phase of bone cancer cell lines
Metabolism of eicosanoid
Necrosis of vascular endothelial cells
Hereditary gingival fibromatosis
Malignant gallbladder neoplasm
Proliferation of epithelial cells
Biliary tract cancer
Arrest in cell cycle progression of lung cancer cell lines
Quantity of apoptotic cells
Apoptosis of heart cell lines
Development of hematopoietic cells
Progesterone receptor negative breast tumor
Apoptosis of connective tissue cells
Differentiation of myoblasts
Proliferation of lung cell lines
Arrest in proliferation of bone cancer cell lines
Cell flattening of bone cancer cell lines
Cell flattening of sarcoma cell lines
Cytostasis
Cell proliferation of ovarian cancer cell lines
Biliary tract adenocarcinoma
Apoptosis of hepatoma cell lines
Arrest in G0 phase of colorectal cancer cell lines
Interphase of bone cancer cell lines
G1/S phase transition of breast cancer cell lines
Bile duct cancer
Premature senescence of tumor cell lines
Re-entry into cell cycle progression
S phase of breast cancer cell lines
Endometrial serous carcinoma
Interphase of fibroblast cell lines
G1/S phase transition of tumor cell lines
Cell death of lung cancer cell lines
Depolarization of mitochondria
Hereditary cancer
Cell death of carcinoma cell lines
Cytostasis of tumor cell lines
Premature senescence of cells
Apoptosis of lung cancer cell lines
Serous or clear cell malignant endometrial tumor
Cholangiocarcinoma
Malignant neoplasm of eye
Cytotoxic reaction of tumor cell lines
Senescence of breast cancer cell lines
Familial lung cancer
G1 phase of breast cancer cell lines
Response of breast cancer cell lines
Cytotoxic reaction of breast cancer cell lines
Apoptosis of carcinoma cell lines
Bile duct carcinoma
Temporal lobe astrocytoma
Activated B-cell-like diffuse large B-cell lymphoma
Cell death of lymphoblastoid cell lines
Arrest in interphase of fibroblast cell lines
Clonogenicity of tumor cell lines
Cell death of gonadal cell lines
Cytotoxic reaction of cells
Development of fibroblasts
Malignant neoplasm of eye and adnexa
Orbital tumor
Extranodal marginal zone lymphoma of mucosa-associated lymphoid tissue
Cytotoxicity of cells
Entry into mitosis
Sporadic tumor
Apoptosis of lymphoblastoid cell lines
Premature senescence of breast cancer cell lines
Phospholipid flip-flop of phosphatidylserine
B-cell non-Hodgkin lymphoma in lymph node
Hereditary gastrointestinal cancer syndrome
Apoptosis of gonadal cell lines
Leukemia
Contact growth inhibition
Depolarization of mitochondrial membrane
Refractory multiple myeloma
Cell death of carcinoma cells
Adrenal cortex carcinoma
Interaction of DNA
Cytotoxicity of tumor cell lines
B-cell neoplasm
Cell death of fibrosarcoma cell lines
Richter syndrome
Generation of dermal fibroblasts
G1 phase of carcinoma cell lines
Myeloid neoplasm
Hematologic cancer
G2 phase of bone cancer cell lines
Apoptosis of B lymphoblastoid cell lines
Large cell neuroendocrine carcinoma
Germinal center B-cell-like diffuse large B-cell lymphoma
Lymphohematopoietic cancer
Proliferation of smooth muscle cells
Ploidy of tumor cell lines
Apoptosis of rhabdomyosarcoma cell lines
Binding of DNA
Bone marrow neoplasm
Invasion of breast cancer cell lines
Development of antigen presenting cells
Liquid tumor
Mature B-cell neoplasm
Bone osteosarcoma
Apoptosis of hematopoietic progenitor cells
Immune response of tumor cell lines
Cell death of hepatoma cell lines
Bone marrow cancer
G2/M phase of tumor cell lines
Malignant fibrous histiocytoma in fibrous tissue
Apoptosis of colon carcinoma cells
Diffuse large B-cell lymphoma in lymph node
Apoptosis of erythroid precursor cells
Colon tubular adenoma
Mature B-cell lymphoma
Cell death of sarcoma cell lines
Interphase of carcinoma cell lines
Adenoma
Small intestine tumor
Sporadic cancer
Extranodal marginal zone cell lymphoma
Lymphoreticular neoplasm
Arrest in G2 phase of tumor cell lines
Apoptosis of lung cell lines
Malignant endometrial serous neoplasm
Diffuse gastric cancer
Initiation of interphase
Fragmentation of nucleus
Arrest in cell cycle progression of colorectal cancer cell lines
Catabolism of steroid
Grade 4 supratentorial astrocytoma
S phase of fibroblasts
Entry into mitosis of cervical cancer cell lines
Arrest in M phase of tumor cell lines
Familial gastric or colon cancer
Cell death of ovarian cancer cells
Metabolism of phosphatidylcholine
G2 phase of tumor cell lines
Arrest in G2 phase
Cell cycle progression of colorectal cancer cell lines
Apoptosis of synovial fibroblasts
Malignant myeloid neoplasm
Cytotoxicity of fibroblast cell lines
Epithelioid mesothelioma in lung pleura
Permeability of mitochondria
Suppression of tumor
Antibody-dependent cell-mediated cytotoxicity of breast cancer cell lines
Adenocarcinoma in ileum
Apoptosis of distal lung epithelial cells
Arrest in G2 phase of fibroblast cell lines
Arrest in mitosis of colorectal cancer cell lines
Arrest in cell cycle progression of melanoma cell lines
WHO grade IV malignant astrocytoma
Benign neoplasm of pancreas
Arrest in G1 phase of bladder cancer cell lines
Colony formation of cells
Synthesis of DNA
Arrest in interphase of tumor cell lines
Cell death of tumor cells
Colony formation of tumor cell lines
G1 phase of tumor cell lines
G1 phase
Cell cycle progression
S phase
Hereditary cancer-predisposing syndrome
Cell cycle progression of tumor cell lines
Cervical cancer
Liver cholangiocarcinoma
Cell proliferation of breast cancer cell lines
Cell proliferation of carcinoma cell lines
Survival of organism
Invasive adenocarcinoma
Quantity of cells
Female genital tract serous carcinoma
Cell viability of tumor cell lines
Cell survival
Interphase
Interphase of tumor cell lines
Cell proliferation of tumor cell lines
Apoptosis of tumor cell lines
Cell viability of breast cancer cell lines
Apoptosis
Connective tissue cancer
Cell death of tumor cell lines
Interphase of breast cancer cell lines
Cell viability of carcinoma cell lines
Osteosarcoma
Necrosis
G2 phase
G2/M phase
Apoptosis of sarcoma cell lines
Cell viability of tumor cells
Uterine corpus cancer
B cell cancer
Proliferation of leukemia cell lines
Interphase of leukemia cell lines
Rectal adenocarcinoma
Bone cancer
Chronic lymphocytic leukemia
Cell movement of tumor cell lines
Proliferation of lung cancer cell lines
Morphology of tumor cell lines
Differentiation of tumor cell lines
Colony formation
Arrest in cell cycle progression
Migration of tumor cell lines
Arrest in interphase
Primary carcinoma
Phosphorylation of protein
Cell death of cancer cells
Primary adenocarcinoma
Papillary adenocarcinoma
Arrest in G1 phase
Arrest in proliferation of cells
Arrest in G1 phase of tumor cell lines
Activation of DNA endogenous promoter
Arrest in proliferation of tumor cell lines
HER2 negative hormone receptor negative breast cancer
Transcription
Transcription of RNA
Mesenchymal tumor
Differentiation of neuroblastoma cell lines
S phase of tumor cell lines
Endometriosis
Homing of tumor cell lines
Apoptosis of breast cancer cell lines
Fibrous tissue tumor
Growth of axons
Benign connective or soft tissue neoplasm
Pelvic tumor
Invasive carcinoma
Formation of secondary tumor
Senescence of cells
Advanced stage carcinoma
Cell viability
Advanced extracranial solid tumor
Size of cells
Metastatic lung tumor
Stage I-II tumor
Advanced malignant solid tumor
Advanced lung cancer
Metastatic carcinoma
Metastatic non-small cell lung carcinoma
Advanced malignant tumor
Unresectable recurrent cancer
Advanced adenocarcinoma
Hepatocellular carcinoma
Advanced non-small cell lung carcinoma
Pediatric cancer
Metastatic unresectable malignant tumor
Stage I-IV non-small cell lung cancer
Stage IIIB cancer
Undifferentiated malignant neoplasm
Cell death of cervical cancer cell lines
BRAF mutation negative solid tumor
Secondary tumor
Stage IIIC cancer
Mitosis
Cell movement
Unresectable BRAF mutation negative NRAS mutation negative melanoma
Unresectable stage IIIC melanoma
Stage IIIB BRAF mutation negative cutaneous melanoma
Metastatic solid tumor
Stage IIIC melanoma
Stage IIIB metastatic cutaneous melanoma
Papillary carcinoma
Recurrent cancer
Unresectable stage III cutaneous melanoma
Stage 3B unresectable cutaneous melanoma
Stage IIIB unresectable metastatic melanoma
Epithelial-mesenchymal transition
Metastatic adenocarcinoma
Metastatic unresectable malignant solid tumor
Organismal death
Cell movement of colorectal cancer cell lines
Organization of cellular membrane
Neoplasia of tumor cell lines
Stage I-II cancer
Metastatic melanoma
Unresectable metastatic solid tumor
Invasion of tumor cell lines
Stage IV malignant tumor
Anaplastic carcinoma
Tumorigenesis of reproductive tract
Uterine tumor
Female genital neoplasm
Autophagy
Stage II-IV breast cancer
Benign lesion
Tumor antigen-positive non-small cell lung carcinoma
Progressive lung disease
Progressive nonsquamous non-small cell lung carcinoma
Stage II-IV gastrointestinal tract cancer
Recurrent leukemia
Progressive advanced cancer
Lung squamous cell carcinoma
Female genital tract adenocarcinoma
Metastatic thyroid carcinoma
Locally recurrent malignant solid tumor
Musculoskeletal cancer
Large-cell carcinoma
Pelvic cancer
Progressive metastatic carcinoma
Tubular adenocarcinoma
Refractory malignant solid tumor
Cell death of mammary tumor cells
Advanced liver tumor
Metastatic large cell lung carcinoma
Progressive squamous cell lung cancer
Stage IIIB nonsquamous non-small cell lung carcinoma
Genitourinary carcinoma
Childhood malignant hematological system tumor
Recurrent carcinoma
Benign Tumors
Rectum cancer
Stage II cancer
Unresectable melanoma
Cell proliferation of brain cancer cell lines
Primary colon cancer
Quantity of focal adhesions
Apoptosis of retinal cells
Mitogenesis of endothelial cells
Differentiation of neuroglia
Differentiation of central nervous system cells
Female genital tract cancer
Metastatic gastrointestinal carcinoma
Tubulogenesis
Diabetic macular edema
Haptotaxis of cells
Metastatic renal cancer
Metastatic renal cell carcinoma
Arrest in interphase of lung cancer cell lines
Recurrent nonsquamous non-small cell lung carcinoma
Progressive ALK fusion positive solid tumor
Progression of secondary tumor
Interaction of tumor cell lines
Lung cancer
Malignant genitourinary solid tumor
Desmoid tumor
Type M3 acute myeloid leukemia
Relapsed malignant solid tumor
Locally advanced pancreatic adenocarcinoma
Relapsed unresectable renal cancer
Stage 4 unresectable renal cancer
Lung tumor
Advanced liver cholangiocarcinoma
Arrest in mitosis
Primary rectum cancer
Differentiation of erythroid cells
Primary colon carcinoma
Chordoma
Primary squamous cell carcinoma
Metastatic liver tumor
Stage IIIB-IV nonsquamous non-small cell lung carcinoma
Advanced EGFR expressing non-small cell lung cancer
Recurrent head and neck cancer
High-risk neoplasia
Cell transformation
Apoptosis of colorectal cancer cell lines
Advanced ovarian carcinoma
G0 phase
Mucinous adenocarcinoma
Unresectable metastasis
Advanced squamous cell cancer
Recurrent non-small cell lung cancer
Cell death of ovarian cancer cell lines
Metastatic skin cancer
Stage IV epithelial cancer
Advanced soft tissue sarcoma
Apoptosis of cervical cancer cell lines
Apoptosis of melanoma cell lines
Progressive metastatic non-small cell lung carcinoma
Stage III-IV melanoma
Migration of colorectal cancer cell lines
Cell viability of lung cancer cell lines
Metastatic squamous cell carcinoma
Cell death of breast cancer cell lines
Early stage solid tumor
Metastatic soft tissue sarcoma
Subcutaneous tumor
Migration of cells
Advanced pancreatic tumor
Familial tumor
Primary colorectal adenocarcinoma
Apoptosis of leukemia cell lines
Benign pelvic disease
HER2-negative breast cancer
Progressive metastatic lung cancer
Stage I cancer
Metastatic skin tumor
Brain cancer
Early stage tumor
Recurrent head and neck carcinoma
Metastatic sarcoma
Invasive breast adenocarcinoma
Rectum carcinoma
Metastatic malignant connective or soft tissue neoplasm
Differentiation of nervous system
Cell viability of cervical cancer cell lines
Stage IV non-squamous lung carcinoma
Stage IV advanced non-small cell lung carcinoma
Recurrent sarcoma
Apoptosis of brain cancer cell lines
Stage IIIB-IV recurrent non-small cell lung cancer
Stage IV solid tumor
B-cell leukemia
Proliferation of myeloma cell lines
Proliferation of kidney cancer cell lines
Advanced large cell lung carcinoma
Stage III cancer
Progesterone receptor-negative breast cancer
Autophagy of colorectal cancer cell lines
Metastatic pancreatic neoplasm
Chemotaxis of tumor cell lines
Stage III-IV metastatic melanoma
Stage IV advanced solid tumor
Primary neoplasm
Development of genital tumor
Cell death of colorectal cancer cell lines
Invasive cancer
Familial nephrotic syndrome
Cell death of granule cells
Mitogenesis
Genitourinary adenocarcinoma
Radioresistance of tumor cell lines
Proliferation of embryonic cancer cell lines
Stage IV non-small cell lung carcinoma
Early breast cancer
Metastatic progressive malignant solid tumor
Stage IV lung squamous cell carcinoma
Metastatic unresectable advanced malignant tumor
Idiopathic scoliosis
Cell death of leukemia cell lines
Cell death of melanoma cell lines
Stage I epithelial cancer
Progressive recurrent neoplasm
Recurrent acute leukemia
Rectum tumor
Invasion of cells
Breast or gynecological cancer
Recurrent EGFR expressing non-small cell lung cancer
Adhesion of kidney cells
Stage IV melanoma
Stage III melanoma
Metastatic progressive tumor
