# Supplementary material for: Infant circulating MicroRNAs as biomarkers of effect in fetal alcohol spectrum disorders
Source: Sci Rep. 2021 Jan 14;11:1429. doi: 10.1038/s41598-020-80734-y (PMC7809131; doi:10.1038/s41598-020-80734-y)

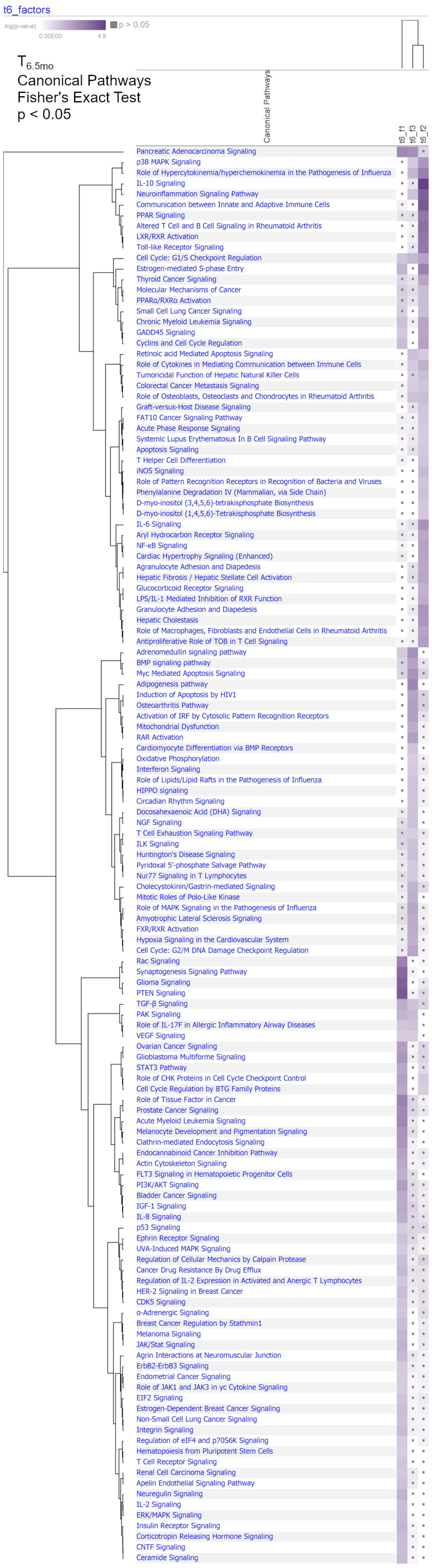
t6_factors
-log(p-value)
0.00E00
4.9
p > 0.05
T6.5mo
Canonical Pathways
Fisher's Exact Test
p < 0.05
Canonical Pathways
t6_f1
t6_f3
t6_f2
Pancreatic Adenocarcinoma Signaling
p38 MAPK Signaling
Role of Hypercytokinemia/hyperchemokinemia in the Pathogenesis of Influenza
IL-10 Signaling
Neuroinflammation Signaling Pathway
Communication between Innate and Adaptive Immune Cells
PPAR Signaling
Altered T Cell and B Cell Signaling in Rheumatoid Arthritis
LXR/RXR Activation
Toll-like Receptor Signaling
Cell Cycle: G1/S Checkpoint Regulation
Estrogen-mediated S-phase Entry
Thyroid Cancer Signaling
Molecular Mechanisms of Cancer
PPARα/RXRα Activation
Small Cell Lung Cancer Signaling
Chronic Myeloid Leukemia Signaling
GADD45 Signaling
Cyclins and Cell Cycle Regulation
Retinoic acid Mediated Apoptosis Signaling
Role of Cytokines in Mediating Communication between Immune Cells
Tumoricidal Function of Hepatic Natural Killer Cells
Colorectal Cancer Metastasis Signaling
Role of Osteoblasts, Osteoclasts and Chondrocytes in Rheumatoid Arthritis
Graft-versus-Host Disease Signaling
FAT10 Cancer Signaling Pathway
Acute Phase Response Signaling
Systemic Lupus Erythematosus In B Cell Signaling Pathway
Apoptosis Signaling
T Helper Cell Differentiation
iNOS Signaling
Role of Pattern Recognition Receptors in Recognition of Bacteria and Viruses
Phenylalanine Degradation IV (Mammalian, via Side Chain)
D-myo-inositol (3,4,5,6)-tetrakisphosphate Biosynthesis
D-myo-inositol (1,4,5,6)-Tetrakisphosphate Biosynthesis
IL-6 Signaling
Aryl Hydrocarbon Receptor Signaling
NF-κB Signaling
Cardiac Hypertrophy Signaling (Enhanced)
Agranulocyte Adhesion and Diapedesis
Hepatic Fibrosis / Hepatic Stellate Cell Activation
Glucocorticoid Receptor Signaling
LPS/IL-1 Mediated Inhibition of RXR Function
Granulocyte Adhesion and Diapedesis
Hepatic Cholestasis
Role of Macrophages, Fibroblasts and Endothelial Cells in Rheumatoid Arthritis
Antiproliferative Role of TOB in T Cell Signaling
Adrenomedullin signaling pathway
BMP signaling pathway
Myc Mediated Apoptosis Signaling
Adipogenesis pathway
Induction of Apoptosis by HIV1
Osteoarthritis Pathway
Activation of IRF by Cytosolic Pattern Recognition Receptors
Mitochondrial Dysfunction
RAR Activation
Cardiomyocyte Differentiation via BMP Receptors
Oxidative Phosphorylation
Interferon Signaling
Role of Lipids/Lipid Rafts in the Pathogenesis of Influenza
HIPPO signaling
Circadian Rhythm Signaling
Docosahexaenoic Acid (DHA) Signaling
NGF Signaling
T Cell Exhaustion Signaling Pathway
ILK Signaling
Huntington's Disease Signaling
Pyridoxal 5'-phosphate Salvage Pathway
Nur77 Signaling in T Lymphocytes
Cholecystokinin/Gastrin-mediated Signaling
Mitotic Roles of Polo-Like Kinase
Role of MAPK Signaling in the Pathogenesis of Influenza
Amyotrophic Lateral Sclerosis Signaling
FXR/RXR Activation
Hypoxia Signaling in the Cardiovascular System
Cell Cycle: G2/M DNA Damage Checkpoint Regulation
Rac Signaling
Synaptogenesis Signaling Pathway
Glioma Signaling
PTEN Signaling
TGF-β Signaling
PAK Signaling
Role of IL-17F in Allergic Inflammatory Airway Diseases
VEGF Signaling
Ovarian Cancer Signaling
Glioblastoma Multiforme Signaling
STAT3 Pathway
Role of CHK Proteins in Cell Cycle Checkpoint Control
Cell Cycle Regulation by BTG Family Proteins
Role of Tissue Factor in Cancer
Prostate Cancer Signaling
Acute Myeloid Leukemia Signaling
Melanocyte Development and Pigmentation Signaling
Clathrin-mediated Endocytosis Signaling
Endocannabinoid Cancer Inhibition Pathway
Actin Cytoskeleton Signaling
FLT3 Signaling in Hematopoietic Progenitor Cells
PI3K/AKT Signaling
Bladder Cancer Signaling
IGF-1 Signaling
IL-8 Signaling
p53 Signaling
Ephrin Receptor Signaling
UVA-Induced MAPK Signaling
Regulation of Cellular Mechanics by Calpain Protease
Cancer Drug Resistance By Drug Efflux
Regulation of IL-2 Expression in Activated and Anergic T Lymphocytes
HER-2 Signaling in Breast Cancer
CDK5 Signaling
α-Adrenergic Signaling
Breast Cancer Regulation by Stathmin1
Melanoma Signaling
JAK/Stat Signaling
Agrin Interactions at Neuromuscular Junction
ErbB2-ErbB3 Signaling
Endometrial Cancer Signaling
Role of JAK1 and JAK3 in γc Cytokine Signaling
EIF2 Signaling
Estrogen-Dependent Breast Cancer Signaling
Non-Small Cell Lung Cancer Signaling
Integrin Signaling
Regulation of eIF4 and p70S6K Signaling
Hematopoiesis from Pluripotent Stem Cells
T Cell Receptor Signaling
Renal Cell Carcinoma Signaling
Apelin Endothelial Signaling Pathway
Neuregulin Signaling
IL-2 Signaling
ERK/MAPK Signaling
Insulin Receptor Signaling
Corticotropin Releasing Hormone Signaling
CNTF Signaling
Ceramide Signaling

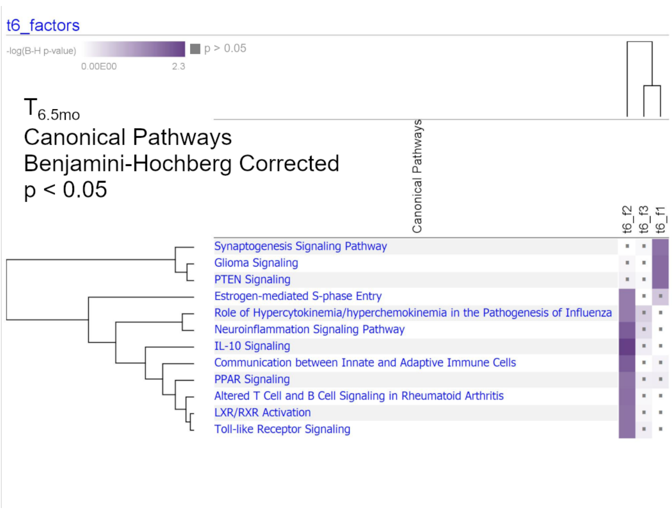
t6_factors
-log(B-H p-value)
0.00E00
2.3
p > 0.05
$T_{6.5mo}$
Canonical Pathways
Benjamini-Hochberg Corrected
p < 0.05
Canonical Pathways
t6_f2
t6_f3
t6_f1
Synaptogenesis Signaling Pathway
Glioma Signaling
PTEN Signaling
Estrogen-mediated S-phase Entry
Role of Hypercytokinemia/hyperchemokinemia in the Pathogenesis of Influenza
Neuroinflammation Signaling Pathway
IL-10 Signaling
Communication between Innate and Adaptive Immune Cells
PPAR Signaling
Altered T Cell and B Cell Signaling in Rheumatoid Arthritis
LXR/RXR Activation
Toll-like Receptor Signaling

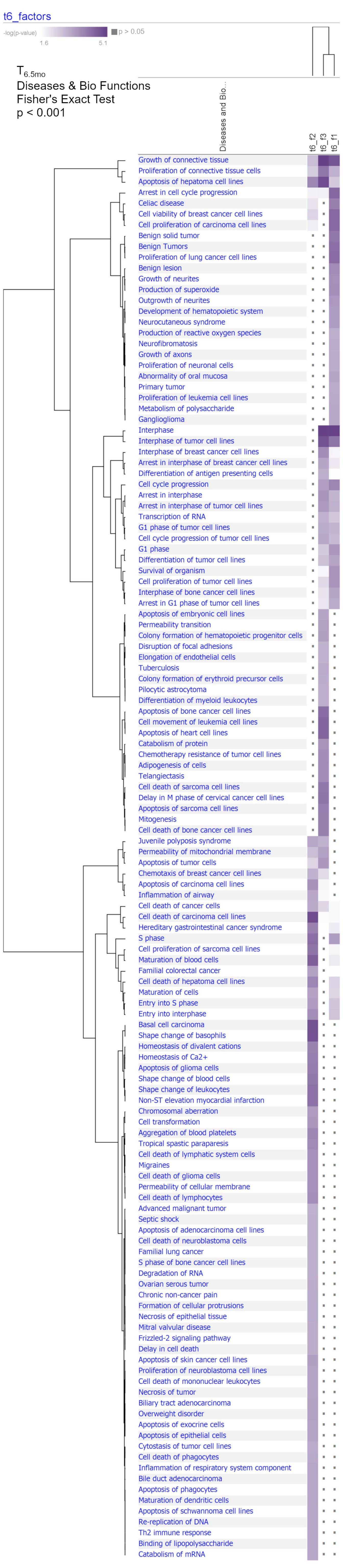

t6_factors
-log(p-value)
1.6
5.1
p > 0.05
T6.5mo
Diseases & Bio Functions
Fisher's Exact Test
p < 0.001
Diseases and Bio...
t6_f2
t6_f3
t6_f1
Growth of connective tissue
Proliferation of connective tissue cells
Apoptosis of hepatoma cell lines
Arrest in cell cycle progression
Celiac disease
Cell viability of breast cancer cell lines
Cell proliferation of carcinoma cell lines
Benign solid tumor
Benign Tumors
Proliferation of lung cancer cell lines
Benign lesion
Growth of neurites
Production of superoxide
Outgrowth of neurites
Development of hematopoietic system
Neurocutaneous syndrome
Production of reactive oxygen species
Neurofibromatosis
Growth of axons
Proliferation of neuronal cells
Abnormality of oral mucosa
Primary tumor
Proliferation of leukemia cell lines
Metabolism of polysaccharide
Ganglioglioma
Interphase
Interphase of tumor cell lines
Interphase of breast cancer cell lines
Arrest in interphase of breast cancer cell lines
Differentiation of antigen presenting cells
Cell cycle progression
Arrest in interphase
Arrest in interphase of tumor cell lines
Transcription of RNA
G1 phase of tumor cell lines
Cell cycle progression of tumor cell lines
G1 phase
Differentiation of tumor cell lines
Survival of organism
Cell proliferation of tumor cell lines
Interphase of bone cancer cell lines
Arrest in G1 phase of tumor cell lines
Apoptosis of embryonic cell lines
Permeability transition
Colony formation of hematopoietic progenitor cells
Disruption of focal adhesions
Elongation of endothelial cells
Tuberculosis
Colony formation of erythroid precursor cells
Pilocytic astrocytoma
Differentiation of myeloid leukocytes
Apoptosis of bone cancer cell lines
Cell movement of leukemia cell lines
Apoptosis of heart cell lines
Catabolism of protein
Chemotherapy resistance of tumor cell lines
Adipogenesis of cells
Telangiectasis
Cell death of sarcoma cell lines
Delay in M phase of cervical cancer cell lines
Apoptosis of sarcoma cell lines
Mitogenesis
Cell death of bone cancer cell lines
Juvenile polyposis syndrome
Permeability of mitochondrial membrane
Apoptosis of tumor cells
Chemotaxis of breast cancer cell lines
Apoptosis of carcinoma cell lines
Inflammation of airway
Cell death of cancer cells
Cell death of carcinoma cell lines
Hereditary gastrointestinal cancer syndrome
S phase
Cell proliferation of sarcoma cell lines
Maturation of blood cells
Familial colorectal cancer
Cell death of hepatoma cell lines
Maturation of cells
Entry into S phase
Entry into interphase
Basal cell carcinoma
Shape change of basophils
Homeostasis of divalent cations
Homeostasis of Ca2+
Apoptosis of glioma cells
Shape change of blood cells
Shape change of leukocytes
Non-ST elevation myocardial infarction
Chromosomal aberration
Cell transformation
Aggregation of blood platelets
Tropical spastic paraparesis
Cell death of lymphatic system cells
Migraines
Cell death of glioma cells
Permeability of cellular membrane
Cell death of lymphocytes
Advanced malignant tumor
Septic shock
Apoptosis of adenocarcinoma cell lines
Cell death of neuroblastoma cells
Familial lung cancer
S phase of bone cancer cell lines
Degradation of RNA
Ovarian serous tumor
Chronic non-cancer pain
Formation of cellular protrusions
Necrosis of epithelial tissue
Mitral valvular disease
Frizzled-2 signaling pathway
Delay in cell death
Apoptosis of skin cancer cell lines
Proliferation of neuroblastoma cell lines
Cell death of mononuclear leukocytes
Necrosis of tumor
Biliary tract adenocarcinoma
Overweight disorder
Apoptosis of exocrine cells
Apoptosis of epithelial cells
Cytostasis of tumor cell lines
Cell death of phagocytes
Inflammation of respiratory system component
Bile duct adenocarcinoma
Apoptosis of phagocytes
Maturation of dendritic cells
Apoptosis of schwannoma cell lines
Re-replication of DNA
Th2 immune response
Binding of lipopolysaccharide
Catabolism of mRNA

t6_factors

T$_{6.5mo}$
Diseases & Bio Functions
Benjamini-Hochberg Corrected
p < 0.05

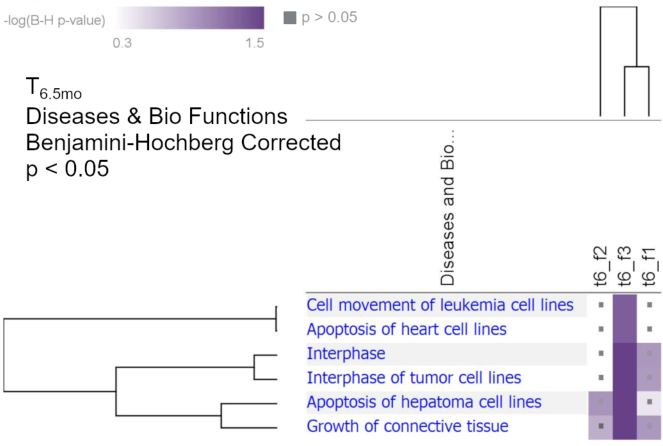

Supplement: Supplementary file 7 — Supplementary Information 7. [file 41598_2020_80734_MOESM7_ESM.pdf]
