# Supplementary material for: Infant circulating MicroRNAs as biomarkers of effect in fetal alcohol spectrum disorders
Source: Sci Rep. 2021 Jan 14;11:1429. doi: 10.1038/s41598-020-80734-y (PMC7809131; doi:10.1038/s41598-020-80734-y)
Supplement: Supplementary file 10 — Supplementary Information 10. [file 41598_2020_80734_MOESM10_ESM.pdf]

Supplementary Methods S10

Omega Adjustment for Collinearity in the Residuals

The formula for Omega with correlated residuals was adjusted as per recommendations of (1) and is shown below:

$$Omega = \frac{(\sum \lambda_i)^2}{(\sum \lambda_i)^2 + \sum Var(\varepsilon_i) + 2\sum_{\iota}\sum_j Var_{ij}} \qquad \text{(Equation 2b)}$$

With the term $2*\Sigma_i\Sigma_j var_{ij}$ being two times the sum of the covariance between the error terms.

Fit Indices and Corrective Statistics for Sample Size per CFA Model

A number of additional fit indices along with corrected statistics as per Bartlett, Yuan and Swain were implemented to further enrich the findings of the CFA models. An R function was developed for that purpose. The findings are shown in the supplemental figures showing the magnitude of the fit indices in relation to what constitutes acceptable model fit (e.g., fit indices >.90, RMSE<.05, Chi-square/D.F.<2, etc.).

Enrichment of pathway targeting for additional factor miRNAs

Following IPA analysis of the factor loaded miRNAs, we examined additional miRNAs with an effect size > 0.35 to determine if there was a biological basis for the inclusion of additional miRNAs in the factors. Using the results from the IPA, we identified the unique, discriminating biological pathways which contained mRNAs targeted by the miRNAs in each factor. We then assessed each of the candidate additional miRNAs for enriched targeting of these discriminating pathways. An enrichment score was created by quantifying the number of times each candidate miRNA targeted mRNAs within the discriminating factor pathways divided by the number of times mRNAs in those pathways were targeted by all the miRNAs. miRNAs which showed enrichment for

targeting unique factor pathways were then added into the original factors if they improved the model fit.

Bootstrap resampling method

To test the stability of the pairwise correlations with *p*-values less than 0.05, we used a bootstrap method (2-4) and resampled each of the groups (PAE and controls) by replacement to generate a new resampled group with sample size equal to the original group. We then generated a *p*-value matrix for the resampled groups and determined the number of significant *p*-values. The resampling method was repeated for 2000 iterations for each of the groups. Confidence intervals for the difference between the mean number of significant *p*-values in the control and PAE groups were generated as well as the standard deviation of the distribution. Bootstrap resampling with replacement, with 2000 iterations, was also used to assess potential sex differences in $_{ex}$miRNA expression. $_{ex}$miRNAs were quantified as “likely sex-specific” if they were significant in at least 50% of the resampling analyses (proportion >0.5) and were significantly altered more frequently in one infant sex than the population as a whole.

R Code for Correlation Plots and Bootstrap analysis

capetown_knitr.R

```
#read the data tables
merged_t0_ctrl <- read.csv("C:/Users/nihal/Desktop/Cape_town_R_scripts
/merged_t0_ctrl.csv")
merged_t0_eth <- read.csv("C:/Users/nihal/Desktop/Cape_town_R_scripts/
merged_t0_eth.csv")
merged_t6_ctrl <- read.csv("C:/Users/nihal/Desktop/Cape_town_R_scripts
/merged_t6_ctrl.csv")
merged_t6_eth <- read.csv("C:/Users/nihal/Desktop/Cape_town_R_scripts/
merged_t6_eth.csv")
mimat_loc_t0 <- read.csv("C:/Users/nihal/Desktop/Cape_town_R_scripts/t
```

```
0_miRNA_location.csv")
mimat_loc_t6<- read.csv("C:/Users/nihal/Desktop/Cape_town_R_scripts/t6
_miRNA_location.csv")
#subset the data to have the 148 miRNA columns only
cor_miR_t0_ctrl<-merged_t0_ctrl[,-c(1,150:159)]
cor_miR_t0_eth<-merged_t0_eth[,-c(1,150:159)]
cor_miR_t6_ctrl<-merged_t6_ctrl[,-c(1:2,151:160)]
cor_miR_t6_eth<-merged_t6_eth[,-c(1:2,151:160)]
#example of the head of the tables (the first 6 columns only for the w
idth of the document)
head(cor_miR_t0_ctrl[,1:6])
```

```
##   MIMAT0000252_t0 MIMAT0000752_t0 MIMAT0000435_t0 MIMAT0000431_t0 M
IMAT0000267_t0 MIMAT0000460_t0
## 1          4.5181         10.4978          1.9758          0.1056
6.8282          0.7856
## 2          3.2249          1.8634          0.6327          9.2682
7.9745          2.9407
## 3          3.1290          0.8669          0.8505          0.9307
7.6617          1.0695
## 4          6.4971          2.7475          0.9660          1.5122
7.6501          1.4382
## 5          3.6535          2.3416          2.0921          2.3006
0.5507          1.4123
## 6          2.2661          1.9001          3.2532          2.3976
7.2232          1.0592
```

```
#use the corrplot library for generating the plots
library(corrplot)
#function to genrate a matrix of p values for the correlation
cor.mtest <- function(mat, ...) {
  mat <- as.matrix(mat)
  n <- ncol(mat)
  p.mat<- matrix(NA, n, n)
  diag(p.mat) <- 0
  for (i in 1:(n - 1)) {
    for (j in (i + 1):n) {
      tmp <- cor.test(mat[, i], mat[, j], ...)
      p.mat[i, j] <- p.mat[j, i] <- tmp$p.value
    }
  }
  colnames(p.mat) <- rownames(p.mat) <- colnames(mat)
  p.mat
}
#generate the correlation matrices and p value matrices for each of th
e t0 and t6 ,control and ethanol data sets
```

```
M0_ctrl<-cor(cor_miR_t0_ctrl)
p.mat0_ctrl <- cor.mtest(cor_miR_t0_ctrl)
M0_eth<-cor(cor_miR_t0_eth)
p.mat0_eth <- cor.mtest(cor_miR_t0_eth)
M6_ctrl<-cor(cor_miR_t6_ctrl)
p.mat6_ctrl <- cor.mtest(cor_miR_t6_ctrl)
M6_eth<-cor(cor_miR_t6_eth)
p.mat6_eth <- cor.mtest(cor_miR_t6_eth)
#generate the plots ,correlations with p values higher than 0.05 are m
asked (white), the miRNAs are ordered by heirarcheal clustering method
corrplot(M0_ctrl, type="upper", order="hclust",
         p.mat = p.mat0_ctrl,addgrid.col=NA ,col=colorRampPalette(c("r
ed","white","blue"))(200), sig.level = 0.05,insig = "blank")
text(0,25,"ctrl_t0_hc_0.05")

corrplot(M0_eth, type="upper", order="hclust",
         p.mat = p.mat0_eth ,col=colorRampPalette(c("red","white","blu
e"))(200),addgrid.col=NA, sig.level = 0.01,insig = "blank")
text(0,25,"eth_t0_hc_0.05")

corrplot(M6_ctrl, type="upper", order="hclust",
         p.mat = p.mat6_ctrl,addgrid.col=NA ,col=colorRampPalette(c("r
ed","white","blue"))(200), sig.level = 0.05,insig = "blank")
text(0,25,"ctrl_t6_hc_0.05")

corrplot(M6_eth, type="upper", order="hclust",
         p.mat = p.mat6_eth ,col=colorRampPalette(c("red","white","blu
e"))(200),addgrid.col=NA, sig.level = 0.05,insig = "blank")
text(0,25,"eth_t6_hc_0.05")

#now we need to generate correlation plots ordered by the chromosomal
location of the miRNAs,
#this was done by ordering the miRNAs by location in Excel sheet and i
mport the ordered miRNAs list
#order my data frame by this ordered list
#this is how the mimat loc table looks like
head(mimat_loc_t0[1:5])

##     X        Mimat time.point .loc type.of.trans.
## 1 146 MIMAT0004557          0    1          miRNA
## 2  99 MIMAT0000692          0    1          miRNA
## 3  42 MIMAT0000227          0    1          miRNA
## 4  59 MIMAT0000271          0    1          miRNA
## 5  54 MIMAT0000256          0    1          miRNA
## 6  98 MIMAT0000681          0    1          miRNA
```

```
miR_t0_ctrl_ordered_loc<-cor_miR_t0_ctrl[,as.character(mimat_loc_t0$Va
riables)]
miR_t0_eth_ordered_loc<-cor_miR_t0_eth[,as.character(mimat_loc_t0$Vari
ables)]
head(miR_t0_ctrl_ordered_loc[,1:6])
```

```
##   MIMAT0004557_t0 MIMAT0000692_t0 MIMAT0000227_t0 MIMAT0000271_t0 M
IMAT0000256_t0 MIMAT0000681_t0
## 1         13.4187         -0.4159          2.9601          2.7811
0.4927          3.6452
## 2          8.6539          8.8862          0.7172          2.2490
0.0365          2.7242
## 3          4.6561         -0.3431          1.3366          1.7890
0.1961          2.3996
## 4         10.9144         -0.0465          1.8632          2.5919
0.3997          1.5562
## 5          3.5321          0.6047          1.2586          2.6532
1.0020          3.2530
## 6          0.3721          0.5306          1.1325          3.0136
-0.1167          3.1200
```

```
#correlation amtrix for t0 ctrl and ethanol ordered by location
M0_ctrl_loc<-cor(miR_t0_ctrl_ordered_loc)
p.mat0_ctrl_loc <- cor.mtest(miR_t0_ctrl_ordered_loc)
#import the chromosome number list to use it to color the miRNA by chr
omosomal location
#substiute X with a number 50 (just to generate a color for it)
color_t0_scheme<-gsub("X","50",mimat_loc_t0$.loc)
corrplot(M0_ctrl_loc, type="upper",
         p.mat = p.mat0_ctrl_loc ,col=colorRampPalette(c("red","white"
,"blue"))(200),tl.col=color_t0_scheme,addgrid.col=NA, sig.level = 0.05
,insig = "blank")#ethanol
```

```
M0_eth_loc<-cor(miR_t0_eth_ordered_loc)
p.mat0_eth_loc <- cor.mtest(miR_t0_eth_ordered_loc)
color_t0_scheme<-gsub("X","50",mimat_loc_t0$.loc)
corrplot(M0_eth_loc, type="upper",
         p.mat = p.mat0_eth_loc ,col=colorRampPalette(c("red","white",
"blue"))(200),tl.col=color_t0_scheme,addgrid.col=NA, sig.level = 0.05,
insig = "blank")
```

```
##location t 6
miR_t6_ctrl_ordered_loc<-cor_miR_t6_ctrl[,as.character(mimat_loc_t6$Va
riables)]
miR_t6_eth_ordered_loc<-cor_miR_t6_eth[,as.character(mimat_loc_t6$Vari
ables)]
head(miR_t6_ctrl_ordered_loc[,1:6])
```

```
##   MIMAT0004557_t6 MIMAT0000692_t6 MIMAT0000227_t6 MIMAT0000271_t6 M
IMAT0000256_t6 MIMAT0000681_t6
## 1          4.2643         -0.2922          1.5974          2.7194
2.5401          1.7337
## 2         11.2952          0.9414          1.1273          2.4208
-1.1288          0.4227
## 3         13.2387         -1.7551          1.5340          2.1855
0.3371          9.3907
## 4         13.2172         -2.4298          1.6701          2.9012
-1.0201          0.5069
## 5          2.4228         -1.5197          0.6041          1.5140
-1.8955          0.1729
## 6         11.5225         -1.9411          1.6495          2.9199
10.7730          0.7495

#correlation amtrix for t6 ctrl and ethanol ordered by location
M6_ctrl_loc<-cor(miR_t6_ctrl_ordered_loc)
p.mat6_ctrl_loc <- cor.mtest(miR_t6_ctrl_ordered_loc)
color_t6_scheme<-gsub("X","50",mimat_loc_t6$.loc)
corrplot(M6_ctrl_loc, type="upper",
         p.mat = p.mat6_ctrl_loc ,col=colorRampPalette(c("red","white"
,"blue"))(200),tl.col=color_t6_scheme,addgrid.col=NA, sig.level = 0.01
,insig = "blank")

#ethanol
M6_eth_loc<-cor(miR_t6_eth_ordered_loc)
p.mat6_eth_loc <- cor.mtest(miR_t6_eth_ordered_loc)
color_t6_scheme<-gsub("X","50",mimat_loc_t6$.loc)
corrplot(M6_eth_loc, type="upper",
         p.mat = p.mat6_eth_loc ,col=colorRampPalette(c("red","white",
"blue"))(200),tl.col=color_t0_scheme,addgrid.col=NA, sig.level = 0.01,
insig = "blank")

#################
#the code for generating correlation matrices for miRNAs with effect s
izes more then 0.4
#read the table of miRNA with high effect sizes , ordered by location
and convert the variables column to character instead of factor and su
bset your intial data matrices
#to include only the high effect size miRNAs
t0_0_4_location <- read.csv("C:/Users/nihal/Desktop/Cape_town_R_script
s/t0_0.4_location.csv")
L<-as.character(t0_0_4_location$Variables)
#how the table looks like
head(t0_0_4_location)
```

```
##         Variables .loc     start       end absolute.effect.size_t0
## 1 MIMAT0005867_t0    2 133014543 133014564               0.4680125
## 2 MIMAT0004911_t0    5 136983271 136983292               0.4749076
## 3 MIMAT0000435_t0    5 148808541 148808561               0.4049864
## 4 MIMAT0000437_t0    5 148810224 148810246               0.4808910
## 5 MIMAT0000093_t0    7  99691438  99691460               0.5848045
## 6 MIMAT0003266_t0    8  10892731  10892752               0.4202743
```

```
high_ctrl_t0<-(cor_miR_t0_ctrl[,L])
high_eth_t0<-cor_miR_t0_eth[,L]
#just changing the underscore to hyphen for readable plot
names(high_ctrl_t0) = sub("_","-",names(high_ctrl_t0))
names(high_eth_t0) = sub("_","-",names(high_eth_t0))
#generate correlation matrices and p value matrices for the subsetted
datasets
M_ctrl_t0_high<-cor(high_ctrl_t0)
p.mat.ctrl.t0.high<-cor.mtest(high_ctrl_t0)
M_eth_t0_high<-cor(high_eth_t0)
p.mat.eth.t0.high<-cor.mtest(high_eth_t0)
#color scheme for the ordered by location plots
color_t0_scheme_high<-gsub("X","20",t0_0_4_location$.loc)
#generate the plots
#ordered by heirarcheal clustering
#set the self-diagonal correlation to zero

M_ctrl_t0_high[which(M_ctrl_t0_high==1)]<-0

M_eth_t0_high[which(M_eth_t0_high==1)]<-0


corrplot(M_ctrl_t0_high, type="upper", order="hclust",
         p.mat = p.mat.ctrl.t0.high,addgrid.col=NA,col=colorRampPalett
e(c("red","white","blue"))(200),tl.cex=0.8, sig.level = 0.05,insig = "
blank")
text(2,5,"ctrl_t0_high_hc_0.05")

corrplot(M_eth_t0_high, type="upper", order="hclust",
         p.mat = p.mat.eth.t0.high,addgrid.col=NA,col=colorRampPalette
(c("red","white","blue"))(200), sig.level = 0.05,tl.cex=0.8,insig = "b
lank")
text(3,5,"eth_t0_high_hc_0.05")

#ordered by chromosomal location #note: the high effect size miRNA lis
t was ordered bylocation and the main dataframe was subset using this
list so
#it is already ordered by location #deleting order="hclust" uses the o
riginal order from the dataframe
```

```
corrplot(M_ctrl_t0_high, type="upper",
         p.mat = p.mat.ctrl.t0.high,addgrid.col=NA,tl.col=color_t0_sch
eme_high,col=colorRampPalette(c("red","white","blue"))(200),tl.cex=0.8
, sig.level = 0.05,insig = "blank")
text(2,5,"ctrl_t0_high_loc_0.05")

corrplot(M_eth_t0_high, type="upper",
         p.mat = p.mat.eth.t0.high,addgrid.col=NA,tl.col=color_t0_sche
me_high,col=colorRampPalette(c("red","white","blue"))(200), sig.level
= 0.05,insig = "blank",tl.cex=0.8)
text(3,5,"eth_t0_high_loc_0.05")

#the same approach for t6
t6_0_4_location <- read.csv("C:/Users/nihal/Desktop/Cape_town_R_script
s/t6_0.4_location.csv")
L6<-as.character(t6_0_4_location$Variables)
high_ctrl_t6<-cor_miR_t6_ctrl[,L6]
high_eth_t6<-cor_miR_t6_eth[,L6]
names(high_ctrl_t6) = sub("_","-",names(high_ctrl_t6))
names(high_eth_t6) = sub("_","-",names(high_ctrl_t6))
color_t6_scheme_high<-gsub("X","20",t6_0_4_location$.loc)
head(high_ctrl_t6[,1:5])

##   MIMAT0000414-t6 MIMAT0000069-t6 MIMAT0000435-t6 MIMAT0000449-t6 M
IMAT0000101-t6
## 1         -1.2730         -8.1441          2.9555         -0.8325
-1.8393
## 2         -1.9767         -7.8664          0.4462         -1.0456
-1.7870
## 3         -1.5055         -7.3059         -0.3621         -1.1795
-2.1211
## 4         -2.6283          3.4625          0.7590          1.4032
-1.5331
## 5         -2.9983         -8.4687          1.3157         -1.6338
-2.0600
## 6         -2.1522         -8.1768          0.0706         -1.3111
-1.4434

M_ctrl_t6_high<-cor(high_ctrl_t6)
p.mat.ctrl.t6.high<-cor.mtest(high_ctrl_t6)
M_eth_t6_high<-cor(high_eth_t6)
p.mat.eth.t6.high<-cor.mtest(high_eth_t6)

M_ctrl_t6_high[which(M_ctrl_t6_high==1)]<-0

M_eth_t6_high[which(M_eth_t6_high==1)]<-0
```

```
corrplot(M_ctrl_t6_high, type="upper", order="hclust",
         p.mat = p.mat.ctrl.t6.high,addgrid.col=NA,col=colorRampPalett
e(c("red","white","blue"))(200), sig.level = 0.05,insig = "blank",tl.c
ex=1.2)
text(2,5,"ctrl_t6_hc_high_0.05")

corrplot(M_ctrl_t6_high, type="upper",
         p.mat = p.mat.ctrl.t6.high,addgrid.col=NA,tl.col=color_t6_sch
eme_high,col=colorRampPalette(c("red","white","blue"))(200),tl.cex=1.2
, sig.level = 0.05,insig = "blank")
text(2,5,"ctrl_t6_high_loc_0.05")

corrplot(M_eth_t6_high, type="upper", order="hclust",
         p.mat = p.mat.eth.t6.high,addgrid.col=NA,col=colorRampPalette
(c("red","white","blue"))(200),tl.cex=1.2, sig.level = 0.05,insig = "b
lank")
text(2,5,"eth_t6_hc_high_0.05")

corrplot(M_eth_t6_high, type="upper",
         p.mat = p.mat.eth.t6.high,addgrid.col=NA,tl.col=color_t6_sche
me_high,col=colorRampPalette(c("red","white","blue"))(200),tl.cex=1.2,
sig.level = 0.05,insig = "blank")
text(2,5,"eth_t6_loc_high_0.05")

##Calculating the number of significant p values (less than 0.05) in e
ach of the groups (t0 control ,t0 ethanol,t6 control , t6 ethanol)
length(which(p.mat0_ctrl<0.05))

## [1] 3066

length(which(p.mat0_eth<0.05))

## [1] 6458

length(which(p.mat6_ctrl<0.05))

## [1] 3592

length(which(p.mat6_eth<0.05))

## [1] 4252

## to test the stability of number of significant p values in each of
the four groups
#we applied bootstrap to simulate a population from the sample set we
have
#in each simulated sample we generated p values matric and counted the
number of significant correlations
```

```
#after 2000 simulations , histogram was drown and t test was conducted between the two lists containing number of significant p values in each
#simulated run
#for the purpose of generating this report , we are running the code for 50 iterations , however the reported data was from 2000 iterations
pmat_ctrl_0_boot_sig<-list()
pmat_eth_0_boot_sig<-list()
for(i in 1:50) {
  row_C_0 <- sample(row(cor_miR_t0_ctrl),nrow(cor_miR_t0_ctrl), replace = TRUE)
  c_0<-cor_miR_t0_ctrl[row_C_0,]
  pmat_c_0<-cor.mtest(c_0)
  pmat_ctrl_0_boot_sig[i]<-length(which(pmat_c_0<0.05))

  row_E_0 <- sample(row(cor_miR_t0_eth),nrow(cor_miR_t0_eth), replace = TRUE)
  E_0<-cor_miR_t0_eth[row_E_0,]
  pmat_E_0<-cor.mtest(E_0)
  pmat_eth_0_boot_sig[i]<-length(which(pmat_E_0<0.05))


}
par(mfrow=c(3,1))
hist(unlist(pmat_ctrl_0_boot_sig),col="blue",xlim=c(2000,12000),breaks=5,main="ctrl blue ,eth red, t0")
hist(unlist(pmat_eth_0_boot_sig),col="red",breaks=5,xlim=c(2000,12000))
hist(unlist(pmat_ctrl_0_boot_sig),col="blue",xlim=c(2000,12000),breaks=5,main="ctrl blue ,eth red, t0")
hist(unlist(pmat_eth_0_boot_sig),col="red",breaks=5,add=TRUE)
```

```
t.test(unlist(pmat_ctrl_0_boot_sig),unlist(pmat_eth_0_boot_sig))
```

```
##
##  Welch Two Sample t-test
##
## data:  unlist(pmat_ctrl_0_boot_sig) and unlist(pmat_eth_0_boot_sig)
## t = -11.449, df = 91.495, p-value < 2.2e-16
## alternative hypothesis: true difference in means is not equal to 0
## 95 percent confidence interval:
##  -3442.412 -2424.548
## sample estimates:
## mean of x mean of y
##   5299.60   8233.08
```

```r
#t6
pmat_ctrl_6_boot_sig<-list()
pmat_eth_6_boot_sig<-list()
for(i in 1:50) {
  row_C_6 <- sample(row(cor_miR_t6_ctrl),nrow(cor_miR_t6_ctrl), replace = TRUE)
  c_6<-cor_miR_t6_ctrl[row_C_6,]
  pmat_c_6<-cor.mtest(c_6)
  pmat_ctrl_6_boot_sig[i]<-length(which(pmat_c_6<0.05))

  row_E_6 <- sample(row(cor_miR_t6_eth),nrow(cor_miR_t6_eth), replace = TRUE)
  E_6<-cor_miR_t6_eth[row_E_6,]
  pmat_E_6<-cor.mtest(E_6)
  pmat_eth_6_boot_sig[i]<-length(which(pmat_E_6<0.05))


}
par(mfrow=c(3,1))
hist(unlist(pmat_ctrl_6_boot_sig),col="blue",xlim=c(2000,12000),breaks=5,main="ctrl blue ,eth red, t6")
hist(unlist(pmat_eth_6_boot_sig),col="red",breaks=5,xlim=c(2000,12000))
hist(unlist(pmat_ctrl_6_boot_sig),col="blue",xlim=c(2000,12000),breaks=5,main="ctrl blue ,eth red, t6")
hist(unlist(pmat_eth_6_boot_sig),col="red",breaks=5,add=TRUE)
```

Supplemental References

1. Wang J, Wang X. Structural Equation Modeling: Applications Using Mplus: John Wiley & Sons; 2012.
2. Efron B. Bootstrap Methods: Another Look at the Jackknife. Ann Statist. 1979;7(1):1-26.
3. Kohavi R. A study of cross-validation and bootstrap for accuracy estimation and model selection. Proceedings of the 14th international joint conference on Artificial intelligence - Volume 2; Montreal, Quebec, Canada. 1643047: Morgan Kaufmann Publishers Inc.; 1995. p. 1137-43.
4. Salem NA, Mahnke AH, Wells AB, Tseng AM, Yevtushok L, Zymak-Zakutnya N, et al. Association between fetal sex and maternal plasma microRNA responses to prenatal alcohol exposure: evidence from a birth-outcomes stratified cohort. Biol Sex Differ. 2020; *Accepted*.
