# Supplementary material for: Infant circulating MicroRNAs as biomarkers of effect in fetal alcohol spectrum disorders
Source: Sci Rep. 2021 Jan 14;11:1429. doi: 10.1038/s41598-020-80734-y (PMC7809131; doi:10.1038/s41598-020-80734-y)
Supplement: Supplementary file 12 — Supplementary Information 12. [file 41598_2020_80734_MOESM12_ESM.docx]

# Supplementary Information

**Supplementary Figure S1**

**Methods to determine plasma purity.** (a) Free hemoglobin, as measured by absorbance at 414nm, was elevated in T_2wk_ plasma samples. (b) Enrichment of miR-451a (MIMAT0001631) relative to miR-23a (MIMAT0000078; ΔCT_miR23a-miR451a_) is not significantly altered between timepoints and was less than 7, the threshold for erythrocyte contamination^[43]^, for each group. (c) Previous reports of a linear relation between absorbance at 414nm and ΔCT_(miR23a-miR451a)_^[105]^ were not replicated in these plasma samples obtained during early infancy, suggesting that these parameters in infants may not be robust indicators of contamination, perhaps due to elevated levels of erythrocyte destruction in in infant liver resulting in hemoglobin release into circulation. Samples showing both ΔCT > 7 and elevated 414nm absorbance (>0.3) were excluded from the analysis (yellow region). (d) All assessed plasma samples used for *_ex_*miRNA analysis had no detectable level of SLC4A1 mRNA, further confirming that hemoglobin release was not due to contamination from erythrocyte damage during sample preparation. (e) There were no appreciable quantitative real-time PCR inhibitors present, as increasing levels of RNA input did not affect the amplification of spike-in control cel-miR-39-3p, particularly around the 25 ng RNA input used in this study.

**Supplementary Table S2**

**Effect size estimates and parametric significance testing of *_ex_*miRNA expression.**

**Supplementary Figure S3**

**Correlation of *_ex_*miRNA expression across chromosomes.** Correlation plots ordered by chromosomal location of *_ex_*miRNAs for T_2wk_ control, T_2wk_ PAE, T_6.5mo_ control, and T_6.5mo_ PAE infant *_ex_*miRNAs. Genomic loci for duplicated miRNAs denoted with an underscore and number for the duplicated loci, as denoted in the miRbase database. Figures were constructed using the corrplot package (version 0.77, https://cran.r-project.org/web/packages/corrplot/index.html) for R (version 3.6.1).

**Supplementary Table S4**

**Chromosomal miRNA cluster membership of *_ex_*miRNAs with effect size ≥ 0.40.**

**Supplementary Figure S5**

**IPA Comparison Analysis pathways for T_2wk_.** Figures were constructed using the Canonical Pathway and Disease and Biological Functions analyses in the Ingenity Pathway Analysis (Winter 2019 update, https://digitalinsights.qiagen.com/products-overview/discovery-insights-portfolio/analysis-and-visualization/qiagen-ipa/).

**Supplementary Figure S6**

**Descriptive fit indices and corrected statistics as per Bartlett’s, Yuan’s, and Swain’s corrections.** (a) Fit indices and corrected statistics at T_2wk_. (b) Parsimony indices for T_2wk_ have recommended values of .50, descriptive indices .90, RMSEA, <.05, and Chi-square to D.F. ratio <2.0. (c) Fit indices and corrected statistics at T_6.5mo_. (d) Parsimony indices for T_6.5mo_ have recommended values of .50, descriptive indices .90, RMSEA, <.05, and Chi-square to D.F. ratio <2.0.

**Supplementary Figure S7**

**IPA Comparison Analysis pathways for T_6.5mo_.** Figures were constructed using the Canonical Pathway and Disease and Biological Functions analyses in the Ingenuity Pathway Analysis (Winter 2019 update, https://digitalinsights.qiagen.com/products-overview/discovery-insights-portfolio/analysis-and-visualization/qiagen-ipa/).

**Supplementary Table S8**

**Comparison of PAE-affected *_ex_*miRNAs with previous studies.**

**Supplementary Table S9**

**Factor loaded cirmiRNAs implicated as potentially sex-specific.**

**Supplementary Methods S10**

**Supplemental methods.**

**Supplementary Dataset 11**

**Infant Circulating miRNA Expression Data**
